# Non-solvating additives regulate zinc deposition through anion-gated Stern-layer competition

**DOI:** 10.1038/s41467-026-76659-1

**Published:** 2026-08-13

**Authors:** Li Li, Hang Yang, Tengyu Yao, Yiming Zhang, Yicheng Tan, Chenglin Miao, Duo Chen, Jingyi Wang, Yunpeng Zhong, Xingsen Guo, Jianrui Feng, Adham Hashibon, Guangshe Li, Wei Han, Guanjie He

**Affiliations:** 1 College of Physics, State Key Laboratory of Inorganic Synthesis and Preparative Chemistry, International Center of Future Science, Jilin University, Changchun, China; 2 Christopher Ingold Laboratory, Department of Chemistry, University College London, London, UK; 3 Jiangsu Key Laboratory of Electrochemical Energy Storage Technologies, College of Materials Science and Technology, Nanjing University of Aeronautics and Astronautics, Nanjing, China; 4 Department of Civil, Environmental and Geomatic Engineering, University College London, London, UK; 5 Institute for Materials Discovery, University College London, London, UK; 6 State Key Laboratory of Inorganic Synthesis and Preparative Chemistry, College of Chemistry, Jilin University, Changchun, China

**Keywords:** Batteries, Batteries

## Abstract

While additive-induced modulation of solvation structure has emerged as an effective strategy to enhance the performance of aqueous zinc-ion batteries, the electrochemical mechanisms by which non-solvation additives interact with ion species within the electric double layer and their subsequent impact on solid electrolyte interphase evolution remain poorly understood. Here, we propose an anion-released interfacial engineering strategy that leverages the competitive spatial distribution between surface-affinitive anions and cationic regulators within the Stern layer. This competition governs additive accessibility to the interface and enables the construction of a robust, inorganic–organic hybrid interface. Combined with in situ spectra and theoretical simulations, we decouple the key kinetic processes in Zn deposition, revealing that fast Zn^2+^ transport within the solid electrolyte interphase, coupled with moderated desolvation at the interface, underpins dendrite-free and highly reversible cycling. This study has the potential to establish a mechanistic framework for the interfacial function of non-solvating additives, thus offering refined insights into electrolyte design and interphase engineering for high-performance aqueous metal batteries.

## Abstract (web summary)

Aqueous zinc-ion batteries can benefit from electrolyte additives, but their interfacial mechanisms remain unclear. Here, authors explore tetraethylammonium-based salts as functional additives and investigate their non-solvating nature to regulate ion distributions at the interface to form a robust interphase, enabling reversible and long-lived zinc batteries.

## Introduction

Zn metal is an ideal negative electrode material for aqueous batteries due to its cost-effectiveness, moderate redox potential (−0.763 V vs. standard hydrogen electrode, SHE), and high theoretical volumetric capacity (5855 mAh cm^−3^)^1,2^. However, Zn dendrites and side reactions such as hydrogen evolution and corrosion inherent in aqueous electrolytes impede the widespread commercialization of aqueous zinc-ion batteries^3,4^. Among mitigation strategies, electrolyte engineering has gained traction for its operational simplicity and effectiveness in stabilizing Zn electrodes^4–6^.

**Fig. 1:**
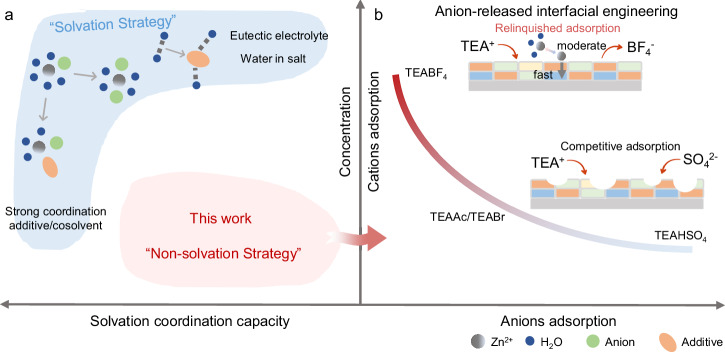
Mechanisms of electrolyte modification. **a** The schematic diagram depicting the solvation structure in conventional electrolyte engineering and **b** the mechanism of the synergistic regulatory effect between cations and anions in the non-solvation additives in this work.

In recent years, electrolyte engineering has focused predominantly on modulating the Zn^2+^ solvation structure to improve Zn negative electrode stability. Highly concentrated salts^7^, water-in-salt systems^8,9^, asymmetric anion clusters^10^, and various organic/ionic-liquid cosolvents^11,12^ have been shown to reduce water activity, strengthen anion coordination, or rearrange the primary solvation shell (Fig. 1a). These solvation-based strategies have undeniably advanced the field and are now widely used to rationalize enhancements in Coulombic efficiency (CE), dendrite suppression, and solid-electrolyte interphase (SEI) formation. Yet, a critical blind spot remains. Not all performance improvements originate from solvation restructuring. Many electrolyte additives are employed at concentrations far below the threshold required to modify the Zn^2+^ solvation shell or the bulk hydrogen-bond network, but still show pronounced effects on interfacial morphology and long-term Zn plating/stripping^13^. The mechanistic origin of such improvements is often interpreted, even sometimes implicitly, under the solvation framework, despite lacking direct evidence of any solvation change. This reveals a gap in current understanding: the field lacks a conceptual basis for interpreting the behavior of non-solvating additives, i.e., species that do not enter the solvation shell yet exert a strong influence on interfacial behavior. Their effect is comparable to the interfacial perturbation mechanism realized through the construction of artificial interface layers and the optimization of substrate properties^14^. Therefore, here we establish an interface regulation mechanism departs the solvation-centric view. We show that trace-level, non-solvating electrolyte additives can regulate Zn deposition not by altering bulk solvation, but by selectively accessing the Stern layer and participating in interfacial assembly.

Quaternary amine salt compounds are frequently employed as functional additives in aqueous zinc-based battery systems^15,16^. The nitrogen atoms possessing lone electron pairs in these compounds can establish robust interactions with zinc atoms, thus constructing specific interface layers, effectively suppressing the formation of alkaline by-products and the hydrogen evolution reaction (HER), and optimizing the deposition behavior of Zn^2+^. Although a multitude of studies have elucidated the regulatory mechanism of such additives at the electrode interface, there are relatively few research efforts that focus on the critical role played by their counterions.

In this work, using a family of quaternary amine salts containing tetraethylammonium and different anions (TEA^+^X^−^, where X^−^ = BF_4_^−^, HSO_4_^−^, CH_3_COO^−^, and Br^−^), we demonstrate that at 10 mmol L^−1^ (10 mM), the Zn^2+^ solvation shell, water structure, and bulk electrolyte speciation remain unchanged, as confirmed by molecular dynamics (MD) simulations and diverse spectral detection. Instead, whether an additive can influence Zn deposition is determined by an entirely different factor: the competitive occupation of the Stern layer between the functional cation and its counter-anion. Different anions possess distinct intrinsic affinities toward the zinc electrode. By substituting the strongly-adsorbing anions with weakly surface-oriented anions, the distribution density of TEA^+^ in the Stern layer was effectively enhanced, thus facilitating the formation of a high-quality SEI rich in TEA^+^ (Fig. 1b). Moreover, based on in situ distribution of relaxation times (DRT) analysis, we elucidated the distinct roles of various SEI components and electric double layer (EDL) structure in affecting the zinc deposition behavior. This study establishes a clear correlation between the competition distribution behavior of additive and the evolution of the electrode-electrolyte layer (EEL) chemistry along with functional SEI formation. The analytical approach presented here also offers broad applicability in understanding the mechanisms underlying other promising electrolyte engineering strategies.

## Results

### Non-solvating additives govern Zn deposition

The nitrogen atom in quaternary ammonium groups has been reported to exhibit electrophilic characteristics, enabling potential interactions with Zn^2+^ as both an electron acceptor and nucleophilic site^15^. Building on this insight, we hypothesized that the TEA^+^ could potentially regulate the deposition behavior of Zn^2+^. To test this, we introduced 10 mM TEA^+^-based salts (TEABF_4_, TEAHSO_4_, TEABr, and TEACH_3_COO) into the 2 M ZnSO_4_ electrolyte, denoted as ZS + TEABF, ZS + TEAHS, ZS + TEABr, and ZS + TEAAc, respectively.

**Fig. 2:**
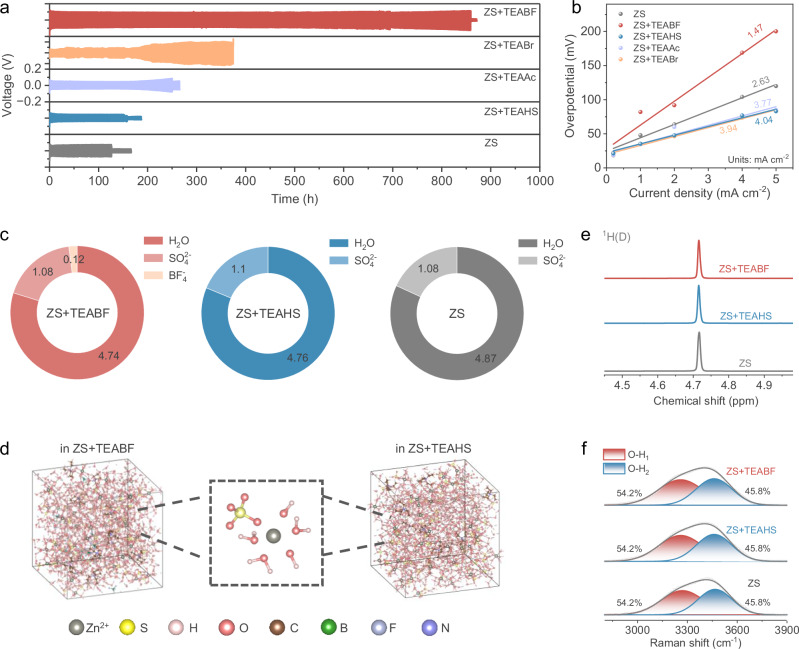
Influence of trace TEA^+^-based additives on Zn^2+^ solvation and electrochemical performance. **a** Galvanostatic cycling performance of Zn||Zn symmetric cells in 10 mA cm^−2^/5 mAh cm^−2^ and **b** exchange current densities of the zinc plating/stripping using different electrolytes. **c** Coordination numbers of H_2_O and anions in the primary solvation shell of Zn^2+^, and **d** corresponding MD snapshots showing electrolyte configurations in the presence of different additives. **e** The normalized ^1^H NMR and **f** fitted Raman spectra of the O-H band obtained from ZS, ZS + TEAHS, and ZS + TEABF electrolytes.

The effect of these additives was evaluated in Zn||Zn symmetric cells under harsh conditions (10 mA cm^−2^/5 mAh cm^−2^). As depicted in Fig. 2a, all additive-containing electrolytes extended Zn plating/stripping stability compared to the blank electrolyte (ZS), which short-circuited after ~126 h. In comparison, the ZS + TEABF electrolyte enabled stable Zn plating/stripping for over 850 h, indicating a strong additive effect. We further examined the variations in the effects of different additives on the kinetics of zinc deposition and the inhibition of side reactions. The scanning electron microscopy (SEM) images indicate that, with the exception of the ZS + TEABF electrolyte, polygonal thin sheets can be detected on the surface of the cycled zinc electrode (indicated by the yellow markings in Supplementary Fig. 1). Based on combined local energy dispersive spectroscopy (EDS) mapping and X-ray diffraction (XRD) analysis of cycled Zn (Supplementary Fig. 2), this structure was identified as basic zinc sulfate (ZSH), a typical by-product formed during the zinc plating/stripping process. The introduction of additives into the electrolyte significantly reduced the formation of ZSH, suggesting that additives effectively mitigate side reactions (Supplementary Figs. 1, 2 and Supplementary Table 1). Moreover, compared to the blank electrolyte, the exchange current density during the zinc plating/stripping process exhibited contrasting trends upon the addition of various additives, indicating that the accompanying anion influences TEA^+^-mediated Zn deposition (Fig. 2b). We further investigated the effects of the additives on the ionic conductivity and pH value of the electrolyte. In addition to the ZS + TEAAc electrolyte, the incorporation of various additives has generally enhanced the ionic conductivity of the electrolyte to some extent, albeit with minor differences among them. This trend is consistent with the results of Zn^2+^ diffusion coefficients derived from MD simulations (Supplementary Figs. 3, 4, Supplementary Table 2 and Supplementary Data 1). In contrast, significant variations in pH were observed, which could be attributed to differences in the degree of anion hydrolysis. Although the pH of the ZS + TEAHS electrolyte was adjusted to match that of the ZS + TEABF electrolyte by adding Zn(OH)_2_, the performance of the zinc negative electrode demonstrated considerable discrepancies (276 h vs. 850 h, Fig. 2a and Supplementary Fig. 5), highlighting that anion identity plays a crucial role even at low additive concentrations (10 mM). It is particularly important to note that in all subsequent tests, the pH of electrolyte was strictly controlled to decouple H^+^ effects from interfacial phenomena.

To determine whether TEA^+^ and/or anion in additives alter Zn^2+^ solvation, we performed MD simulations using ZS, ZS + TEABF, and ZS + TEAHS electrolytes. It should be noted that due to the commercial unavailability and synthetic instability of (TEA)_2_SO_4_, we used TEAHSO_4_ as the blank control group for the TEABF_4_ to explore and highlight the influence of the anions in additives on the regulation of TEA^+^. Radial distribution functions show that Zn^2+^ maintains similar coordination number (CN) with H_2_O and SO_4_^2−^  across all systems (average CN ≈ 4.8 and 1.1, respectively), while BF_4_^−^ coordination was minimal (CN = 0.12) (Fig. 2c, Supplementary Figs. 6, 7, and Supplementary Data 1). As illustrated in Fig. 2d and Supplementary Fig. 8, in all three electrolytes, the Zn^2+^ coordinated with H_2_O and SO_4_^2−^, forming the [Zn(H_2_O)_5_SO_4_^2−^] solvation structure. Spectra analyses, including nuclear magnetic resonance (NMR), Fourier transform infrared spectroscopy (FTIR), further confirmed negligible changes in the hydrogen-bond network and solvation shell upon TEA^+^-based addition (Fig. 2e and Supplementary Fig. 9a, b). This result was further validated through Gaussian fitting analysis of the signals corresponding to the stretching vibrations of the O–H bonds in the Raman spectra within the wavenumber range of 2800–3900 cm^−1^ (Fig. 2f). The relative intensities of the bands associated with strong hydrogen bonds (approximately 3260 cm^−1^, O–H_1_) and weak hydrogen bonds (approximately 3450 cm^−1^, O–H_2_) remained almost unchanged upon the addition of various additives^17^. Furthermore, the band corresponding to Zn^2+^-BF_4_^−^ coordination at 775 cm^−1^ was not observed (Supplementary Fig. 9d)^18^. These collective findings suggest that the significant electrochemical differences stem not from altered solvation structure but might stem from distinct additive behaviors within the EDL.

### Competitive distribution behavior within the Stern layer

**Fig. 3:**
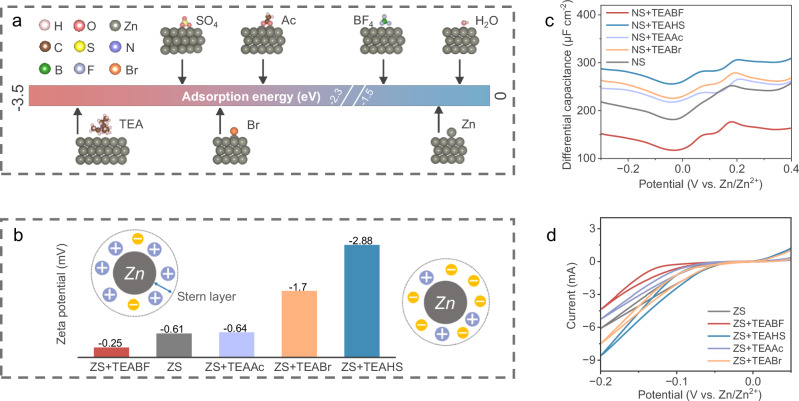
Competitive interfacial arrangement of TEA^+^ and anions. **a** Calculated adsorption energies of various electrolyte species on Zn (002), with insets illustrating their preferred binding configurations. **b** Zeta potentials of Zn surfaces in different electrolytes. The yellow circle and the purple circle in the figure respectively correspond to the anions and cations in the electrolyte. **c** Differential capacitance curves from Na_2_SO_4_-based electrolytes with or without TEA^+^-based salts. **d** Cyclic voltammetry of Zn||Zn symmetric cells conducted from −0.2 to 0.2 V.

The ions and molecules present in the electrolyte serve as key components of the EDL. Owing to their distinct affinities for the electrode surface, these species exhibit varying concentrations within the EDL, thereby significantly influencing the electrochemical behavior. To elucidate the surface adsorption behavior of different additives, we first performed theoretical analyses using density functional theory (DFT) calculations. The results reveal that the neutral TEA exhibits the strongest affinity for the Zn (002) surface among electrolyte species, while the BF_4_ shows the weakest adsorption among the candidate anions (−1.31 eV, Fig. 3a, Supplementary Table 3 and Supplementary Data 2). This suggests the alienation of the BF_4_^−^ ions from the zinc electrode, thereby creating a favorable environment for selective TEA^+^ surface enrichment. Zeta (*ζ*) potential measurements support this: the ZS + TEABF electrolyte yields a near-neutral potential (-0.25 mV), compared to significantly more negative values in the ZS and ZS + TEAHS electrolytes, indicating enhanced TEA^+^ accumulation within the Stern layer (Fig. 3b). By contrast, strongly adsorbing anions such as Br⁻ and Ac⁻ competitively exclude TEA^+^ from the interface, leading to the formation of a more compact and anion-rich Stern layer and undermining the beneficial role of TEA^+^ in Zn regulation. This competitive ion distribution mechanism was further validated by a control experiment in which ZnSO_4_ was partially replaced by Zn(BF_4_)_2_. With increasing proportions of BF_4_^−^, upon addition of 0.01 M TEABF_4_, the *ζ* potential of all systems exhibited a positive shift, and the magnitude of this shift increased progressively with higher BF_4_^−^ content (Supplementary Fig. 10). These results provide direct experimental evidence supporting the proposed interfacial restructuring effect driven by anion selection.

Differential capacitance measurements in Na_2_SO_4_-based electrolytes further corroborate this behavior. Na_2_SO_4_ (NS) was employed as an alternative to ZnSO_4_ to eliminate potential interference from Faraday currents. Among the TEA^+^-containing systems, only the NS + TEABF electrolyte shows a pronounced decrease in differential capacitance, compared to the blank NS electrolyte (Fig. 3c). According to the relationship ${{\mbox{C}}}_{{\mbox{EDL}}}=\varepsilon A/d$, where ${{\mbox{C}}}_{{\mbox{EDL}}}$ is the electric double-layer capacitance, ε is the effective dielectric constant of the interfacial region, ${A}$ is the electrode area, and d is the effective thickness of the EDL, the reduced capacitance suggests an increased effective EDL thickness. This behavior is consistent with the adsorption of larger-volume species at the interface^5^. When considered together with the positive shift in the zeta potential, these results suggest enhanced interfacial accumulation of bulky TEA^+^ cations in the NS + TEABF system. Since excess positive charge near the electrode slows Zn^2+^ nucleation, the ZS + TEABF electrolyte, showing the most TEA^+^ enrichment, which exhibits the highest nucleation overpotential (Fig. 3d). These results confirm that competitive cation/anion assembly within the Stern layer, rather than solvation structure, critically dictates Zn plating/stripping behavior stability.

**Fig. 4:**
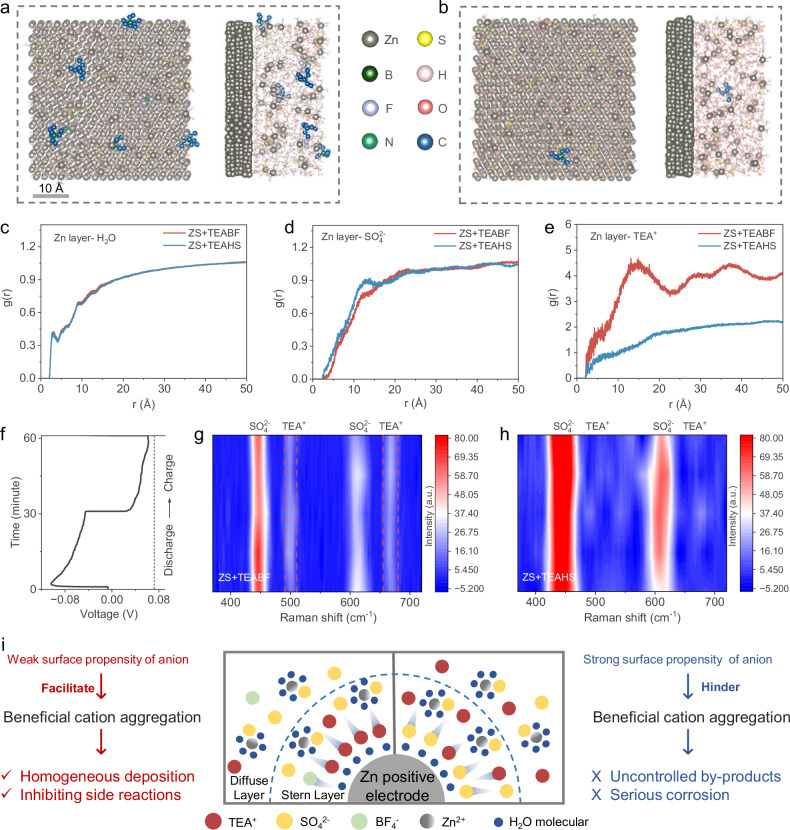
Interfacial chemical environments regulated by anion-dependent adsorption in the Stern layer. MD simulation snapshots showing molecular distributions near the Zn surface in **a** ZS + TEABF and **b** ZS + TEAHS electrolytes. Radial distribution profiles of **c** H_2_O, **d** SO_4_^2−^, and **e** TEA^+^ highlighting differential participation in the EEL. **f** Galvanostatic voltage response during initial Zn plating/stripping at 1 mA cm^−2^/0.5 mAh cm^−2^ under 25 °C, along with in situ Raman spectra tracking TEA^+^ and SO_4_^2−^ evolution at the Zn interface in **g** ZS + TEABF and **h** ZS + TEAHS electrolyte. **i** Schematic illustration of the structural differences in molecular participation at the EEL, induced by competitive behavior between anion and cation.

To validate the proposed role of anion-regulated TEA^+^ distribution in the Stern layer, MD simulations were carried out on the solid/liquid interfaces of the ZS + TEABF and ZS + TEAHS systems. Transverse and cross-sectional snapshots reveal that TEA^+^ can penetrate and distribute more uniformly within the inner layer of the EDL in the ZS + TEABF electrolyte, whereas its presence in the same thickness of the EDL with the ZS + TEAHS electrolyte is minimal (Fig. 4a, b and Supplementary Data 3)^19^. Corresponding density profiles show negligible changes in water distribution, suggesting that trace additives do not substantially modify the H_2_O hydrogen-bond network or solvation structure, contrary to earlier TEA^+^-based studies in Na/Li systems where performance enhancements stem from water suppression via hydrophobic interactions (Fig. 4c)^20,21^. Thus, H_2_O modulation is unlikely to be the origin of improved Zn stability here. Notably, the SO_4_^2−^ ions in the ZS + TEAHS electrolyte exhibit dense accumulation within 10–16 Å of the Zn surface, forming an anion-rich interface that correlates with uneven Zn deposition and by-product formation (Fig. 4d and Supplementary Figs. 1, 2). In addition, it is evident that the introduction of BF_4_^−^, which exhibits low zinc affinity, causes the primary distribution region of SO_4_^2−^ to be located further from the zinc surface in the ZS + TEABF electrolyte. The key divergence lies in the distribution of TEA^+^. A prominent TEA^+^ density peak appears around 14 Å from the Zn surface in the ZS + TEABF electrolyte, corresponding to the Stern layer (Fig. 4e). This peak is absent in the ZS + TEAHS electrolyte, suggesting that highly surface-affinity SO_4_^2−^ does not facilitate the effective aggregation of TEA^+^ accumulation near the surface. BF_4_^−^, in contrast, localizes ~6.7 Å away from the Zn interface (Supplementary Fig. 11), underscoring its lower surface propensity. These results support the hypothesis that TEA^+^ enrichment in the Stern layer, enabled by anion displacement, is critical for guiding interfacial Zn^2+^ deposition. The stark contrast in TEA^+^ localization between the two systems reveals a competitive arrangement mechanism dictated by anion adsorption strength.

To further validate the competitive localization of anions and TEA^+^ in the Stern layer, in situ Raman spectra was employed to probe species distribution on the zinc surface during the initial constant-current charge-discharge process (Fig. 4f). As shown in Fig. 4g, distinct signals near 670 cm^−1^ and 500 cm^−1^ corresponding to TEA^+^ vibrational modes, which are observed only in the ZS + TEABF electrolyte, indicating significant interfacial accumulation of TEA^+^^22^. In contrast, these peaks are absent in the ZS + TEAHS electrolyte, where instead a stronger sulfate signature is detected, reinforcing the notion of an anion-dominated interface that excludes beneficial cations (Fig. 4h). Together, these results establish a clear competitive adsorption mechanism at the Stern layer, where anion surface propensity governs the spatial accessibility of functional cations like TEA^+^. While SO_4_^2−^ hinders interfacial TEA^+^ enrichment, BF_4_^−^, due to its low surface affinity, enables TEA^+^ accumulation that likely modulates Zn^2+^ nucleation energetics and promotes uniform deposition.

Our findings reveal that introducing equal concentrations of TEAHSO_4_ and TEABF_4_ into a baseline ZnSO_4_ electrolyte results in negligible differences in both Zn^2+^ solvation structure and interfacial water orientation, as confirmed by MD simulations and spectra analyses. Despite this similarity in bulk electrolyte structure, the two systems exhibit dramatically different Zn negative electrode stability. By comparing the arrangement of interface ions within the EDL, we attribute the poor performance of ZS + TEAHS system to the high surface affinity of SO_4_^2−^ anions, which preferentially occupy the limited spatial region of the Stern layer, thereby impeding the interfacial enrichment of functional TEA^+^ cation. The resulting cation-deficient interface exacerbates side reactions and promotes uneven Zn deposition, leading to rapid failure of the zinc electrode. In contrast, substitution with BF_4_^−^ (a weakly coordinating, low-affinity anion) relieves competitive spatial constraints within the Stern layer. This anion-released interfacial environment facilitates robust TEA^+^ accumulation near the Zn surface, which in turn modulates interfacial thermodynamics and promotes more uniform Zn nucleation (Fig. 4i).

### Anion-regulated EDL architecture governs organic-inorganic SEI construction

**Fig. 5:**
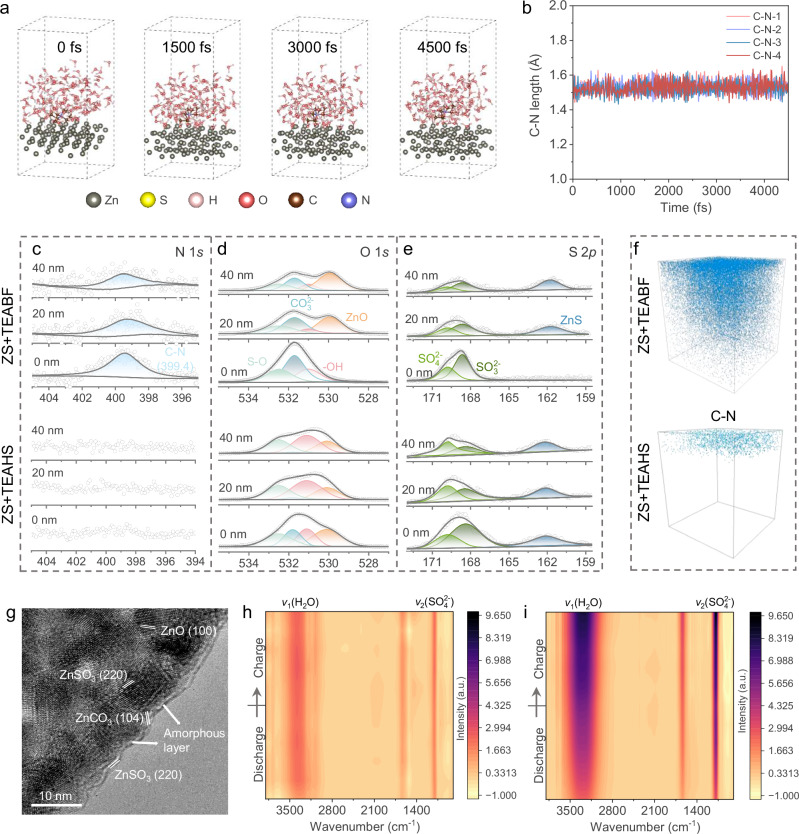
Simulation and characterization of TEA^+^ and SEI components. **a** Snapshots from AIMD simulations depicting TEA existence on the Zn surface. **b** Changes in the length of C-N bond during MD simulation. **c** N 1*s*, **d** O 1*s* and **e** S 2*p* XPS depth profiles of Zn electrodes in the discharging state with 2 mA cm^−2^/1 mAh cm^−2^ after 10 cycles in ZS + TEABF and ZS + TEAHS electrolytes. **f** Three-dimensional view of elemental C-N distributions for cycled Zn in the TOF-SIMS sputtered volumes. After 10 Zn plating/stripping cycles with 2 mA cm^−2^/1 mAh cm^−2^. **g** The HRTEM images after three cycles of zinc plating/stripping on the Cu mesh under 2 mA cm^−2^/1 mAh cm^−2^ in ZS + TEABF electrolyte. The zinc electrodes employed in the aforementioned ex situ tests were all extracted from the coin cells subsequent to the cycling process. In situ FTIR spectra of Zn||Cu cells in the **h** ZS + TEABF and **i** ZS + TEAHS electrolytes at the current density of 1 mA cm^−2^ for 1 mAh cm^−2^ under 25 °C.

The formation of SEI is ascribed to the selective adsorption and electrochemical reduction of diverse components within the EDL, and it assumes a vital role in sustaining the stability of electrode. To clarify the impact of this competitive distribution mechanism on the evolution of the SEI, we conducted detailed investigations into the SEI components formed in the ZS, ZS + TEAHS, and ZS + TEABF electrolytes. Before this, we initially assessed the potential decomposition behavior of TEA^+^ on the electrode surface and its possible contribution to the formation of the SEI. Cyclic voltammetry (CV) tests were initially conducted within a voltage window (0–1.2 V) using deionized water (DIW) and DIW containing 0.01 M TEABF_4_ in a three-electrode system to simulate the actual working voltage environment of the aqueous battery (Supplementary Fig. 12). The results indicated that no other distinct redox signals were detected in the latter system, suggesting that TEABF_4_ exhibits good electrochemical stability in aqueous electrolytes. To further examine the dynamic behavior of TEA^+^ at the zinc electrode, ab initio molecular dynamics (AIMD) simulations were performed, with a focus on the real-time changes in the bond lengths of TEA^+^ in aqueous solutions (Fig. 5a and Supplementary Data 4). The simulation results demonstrated that within 4500 fs simulation time, the C–N, C–C, and C–H bonds remained intact, indicating that TEA^+^ is structurally stable in aqueous electrolytes (Fig. 5b and Supplementary Fig. 13). More significantly, the zinc surface films were collected after cycling for magic-angle spinning (MAS) solid-state NMR detection analysis. The ^13^C spectrum presented only two main signals: the α-C (N^+^–CH_2_) peak at approximately 50.9 ppm and the β-C (–CH_3_) peak at approximately 9.8 ppm^23^, which were consistent with the spectrum of TEABF_4_ powder (Supplementary Fig. 14). This indicates that even after long-term Zn plating/stripping, TEA^+^ can still exist in the SEI in its original structure. Above results underscore that, in contrast to prior studies on analogous ammonium salt additives^24^, TEA^+^ is unlikely to undergo a decomposition reaction akin to the Hoffmann elimination in this study, owing to its high symmetry and the relatively weak acidity of the β-H.

Subsequently, X-ray photoelectron spectroscopy (XPS) was conducted on Zn negative electrode cycled for 10 times in different electrolytes to examine the specific chemical composition of the SEI. In the N 1*s* XPS spectra of zinc electrode cycled in the ZS + TEABF electrolyte, a prominent C–N peak was observed at 399.4 eV, indicating the accumulation of TEA^+^ ions at the electrode-electrolyte interface (Fig. 5c). Moreover, compared to the reference TEABF_4_ powder, this peak exhibited a notable redshift (see Supplementary Fig. 15a), which suggests an enhancement in the local electron density around TEA^+^, likely due to their interaction with the Zn surface^25^. In contrast, no corresponding characteristic peaks were detected for the ZS + TEAHS electrolyte. This provides strong evidence for favorable tendency of TEA^+^ on the Zn surface in the ZS + TEABF electrolyte. Additionally, F 1*s* signals remained negligible during etching, confirming that BF_4_^−^ does not integrate into the SEI, likely due to its weak surface affinity (Supplementary Fig. 15b). O 1 *s* spectra revealed a dominant CO_3_^2−^ signal at the surface in the ZS + TEABF electrolyte, gradually transitioning to ZnO at deeper layers (Fig. 5d). The observed CO_3_^2−^ peak is attributed to CO_2_-derived ZnCO_3_, as supported by C 1*s* spectra across all electrolytes (Supplementary Fig. 16)^26^. In contrast, ZS and ZS + TEAHS samples exhibited SEIs rich in ZnO, Zn(OH)_2_, and sulfate product, consistent with intensified interfacial corrosion in the absence of TEA^+^ accumulation (Fig. 5d, e and Supplementary Fig. 17a–c). Furthermore, it was observed that in both the ZS and ZS + TEAHS electrolytes, the content of Zn^0^ increased with greater etching depth, suggesting incomplete coverage by the SEI (Supplementary Fig. 17d–f). This non-uniform SEI coverage may result in continuous electrolyte corrosion of the fresh zinc metal, which could hinder the long-term stability and performance of the Zn negative electrode.

Time-of-flight secondary ion mass spectrometry (TOF-SIMS) provided 3D mapping of interfacial TEA^+^. A clear C–N signal gradient was detected in the ZS + TEABF electrolyte, indicative of TEA^+^-enriched SEI (Fig. 5f). However, this signal was nearly absent in the ZS + TEAHS electrolyte, suggesting negligible cation adsorption. High-resolution transmission electron microscopy (HRTEM) further corroborated these results: the SEI derived from ZS + TEABF electrolyte displayed defined lattice fringes of ZnO, ZnCO_3_, and ZnSO_3_, along with surface-adhered amorphous regions consistent with organic species (Fig. 5g)^27,28^. In contrast, SEI from ZS + TEAHS electrolyte was dominated by crystalline (Zn(OH)_2_)_3_(ZnSO_4_)(H_2_O)_3_ (ZSH), indicative of side reaction by-products (Supplementary Fig. 18). To monitor interfacial species evolution in real time, the in situ FTIR spectra presented in Fig. 5h, i illustrate the variation of absolute transmittance over time. These spectra depict the evolution of the O-H stretching vibration of interfacial H_2_O and the S-O stretching vibration of SO_4_^2−^ in various electrolytes. The gradual increase in the absolute transmittance of SO_4_^2−^ and H_2_O in the ZS + TEAHS electrolyte signifies a more pronounced consumption rate, indicating that severe side reactions occur at the interface, resulting in a higher content of ZSH components in the SEI. Additionally, the characteristic peaks of SO_4_^2−^ and H_2_O can serve as indicators for the concentration changes of Zn^2+^^29^. The stable peak strength observed in the ZS + TEABF electrolyte suggest a uniform Zn^2+^ flux within the EDL, effectively inhibiting dendrite formation. Based on the above results, it becomes evident that in the ZS + TEABF electrolyte, the addition of surface-avoiding anions allows for ample distribution of TEA^+^ within the Stern layer and its active involvement in the SEI formation process. Ultimately resulting in a composite SEI with an inorganic-organic co-mosaic outer layer and an inner layer rich in inorganic content. In contrast, due to inadequate optimization of EDL structure by TEA^+^ in the ZS + TEAHS electrolyte, increased sulfate-induced parasitic reactions at the surface lead to the formation of an incomplete SEI lacking organic components.

### Electrochemistry kinetics governed by SEI architecture

**Fig. 6:**
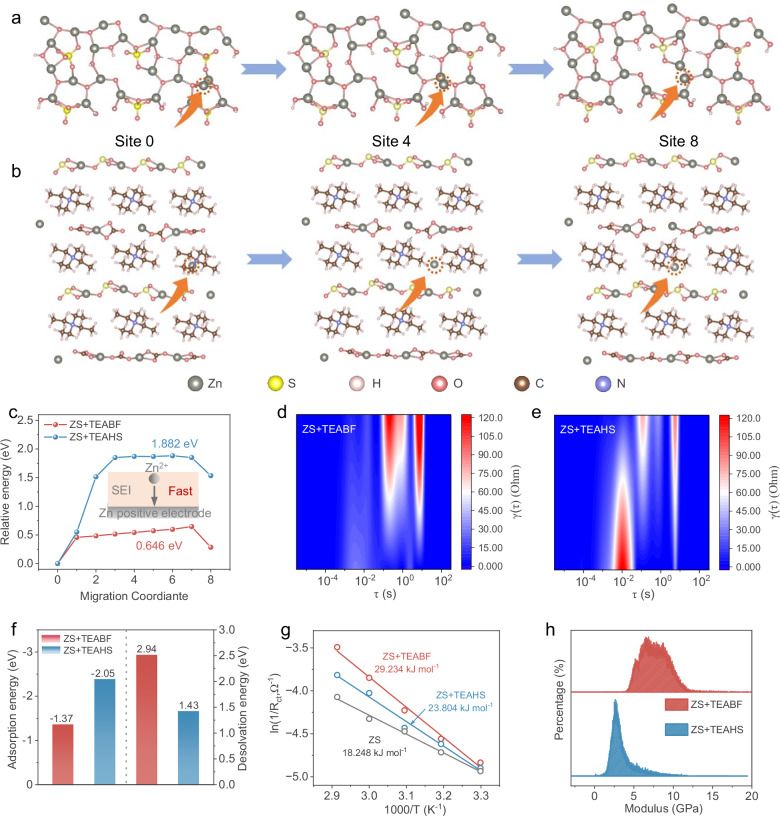
Mechanical robustness and Zn^2+^ transport properties of SEI. Representative Zn^2+^ migration pathways through SEIs derived from **a** ZS + TEAHS and **b** ZS + TEABF electrolytes, constructed based on experimental HRTEM and XPS data. **c** Corresponding Zn^2+^ migration energy barriers in the two SEIs. In situ DRT spectra for Zn||Zn symmetric cells after 1 and 5 cycles of Zn plating/stripping at a current density of 2 mA cm^−2^ and capacity density of 1 mAh cm^−2^ in **d** ZS + TEAHS and **e** ZS + TEABF electrolyte under 25 °C. **f** The calculated adsorption energy and desolvation energy of hydrated Zn^2+^ on SEIs and **g** activation energy fitted by the Arrhenius formula in different electrolytes. **h** AFM-derived modulus distributions of the outer SEI layers. The Zn negative electrode employed in this test was prepared subsequent to undergoing 50 zinc deposition/stripping at current density of 2 mA cm^−2^ for 1 mAh cm^−2^, and was in a charged state at that juncture.

As is well known, the SEI plays a pivotal role in regulating ion desolvation, interfacial charge transfer, and the reversibility of metal plating/stripping processes. Yet, a mechanistic understanding of how additive-induced interfacial regulation modulates SEI evolution and Zn negative electrode performance remains limited. To bridge this gap, we constructed two simplified SEI models derived from experimental characterizations (HRTEM and XPS, Supplementary Figs. 19, 20): one formed in ZS + TEAHS electrolyte containing primarily ZnO and ZSH, and another formed in ZS + TEABF electrolyte composed of alternating layers of ZnSO_3_-TEA-ZnCO_3_-TEA-ZnSO_3_. DFT calculations reveal that the energy barrier for Zn^2+^ diffusion through the organic-inorganic hybrid SEI derived from ZS + TEABF electrolyte (0.646 eV from site 0 to site 8) is significantly lower than that in the SEI from ZS + TEAHS electrolyte (1.882 eV), underscoring the critical role of organic components in facilitating Zn^2+^ mobility (Fig. 6a-c, and Supplementary Data 5).

To further resolve the kinetics at the Zn-electrolyte interface, we conducted in situ DRT analysis to decouple the electrochemical contributions from SEI transport, interfacial migration, charge transfer, and ion diffusion. As shown in Fig. 6d, e, with increasing cycle numbers, the ZS + TEABF system exhibits a gradual decrease in resistance associated within SEI diffusion (*R*_*SEI*_), indicative of a maturing, conducting-ion SEI layer. However, the relatively high concentration of hydrophobic TEA^+^ in the Stern layer impedes the diffusion and desolvation of hydrated Zn^2+^, leading to the highest charge transfer resistance in the ZS + TEABF electrolyte. Given that the resistance associated with *R*_*SEI*_ is smaller compared to other types, it predominantly manifests as charge transfer and diffusion resistance. Consequently, the activation energy (*E*_*a*_) required for zinc ion deposition in the ZS + TEABF electrolyte is the highest, which aligns well with the results obtained by fitting electrochemical impedance spectroscopy (EIS) at different temperatures and simulated in theoretical calculation models (Fig. 6f, g, Supplementary Fig. 21 and Supplementary Table 4). Compared to the ZS + TEAHS electrolyte, the SEI derived from the ZS + TEABF electrolyte exhibits a smoother desolvation and charge transfer process, which is conducive to forming smaller zinc nucleation grains and achieves uniform deposition. This conclusion was corroborated by the measurement of the surface roughness of zinc electrodes after cycling using atomic force microscopy (AFM). The surface height difference of the zinc negative electrode in the ZS + TEABF electrolyte was approximately 250.5 nm, significantly lower than that of 810.8 nm observed in the ZS + TEAHS electrolyte (Supplementary Fig. 22), indicating that the milder deposition kinetics induced by TEA^+^ contribute to the formation of a more uniform and compact zinc deposition morphology.

Additionally, AFM was employed to examine the mechanical strength of the SEI layers formed in different electrolytes. As shown in Supplementary Fig. 23 and Fig. 6h, the SEI generated in the ZS + TEABF electrolyte exhibits significantly higher mechanical modulus values (~5.8–9.3 GPa) compared to that in the ZS + TEAHS system (~2.2–3.3 GPa). This enhancement arises from the intimate integration of organic and inorganic species, which yields a denser, more resilient interphase. Complementary DFT calculations further reveal that the key SEI constituents in the ZS + TEABF system possess lower binding energies (Supplementary Fig. 24 and Supplementary Data 5), indicative of improved thermodynamic stability and greater resistance to mechanical degradation during cycling^30^.

Together, these results highlight the critical role of Stern layer regulation in governing the uniformity, composition, and robustness of the SEI. Such variations in SEI architecture profoundly influence Zn deposition behavior by modulating interfacial ion flux and mechanical confinement. Notably, although the TEA^+^-rich interphase promotes accelerated Zn^2+^ transport within the SEI, the initial adsorption and desolvation of solvated Zn^2+^ at the electrolyte-SEI interface is kinetically hindered—acting as a rate-determining step in the deposition process. We thus propose that this modest interfacial kinetic barrier serves a beneficial function, suppressing uncontrolled nucleation and guiding well-oriented Zn growth, ultimately contributing to improved cycling stability.

### Electrochemical performance and practical implications of anion-released interface engineering

To validate the efficacy of trace non-solvating additives, we comprehensively investigated their impact on Zn negative electrode stability and SEI formation through anion-released interfacial regulation. Prior to this, the optimal addition concentration of TEABF_4_ was determined. Under the conditions of 10 mA cm^−2^/5 mAh cm^−2^, the ZS + TEABF-0.01 electrolyte enabled the zinc electrode to cycle stably for over 850 h, significantly outperforming other tested concentrations (0.005, 0.03, and 0.1 M, see Supplementary Fig. 26). Further analysis and comparison of the interfacial dynamics were carried out using exchange current density test and DRT (Supplementary Fig. 27).

**Fig. 7:**
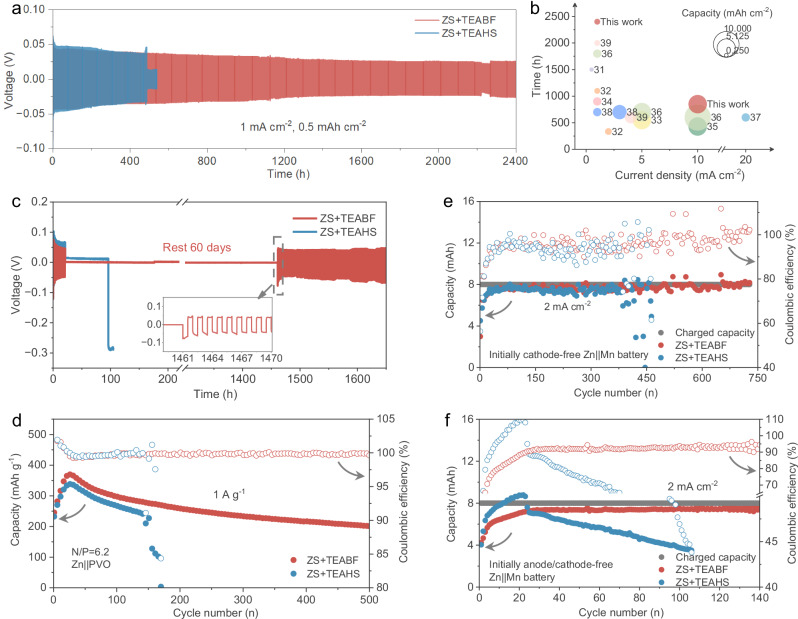
Effects of anion-released interface engineering on Zn anode stability and full-cell performance. **a** Long-term galvanostatic cycling of Zn||Zn symmetric cells in ZS + TEABF and ZS + TEAHS electrolytes at 1 mA cm^−2^/0.5 mAh cm^−2^. **b** Comparison of cycling life, current density, and areal capacity per cycle with state-of-the-art trace additive strategies^31–39^. The source of the-literature data shown in this figure can be found in Supplementary Information, Table 4. **c** Static resting test of Zn||Zn cells over 60 days. **d** Cycling performance of Zn ||PVO full cells at 1 A g^−1^ with a practical *N*/*P* ratio of ~6.2. **e** Cycling of Mn-based initially positive electrode-free Zn||Mn cells at 2 mA cm^−2^. **f** Initially dual-electrode-free Zn||Mn cells with 8 mAh charging capacity and 2 mA cm^−2^ discharge current density, operated in the ZS + TEABF and ZS + TEAHS electrolytes.

Compared to the ZS + TEABF-0.01 electrolyte, the *τ* value associated with zinc ion diffusion in the ZS + TEABF-0.1 electrolyte was found to be higher after multiple charge-discharge cycles. This observation may be attributed to the increased concentration of TEA^+^ ions, which potentially enhanced the thickness of the EDL and the SEI, thereby elongating the diffusion pathway of Zn^2+^ ions and raising the migration energy barrier. Consequently, the optimal additive concentration of TEABF_4_ was determined to be 0.01 M. Figure 7a illustrates that in the ZS + TEABF electrolyte, the symmetric battery exhibits stable operation over a period of 2400 h under 1 mA cm^−2^/0.5 mAh cm^−2^ condition, indicating the effectiveness of anion-released Stern layer in stabilizing zinc deposition during long-term Zn plating/stripping. In contrast, the battery in ZS + TEAHS electrolyte experiences short-circuiting within 500 h due to the accumulation of rigid zinc dendrites. Compared with recently published studies on trace electrolyte additives, this work demonstrates certain competitiveness in cycle life and tolerable current density (Fig. 7b and Supplementary Table 5)^31–39^. Furthermore, the Zn||Cu asymmetric cells and the symmetric cells demonstrated more reversible plating/stripping (average reversible CE = 99.89%) and long-term stability under static condition (>60 days) in the ZS + TEABF electrolyte, which could be attributed to the effective suppression of side reactions (Fig. 7c and Supplementary Figs. 28, 29).

Moreover, the full cells using poly (3,4-ethoxylene dioxy thiophene) (PEDOT)-V_2_O_5_ (PVO) positive electrode are assembled in the ZS + TEAHS and ZS + TEABF electrolytes to evaluate practical value. The incorporation and internal polymerization of EDOT can reduce the crystallinity of V_2_O_5_, thus mitigating severe structural phase transformations induced by Zn^2+^ insertion/extraction during cycling (Supplementary Figs. 30, 31). Furthermore, this modification introduces additional active sites, facilitating faster zinc ion transport kinetics (Supplementary Fig. 32)^40^. To meet the practical application, the battery with a low Negative/Positive capacity (*N*/*P*) ratio (~6.2) underwent an extended cycle test at a low specific current of 1 A g^−1^. The results reveal that after 500 cycles, the capacity retention rate of the Zn||PVO full cell in the ZS + TEABF electrolyte is approximately 81.9%, while another cell in the ZS + TEAHS electrolyte fails after only 160 cycles (Fig. 7d). When the specific current is increased to 10 A g^−1^, the full cell in the ZS + TEABF electrolyte achieved a maximum capacity retention rate of 67.7% in 1500 cycles, much higher than the cell in the ZS + TEAHS electrolyte (Supplementary Fig. 33). We further conducted a more rigorous evaluation of the electrolyte’s performance by fabricating a pouch cell with titanium pre-deposited zinc, along with PVO positive electrode (*N*/*P* = 2.51) (Supplementary Fig. 34). Specifically, the TEABF_4_-based system maintained stable cycling over 120 cycles with consistently CE approaching 100%. In contrast, the TEAHS-based pouch cell experienced severe capacity decay (retention: 39.2%) after only 36 cycles. These results highlight the dual benefit of TEABF_4_ in suppressing Zn negative electrode corrosion and limiting PVO positive electrode dissolution (Supplementary Figs. 35, 36, and Supplementary Table 6).

To further probe the additive’s universality, we constructed an initially positive electrode-free electrolytic Zn||Mn cell in ZS + TEABF electrolyte to evaluate the expandability of Zn negative electrode protected by anion-released interface engineering (Supplementary Fig. 37). Under a current density of 2 mA cm^−2^, the cell in ZS + TEABF electrolyte exhibits stable cycling for 730 cycles with a capacity of 2 mAh cm^−2^. In contrast, active zinc loss is pronounced after only 380 cycles due to the accumulation of side reactions at the negative electrode surface in the ZS + TEAHS electrolyte, resulting in sharp fluctuations in CE (Fig. 7e). Finally, we designed a more challenging initially dual-electrode-free electrolytic Zn||Mn battery. As depicted in Fig. 7f, the battery in the ZS + TEABF electrolyte exhibits specific energy of 896.9 Wh kg^−1^ at an 8 mAh-scale capacity over 140 cycles based on Mn^2+^/MnO_2_ redox, accompanied by a high average discharge platform voltage of 1.45 V (Supplementary Fig. 38). However, when operated with the ZS + TEAHS electrolyte, the CE initially exceeds 100% due to vigorous side reactions and failed prematurely after 25 cycles. Collectively, these findings establish that surface-anion-released interfacial engineering via trace TEABF_4_ additives not only enables highly reversible Zn plating/stripping but also stabilizes full-cell operation under practical constraints, offering a promising avenue for advanced aqueous Zn-based energy storage.

## Discussion

In this work, we unveil an anion-released interfacial engineering strategy that fundamentally redefines the role of trace non-solvating additives in regulating electrolyte–electrode interactions. In situ spectra analysis revealed a competitive spatial arrangement between anions and cations within the Stern layer, which directly governs their accessibility to the electrode surface and subsequently modulates interfacial electrochemical processes. By replacing high-affinity anions with surface-avoiding BF_4_^−^, we successfully enriched the distribution of TEA^+^ within the Stern layer, enabling its active incorporation into the SEI. This structural rearrangement not only suppressed parasitic reactions but also yielded a composite SEI with rapid ion transport characteristics. Through systematic decoupling of SEI function and electrochemical kinetics, we demonstrate that a coupling of slower interfacial desolvation and faster Zn^2+^ transport across the SEI is essential to achieving dendrite-free, highly reversible Zn plating under demanding conditions.

The resultant zinc negative electrodes exhibited robust cycling stability across symmetric and full-cell configurations, including under practical current densities and high areal capacities. This study highlights the critical influence of ion-specific interfacial arrangements beyond classical solvation models and provides a generalizable framework for electrolyte design in aqueous batteries. Our findings offer mechanistic insights into electrolyte–interface coupling and we hope they will open avenues for constructing robust electrode–electrolyte interfaces through selective ion displacement.

## Methods

### Preparation of ZS electrolyte and additive electrolyte

143.78 g ZnSO_4_·7H_2_O (99.5%, Innochem) was dissolved in deionized water to form 250 mL homogeneous solution for the preparation of 2 M ZnSO_4_ electrolyte, which was denoted as ZS electrolyte. Then, different moles of tetraethyl ammonium tetrafluoroborate (TEABF_4_, 99%, Innochem) powder were dissolved in 2 M ZnSO_4_ electrolyte respectively to prepare hybrid electrolytes containing different concentrations (0.005, 0.01, 0.03 and 0.1 M) TEABF_4_ (denoted as ZS + TEABF electrolyte). The electrolytes with different additives were prepared by introducing TEAHSO_4_ (99%, Aladdin), TEABr (99%, Innochem) and TEAAc (99%, Adamas-beta) in the same method. Commercially available TEAHSO_4_ (99%, Apinno) was selected because battery-grade (TEA)_2_SO_4_ is neither available nor reliably synthesized; repeated laboratory attempts produced hygroscopic solids with residual acid. In aqueous solution TEAHSO_4_ is easily deprotonated, thereby delivering the same TEA^+^/SO_4_^2−^ pair required for this study.

### Preparation of the carbon cloth@PEDOT-V_2_O_5_ positive electrode

The synthesis of the PEDOT-V_2_O_5_ (PVO) follows the method described in our previous work^41^. A solution was prepared by dissolving 1.5 mL of 3,4-ethoxylene dioxy thiophene (EDOT, 98%, Innochem) monomer and 3 g of commercial V_2_O_5_ (99.6%, Alfa Aesar) in 100 mL of 2 M NaCl solution, which was then magnetically stirred for a period of 5 days. The impurities were subsequently washed away using deionized water and alcohol, resulting in the formation of a gray-green product that was obtained through centrifugation. Finally, the PVO powder was obtained after freeze-drying.

To prepare the paste for coating, 80 wt% PVO, along with 10 wt% polyvinylidene fluoride (PVDF, dodochem) and 10 wt% carbon nanotube (CNT) conductor were dissolved uniformly in N-Methyl-2-pyrrolidinone (NMP, 99.9%, Innochem) and subjected to magnetic stirring for a duration of 6 h. This paste was then evenly coated on one side of the carbon cloth and dried under vacuum conditions at 60 °C for 12 h to obtain the positive electrode consisting of carbon cloth@PVO composite material. The mass loading of PVO on the carbon cloth is estimated to be around 3.6 mg cm^−2^.

### Battery assembly

Zn||Zn symmetric coin cell (CR2032) was assembled by zinc foils (99.99%, Qingyuan Metal Co., Ltd.) measuring 15 mm in diameter and 50 μm in thickness, along with a glass microfiber filter (Whatman, GF/D, with a thickness of 675 μm and a pore size of 2.7 μm) measuring 16 mm in diameter. Prior to using the zinc foil, the surface oxide layer should be polished off with sandpaper, followed by cleaning with absolute ethanol. The preparation process of the Zn||Cu asymmetric cell and the Zn||PVO full cell is identical to that of the symmetric cell, except for substituting a 15 mm diameter copper foil (99.99%, Qingyuan Metal Co., Ltd.) and a 12 mm diameter carbon cloth@PVO in place of the zinc foil on the positive side. Specifically, the thickness of the zinc foil used in the Zn||PVO battery with low *N*/*P* is 20 μm. ZS + TEABF, and ZS + TEAHS were applied as the electrolytes, respectively. The amount of electrolyte injected was kept at 120 μL.

The low *N*/*P* ratio PVO single-layer pouch cell consists of a titanium foil pre-deposited with zinc (~ 2.22 mAh cm^−2^) and a PVO positive electrode. The area of each electrode measures 4.5 × 3 cm, and the separator was cut into a 5.5 × 4 cm rectangle and positioned in the middle between the two electrodes. The active material loading of PVO positive electrode is approximately 1.5 mg cm^−2^, resulting in an *N*/*P* ratio of 2.51. The quantity of electrolyte added was 1.5 mL. During the sealing procedure, an exhaust hole is reserved to expel the surplus electrolyte. The pre-deposited zinc titanium foil is prepared by placing a 50 μm zinc foil (serving as the negative electrode) and a 30 μm titanium foil (99.99%, Qingyuan Metal Co., Ltd., serving as positive electrode current collector) in a 100 mL beaker filled with 80 mL of 2 M ZnSO_4_ electrolyte. The immersion area of the electrodes is 3 × 4.5 cm. The device is subjected to a constant-current discharge at 30 mA for 1 h. After the electrodes are removed, they are rinsed with deionized water and ethanol and then dried, which can be utilized for the subsequent assembly of pouch cells. All the SEI characterization samples in this work have undergone at least 5 cycles of electrochemical cycling. Nevertheless, the pre-deposited zinc step consisted of a single 1 h constant-current deposition. Consequently, we hypothesize the stability of the pre-deposited zinc negative electrode was primarily determined by the SEI film originating from the electrolyte during the cycling process in pouch cells.

The assembly process of the Zn||Mn beaker battery involves adding 0.3 M MnSO_4_·H_2_O (AR, Sinopharm) to the electrolyte solution, placing an area of 2 × 2 cm and thickness of 50 μm Zn foil or 20 μm Cu foil on the positive electrode, and using an area of 2 × 2 cm carbon felt (Tianjin Carbon Factory) on the negative electrode. This setup realizes the dissolution/deposition reaction of Mn^2+^/MnO_2_ on the surface of the carbon felt through charging and discharging. All the current collectors were not subjected to any additional treatments prior to use. The beaker batteries tests were carried out in 25 mL beakers filled with 20 mL of electrolyte solution and all the cells were tested within a constant-temperature chamber maintained at 30 °C in air environment unless otherwise stated.

### Material characterizations

The morphology and imaging of the materials were investigated using a scanning electron microscopy (SEM, SU8020, at 5 kV) equipped with energy dispersive spectroscopy (EDS, at 10 kV). HRTEM was performed using a JEOL JSM-2010 microscope and analyzed by DigitalMicrograph, the voltage is set to 200 kV. The elemental distribution on the cycled electrode surface was analyzed utilizing time-of-flight secondary ion mass spectrometry accessories (TOF-SIMS Accessories, TESCAN LYRA3 GM, TESCAN, Czech). Throughout the test, a 30 keV Ga^+^ beam with 200 pA of ion current was employed to bombard the electrode surface. The crystal structures of all samples were characterized by X-ray diffraction analysis (XRD, Rigaku Smart Lab, Cu-Kα, *λ* = 0.15406 nm, 40 kV, the step size is 0.026 degrees.). X-ray photoelectron spectroscopy (XPS) data was collected using a Thermo Fisher 250Xi electron spectrometer that was equipped with a monochromatic Al-Kα radiation source (1486.6 eV) and had a beam size of 650 μm. The acquired XPS data were calibrated against the C 1*s* at 284.8 eV. Atomic force microscopy (AFM, Dimension Icon, Bruker, Germany) equipped with an RTESPA-525 probe was employed to evaluate the surface height variations of the zinc negative electrode and the Young’s modulus of the SEI in the air atmosphere. The aforementioned test electrodes were all obtained from intact 2032-type coin cells that had undergone electrochemical cycling.

The pH value was determined employing a pH meter (PUCHUN, PHS-3C). All ^13^C solid-state nuclear magnetic resonance (ssNMR) characterization was conducted on a 500 MHz JEOL JNM-ECZ500R spectrometer equipped with a PhoenixNMR 3.2 mm MAS probe, which operates at 50 MHz. With a magic angle rotation rate of 10 kHz, the probe tilt angle was adjusted, and the magic angle calibration was executed using the adamantane standard sample. The resonance frequency was 125.77 MHz, the relaxation delay was 3 s, the acquisition time was 0.0162 s, and the number of scans for the SEI sample and TEABF_4_ were 436 and 521, respectively. The raw data is subjected to manual phase correction and subsequently normalized prior to being utilized for plotting. The SEI samples were prepared by scraping the surface film of the cycled zinc electrode using a scalpel, and the mass of the samples was approximately 20 mg.

### Electrochemical tests

The LAND battery test system was utilized for conducting constant current/voltage charge and discharge tests on cells. All the battery performance test data were based on measurements carried out on at least three sets of independently assembled parallel battery samples. The cyclic voltammetry curves and differential charge-voltage curves of cells were measured using CHI660E electrochemical workstation. The differential capacitance curve is calculated according to Eq. (1):

1 $${{\rm{C}}}=-{(\omega Z{im})}^{-1}$$

where C represents the differential capacitance, *ω* denotes the angular frequency, and *Z*_*im*_ corresponds to the imaginary component of impedance. The specific frequency employed in this calculation is set at 1000 Hz. It should be noted that all measurements were conducted under strictly controlled and identical pH conditions to exclude possible pH-induced contributions to the capacitance response (specific operational details are provided in the following section).

Depth-of-discharge (DOD) is determined by the Eq. (2):

2 $${{\rm{DOD}}}=(Iat)/{Cm}\times 100\%$$

where *I*_*a*_ represents the current during deposition/stripping, *t* represents the time of deposition or stripping, *C* is the theoretical capacity of zinc metal (820 mAh g^−1^), and m represents the quality of zinc foils.

After being allowed to stabilize for 1 h under open-circuit conditions, EIS measurements were carried out on the assembled coin cells using CHI660E electrochemical workstation. A 5 mV potentiostatic perturbation was applied during the test, with a frequency scanning range of 0.01–100,000 Hz. A total of 73 test frequency points was collected. The EIS tests at different temperatures were performed by placing the coil-symmetric cells in a temperature-controlled oven. The obtained EIS data were analyzed using ‘DRT tools’.

### Electrolyte pH standardization

Given the substantial influence of pH on the differential capacitance at the electrode surface, during the differential capacitance test, the pH of each electrolyte was strictly and uniformly regulated. At 25 °C, the initial pH values of the original NS, NS + TEABF, NS + TEAHS, NS + TEABr, and NS + TEAAc electrolytes were 5.56, 5.90, 3.03, 5.60, and 6.55, respectively. The slightly sub-neutral pH of the NS electrolyte (5.56) is attributable to the absorption of atmospheric CO_2_, which dissolves in water to form carbonic acid, lowering the equilibrium pH to approximately 5.6 in unbuffered aqueous solutions^42,43^. Notably, the presence of dissolved CO_2_ at this level has been reported to facilitate the formation of a favorable SEI on the electrode surface, and its consistent contribution across all electrolyte groups in this study has been verified (see Supplementary Fig. 16)^26^. Since one of the intrinsic advantages of aqueous batteries is that electrolytes can be prepared and handled under ambient conditions, the presence of dissolved CO_2_ is both inevitable and representative of practical operation. To achieve pH-matched conditions for comparative testing, 20 mL of each electrolyte was taken for adjustment: 30 μL of 0.025 M H_2_SO_4_ was added to NS + TEABF to adjust the pH to 5.52; 150 μL of 1.5 M NaOH and 30 μL of 0.1 M NaOH were added to NS + TEAHS to stabilize the pH at 5.62; and 60 μL of 0.1 M H_2_SO_4_ was added to NS+TEAAc, resulting in a final pH of 5.57.

To directly probe the influence of BF_4_^−^ on the adsorption behavior of the TEA^+^ cation, the ZS + TEAHS was selected as a reference electrolyte. However, the intrinsic acidity of HSO^−^_4_ (pKa ≈ 1.99) resulted in a lower pH in the ZS + TEAHS electrolyte (2.27), which induced severe corrosion of the zinc negative electrode and precluded a fair electrochemical comparison. To mitigate this, 20 mg of Zn(OH)_2_ (99%, Tianjin Damao) was employed to alkalize 20 mL of the ZS + TEAHS electrolyte. Once the Zn(OH)_2_ solid was completely dissolved (the added Zn^2+^ concentration in the system was less than 0.5%, which would not significantly interfere with the electrochemical behavior of Zn^2+^), the supernatant was collected. Subsequently, by adding pure ZS + TEAHS electrolyte, the pH of the system was accurately adjusted to a level comparable to that of the ZS + TEABF electrolyte (approximately 2.7) for subsequent comparative tests.

### Details of in situ FTIR experiments

In situ FTIR measurements were conducted at room temperature using a Thermo Fisher Nicolet 6700 spectrometer. An in situ electrochemical cell was constructed with zinc foil as the working electrode and copper mesh as the counter electrode. The electrodes were positioned on a ZnSe prism, with the incident angle set to 60°, spectral resolution adjusted to 4 cm^−1^, and the results presented as transmittance spectra. The testing protocol involved charging and discharging the battery at a constant current density of 1 mA cm^−2^ for 1 h each.

### Details of in situ Raman experiments

In situ Raman test was performed using the Beijing Zhongyan Huanke RX50M confocal Raman spectrometer. The experimental setup included a custom-designed electrochemical Raman cell, with a laser wavelength of 533.8 nm, a power intensity set at 10%, and an integration time of 10 s. The spectrometer employed the 23-G600-B500 grating, centered at a wavenumber of 2000 cm^−1^. Both the working electrode and the counter electrode were zinc foil. The testing conditions involved constant current charging and discharging at a current density of 1 mA cm^−2^ for 0.5 h each.

### DFT calculations

The adsorption energy of different substances on the zinc surface was carried out using the Quantum ESPRESSO software package^44^. The electron-ion interactions in the system were accurately described by the all-electron projector augmented wave (PAW) pseudopotential, while the exchange-correlation interaction was modeled using the Perdew-Burke-Ernzerhof (PBE) functional within the generalized gradient approximation (GGA) framework^45^. The DFT-D3 method was employed to account for van der Waals interactions between molecules and substrates^46^. A plane-wave basis set with a cutoff energy of 50 Ry was utilized, and the corresponding charge density cutoff energy was set to 400 Ry. The Zn (002) crystal plane was chosen as the substrate for molecular adsorption because both theoretical and experimental studies have confirmed that preferential epitaxial growth of the (002) crystal plane occurs during the zinc deposition process. And all surface-adsorption models were performed using a charge-neutral cluster or molecule. To eliminate spurious interactions between periodic mirror image structures, a vacuum layer of 15 Å was introduced in the direction perpendicular to the surface during modeling. Given the size variations among the molecules, different-sized Zn (002) supercells were selected as adsorption substrates to ensure consistent intermolecular distances or near-uniform coverage under periodic boundary conditions. Specifically, TEA required a 6 × 6 Zn (002) supercell due to its largest size, whereas BF_4_, SO_4_, and Ac used a 5 × 5 Zn (002) supercell given their comparable sizes. For the smallest species, H_2_O, Br, and Zn, a 4 × 4 Zn (002) supercell was employed. This ensured an intermolecular distance of approximately 10 Å across all systems. During the calculations, the Monkhorst-Pack scheme was adopted for k-point sampling. System convergence tests determined that a k-point grid density of 0.03 × 2π/Å was sufficient for self-consistent field (SCF) calculations and lattice optimizations. For non-self-consistent field (NSCF) calculations, such as energy evaluations, a denser k-point grid density of 0.02 × 2π/Å was used to enhance the accuracy of Brillouin zone integrations. Structural relaxations were performed accordingly.

The adsorption energy E_ads_ can be defined as, E_ads_ = E_*M_-E_*_-*E*_*M*_, where E_*_ and E_*M_ denote the total energy of the system prior to and following adsorption, respectively, while *E*_*M*_ represents the energy of a M molecule under vacuum conditions.

### MD simulations

Force-field molecular dynamics (FFMD) simulations were perform using the MD software Gromacs^47,48^. The forced field parameters were obtained from Amber fields^49^. The size of ZS + TEAHS box is 55.9 × 55.6 × 69.2 Å, which contains 3900 H_2_O molecules, 10 TEAHSO_4_ molecules, 200 Zn^2+^ ions and 200 SO^2−^_4 _ions. The size of ZS + TEABF box is 55.9 × 55.6 × 69.4 Å, which contains 3900 H_2_O molecules, 10 TEABF_4_ molecules, 200 Zn^2+^ ions and 200 SO^2−^_4 _ions. The periodic boundary conditions were set in all three directions. The electrostatic interactions were computed using Ewald methods^50^. The atomic charges were calculated at the B3LYP-D3/def2-SVP theoretical level by determining the RESP charges. Additional force field parameters were generated using dedicated software packages, such as Sobtop. The real space electrostatic and non-electrostatic interactions were handled with a cutoff length of 15.5 Å, and the integration step size was set at 1 fs. The system was initially heated from 200 K to 500 K via a 0.5 ns temperature increase and annealing process. Subsequently, the simulation duration was prolonged by 0.5 ns to enable the system to fully relax and attain equilibrium, with the aim of identifying the most optimized structural model to serve as the initial configuration for the subsequent particles, volume, and temperature (NVT) ensemble simulation. The temperature was performed in Nose method. Finally, a 0.5 ns production simulation was run for post-processing analysis. The radial distribution function (RDF) and Mean Square Displacement were obtained by Forcite analysis.

### AIMD simulations

AIMD simulations were performed on the surface of a zinc crystal substrate in an aqueous solvent containing TEABF_4_ salt by utilizing the CASTEP module^51^. A periodic slab model (18.5 × 16.0 Å) was established, which incorporated the zinc crystal surface ((002) plane, 3 layers) in contact with an explicit aqueous solution (120 H_2_O molecules, the density was set to ~1.0 g/cm^3^) containing the target organic molecule. The total height of the simulation cell was approximately 32 Å, with a 15 Å vacuum layer above the liquid surface to mitigate spurious dipole interactions. Initially, the system geometry was optimized with an energy convergence criterion of 1 × 10^−5 ^eV/atom and a maximum force tolerance of 0.0001 Å. Subsequently, MD simulations were conducted in the NVT ensemble for a total duration of 4500 fs, employing the Nosé thermostat to maintain the system (simulations were performed at 298 K)^52^. The electronic structure was described using the GGA with the PBE functional, and the equations of motion were integrated using a time step of 1 fs. The evolution of specific chemical bonds was monitored by computing instantaneous bond lengths throughout the simulation. The resulting time-series data were analyzed to determine the distribution, mean values, fluctuations, and potential instances of significant bond elongation or dissociation.

## Supplementary information

Supplementary Information

Description of Additional Supplementary Files

Supplementary Data 1–5

Transparent Peer Review file

## Source data

Source Data

## Data Availability

Source data are provided with this paper. Any other data that support the findings of this study are also available from the corresponding authors on request. Source data are provided with this paper.
