# Supplementary material for: Non-solvating additives regulate zinc deposition through anion-gated Stern-layer competition
Source: Nat Commun. 2026 Aug 13;17:8260. doi: 10.1038/s41467-026-76659-1 (PMC13473634; doi:10.1038/s41467-026-76659-1)
Supplement: Supplementary file 1 [file 41467_2026_76659_MOESM1_ESM.pdf]

Supplementary Information

Non-Solvating Additives Regulate Zinc Deposition through Anion-Gated Stern-Layer Competition

Authors: Li Li[1], Hang Yang[2]*, Tengyu Yao[3], Yiming Zhang[1], Yicheng Tan[1], Chenglin Miao[1], Duo Chen[3], Jingyi Wang[2], Yunpeng Zhong[2], Xingsen Guo[4], Jianrui Feng[2], Adham Hashibon[5], Guangshe Li[6], Wei Han[1]*, Guanjie He[2]*

Affiliations:

[1] College of Physics, State Key Laboratory of Inorganic Synthesis and Preparative Chemistry, International Center of Future Science, Jilin University, Changchun 130012, China.

[2] Christopher Ingold Laboratory, Department of Chemistry, University College London, London, WC1H 0AJ, UK.

[3] Jiangsu Key Laboratory of Electrochemical Energy Storage Technologies, College of Materials Science and Technology, Nanjing University of Aeronautics and Astronautics, Nanjing 210016, China.

[4] Department of Civil, Environmental and Geomatic Engineering, University College London, London WC1E 6BT, UK.

[5] Institute for Materials Discovery, University College London, 107 Roberts Building, London WC1E 7JE, UK.

[6] State Key Laboratory of Inorganic Synthesis and Preparative Chemistry, College of Chemistry, Jilin University, Changchun 130012, China.

* Corresponding author, E-mail: hang.yang@ucl.ac.uk, whan@jlu.edu.cn, g.he@ucl.ac.uk

‡ These authors contributed equally to this work.

This file includes:

Figs. S1 to S38

Tables S1 to S6

Supplementary References

Supplementary Figures

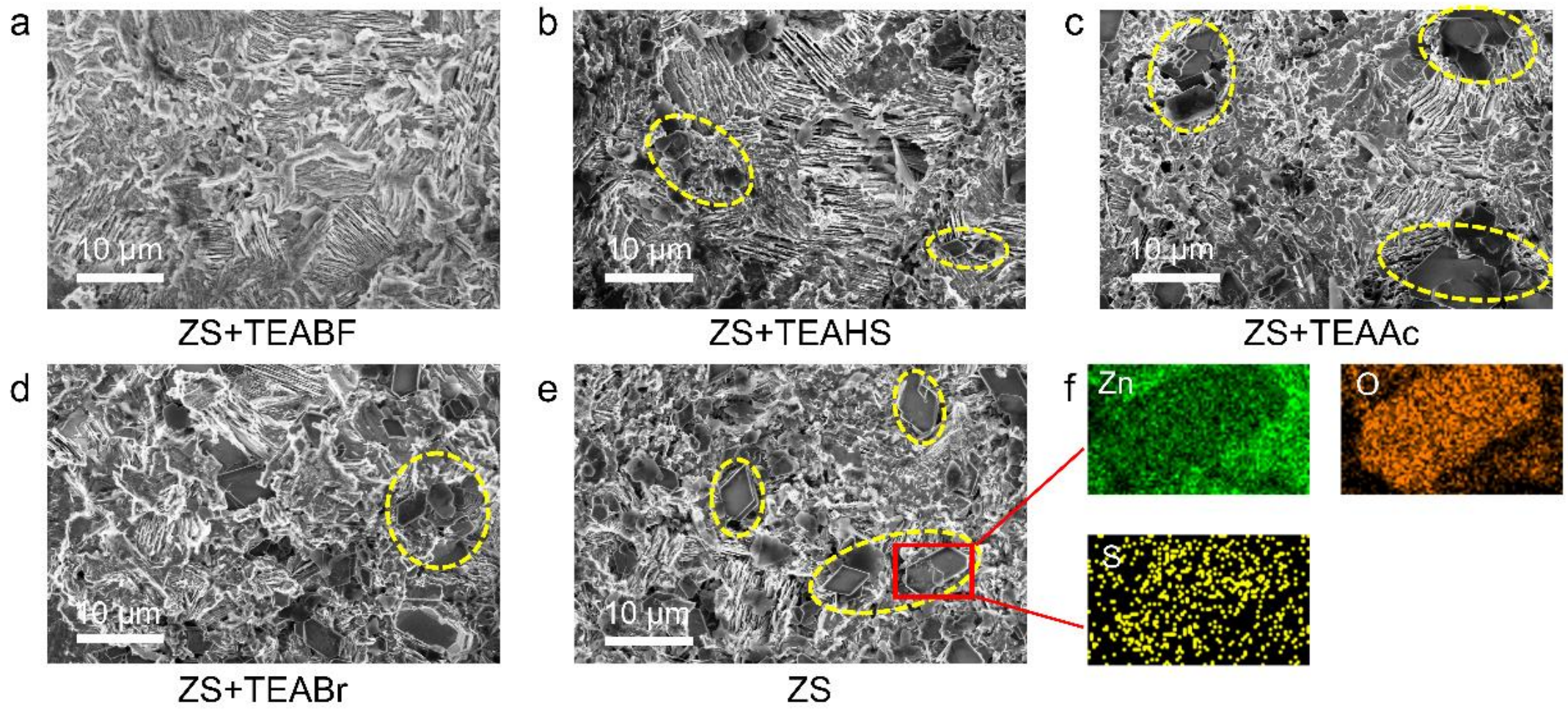

Supplementary Fig. 1. (a-e) SEM images of zinc electrodes in different electrolytes after 30 cycles under 5 mA cm^{-2}/ 2.5 mAh cm^{-2}. (f) The elemental mappings of the polygonal thin sheets on the surface of the cycled zinc electrode.

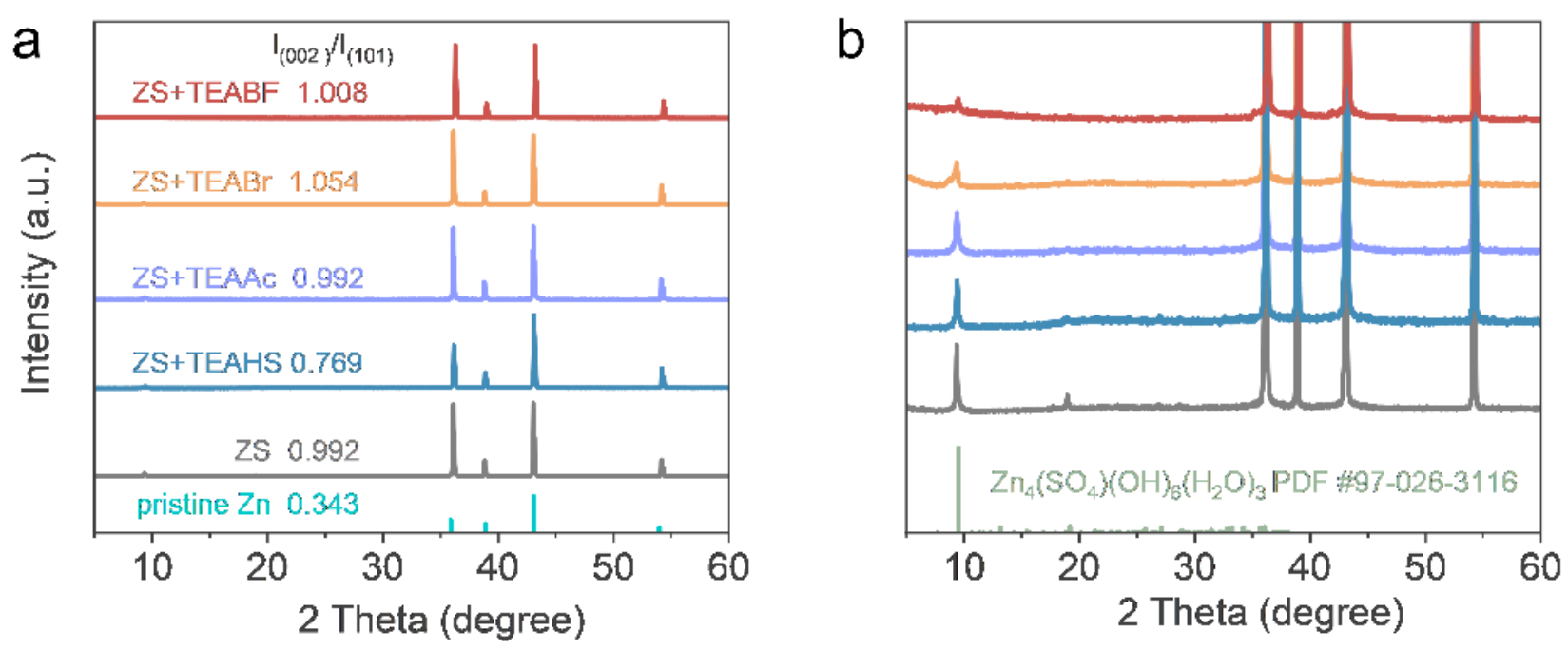

Supplementary Fig. 2. (a) XRD patterns and (b) locally magnified images of cycled zinc electrodes in various electrolytes after 30 Zn plating/stripping under 5 mA cm^{-2}/ 2.5 mAh cm^{-2}.

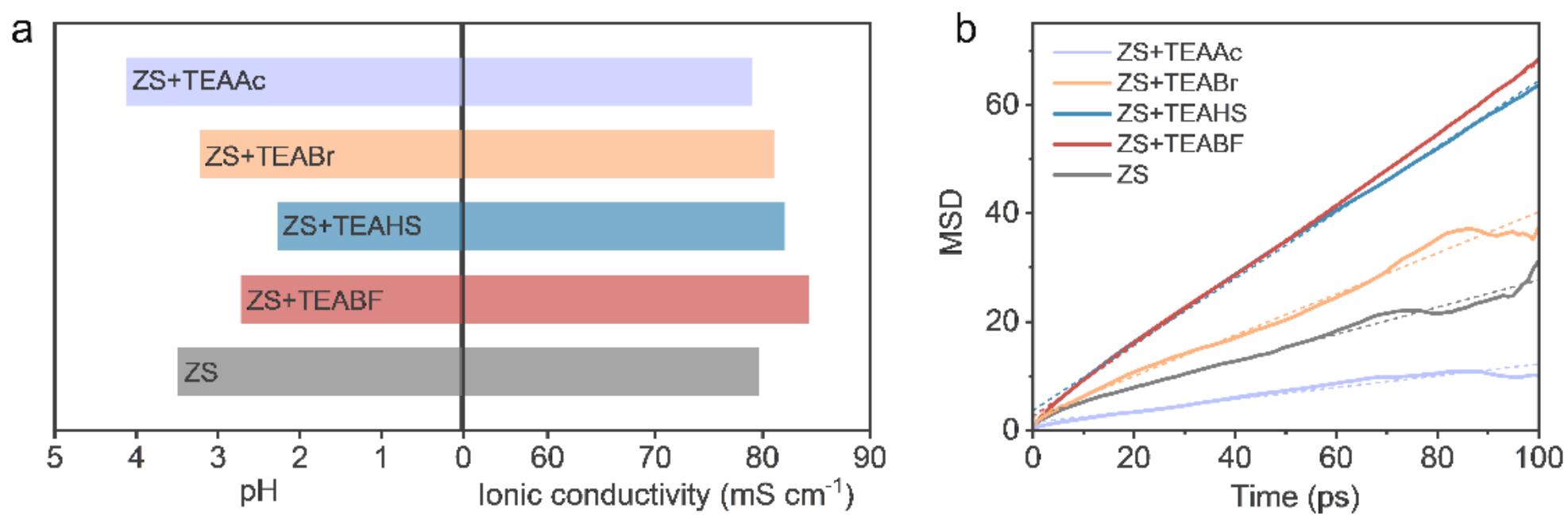

Supplementary Fig. 3. (a) The pH and ionic conductivity of the electrolytes with 2M $ZnSO_4$ and 10 mM quaternary amine salt additives composed of TEA^+ cation and different anions. (b) The mean square displacement (MSD) results of Zn^{2+} in different electrolytes.

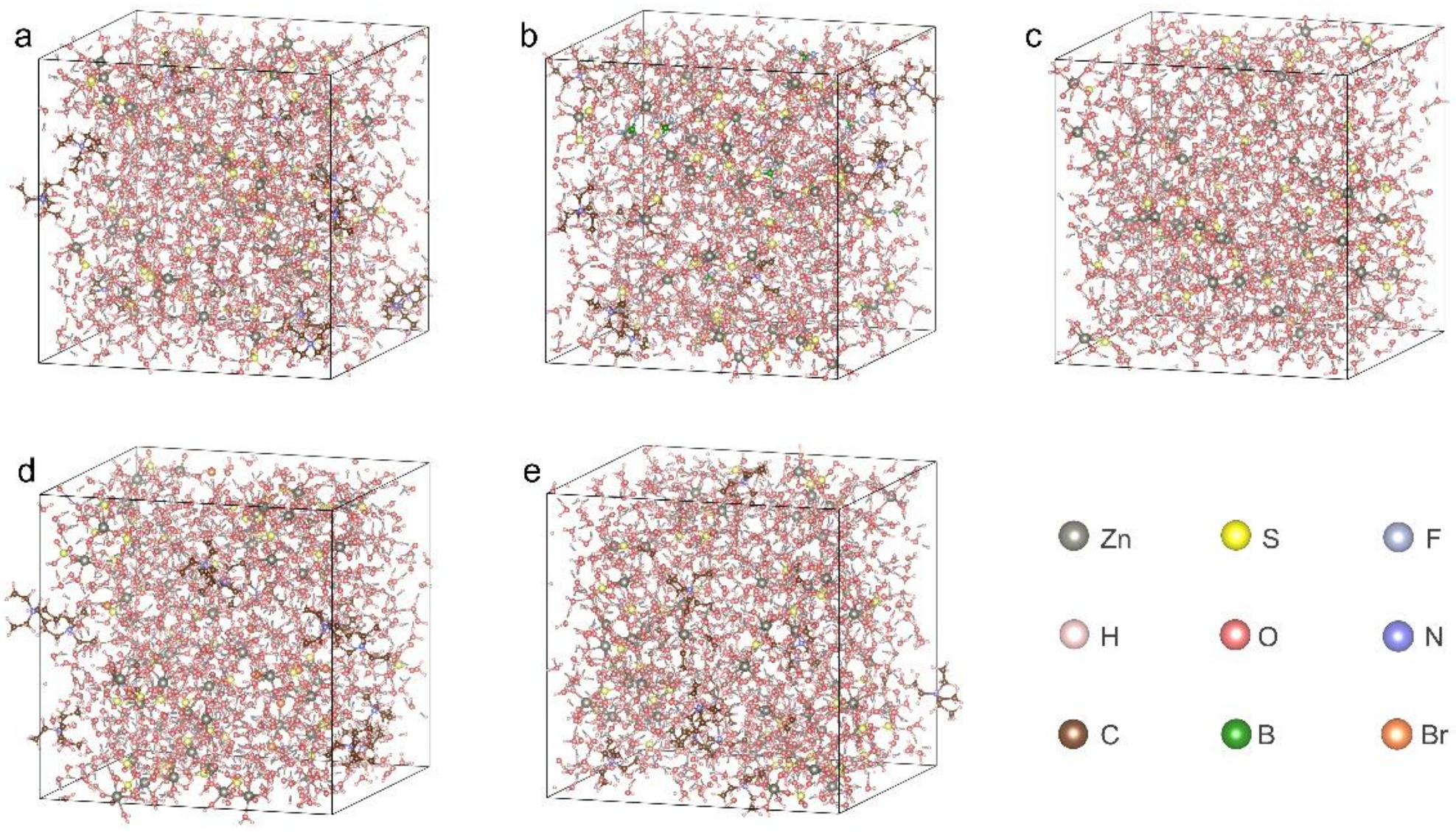

Supplementary Fig. 4. The MD results of (a) ZS+TEAHS, (b)ZS+TEABF, (c) ZS, (d) ZS+TEABr and (e) ZS+TEAAc electrolytes.

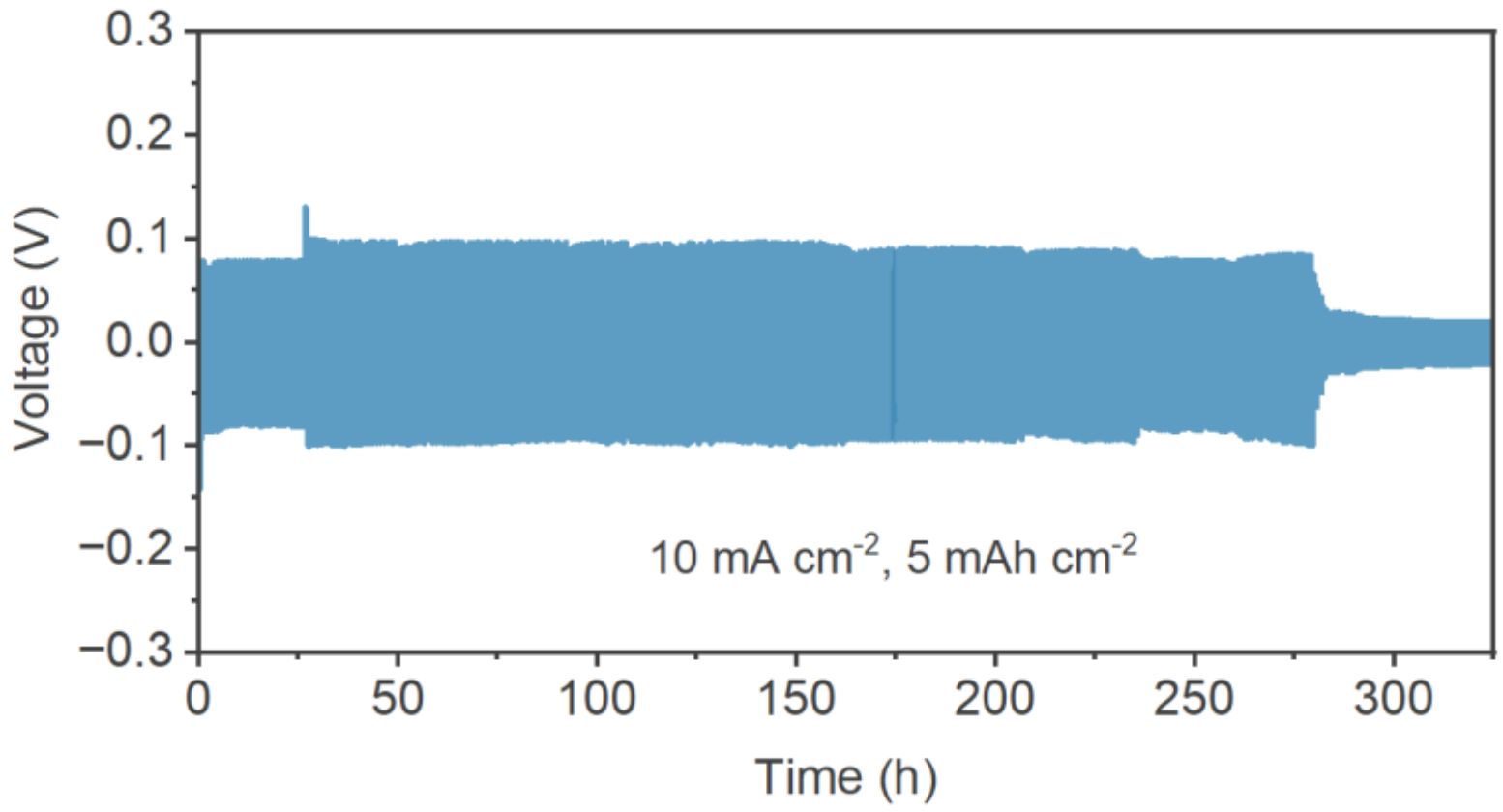

Supplementary Fig. 5. Cycle performance of the Zn||Zn symmetric cell in an alkalized ZS+TEAHS electrolyte.

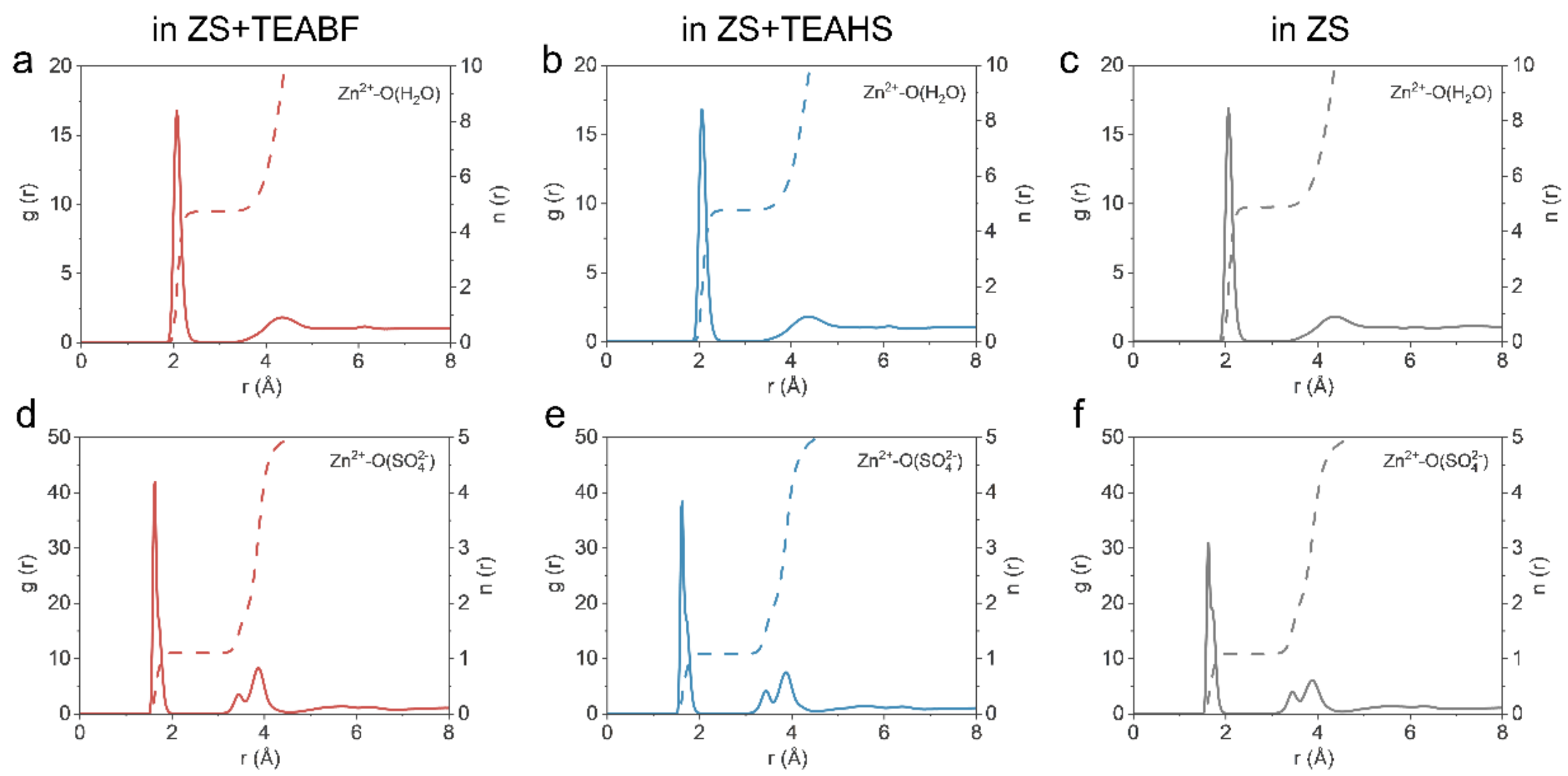

Supplementary Fig. 6. The RDF g(r) and coordination number n(r) plots for (a-c) Zn^{2+}-O (H_2O) and (d-f) Zn^{2+}-O (SO_4^{2-}) in different electrolytes.

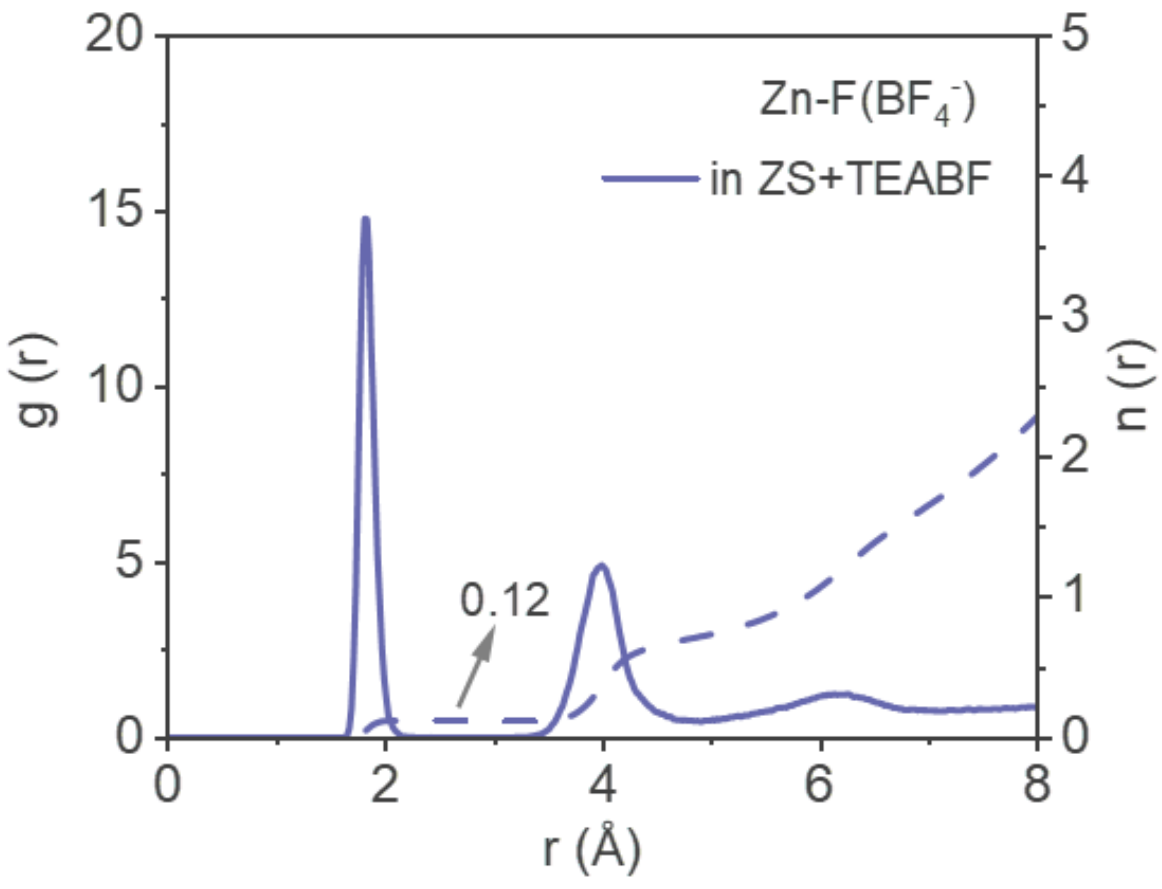

Supplementary Fig. 7. The RDF g(r) and coordination number n(r) plots for Zn^{2+}-F (BF_4^-) in ZS+TEABF electrolyte.

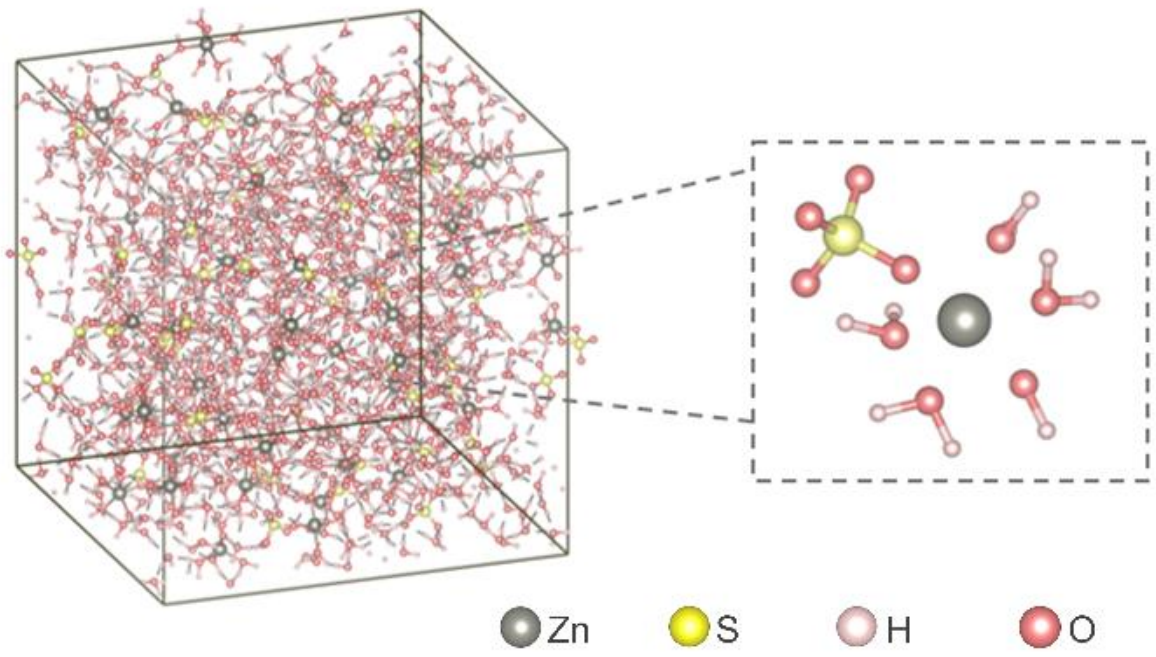

Supplementary Fig. 8. 3D snapshots obtained from MD simulation box in ZS electrolyte and partial enlarged snapshot representing Zn^{2+} solvation structure.

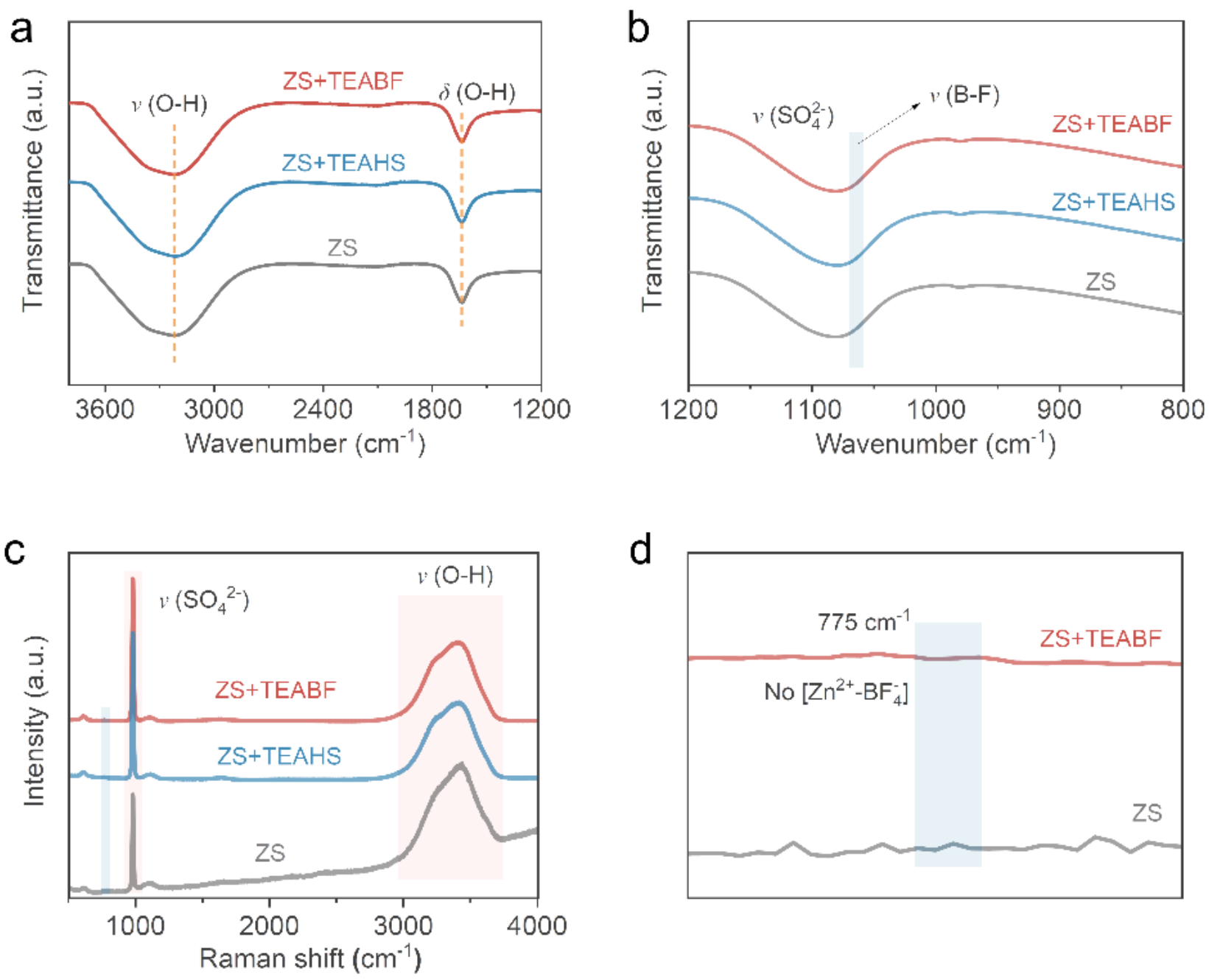

Supplementary Fig. 9. (a-b) FTIR, and (c-d) Raman spectra obtained from ZS, ZS+TEAHS, and ZS+TEABF electrolytes.

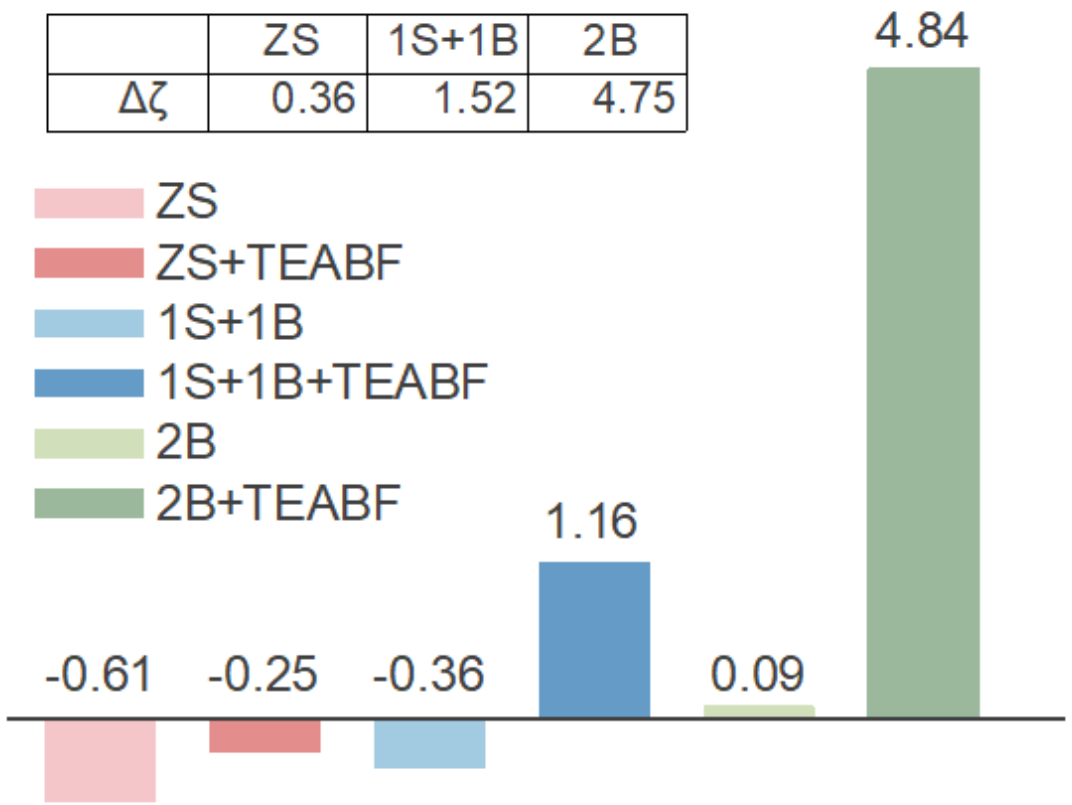

Supplementary Fig. 10. The zeta potential of Zn powder in electrolytes containing varying ratios of $Zn(BF_4)_2$ and $ZnSO_4$ ratios, measured under conditions with and without 0.01 M $TEABF_4$.

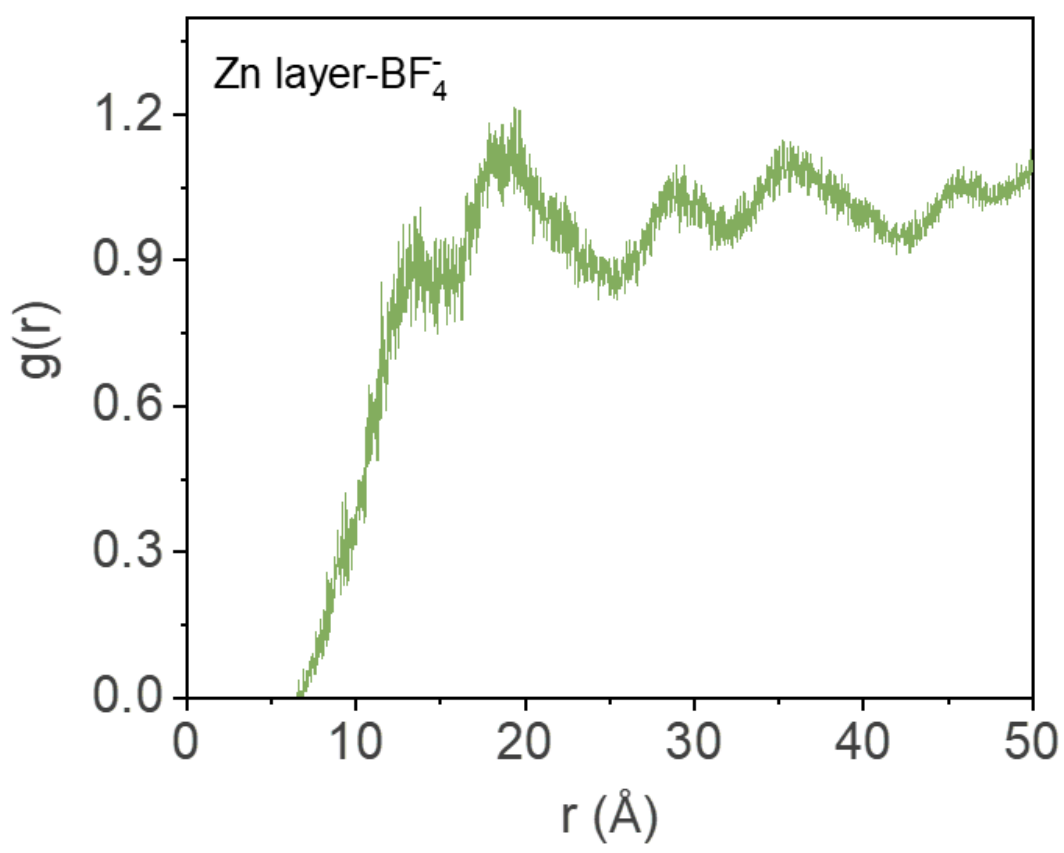

Supplementary Fig. 11. Distance-density distribution curve of BF_4^- on the surface of the Zn electrode in the ZS+TEABF electrolyte.

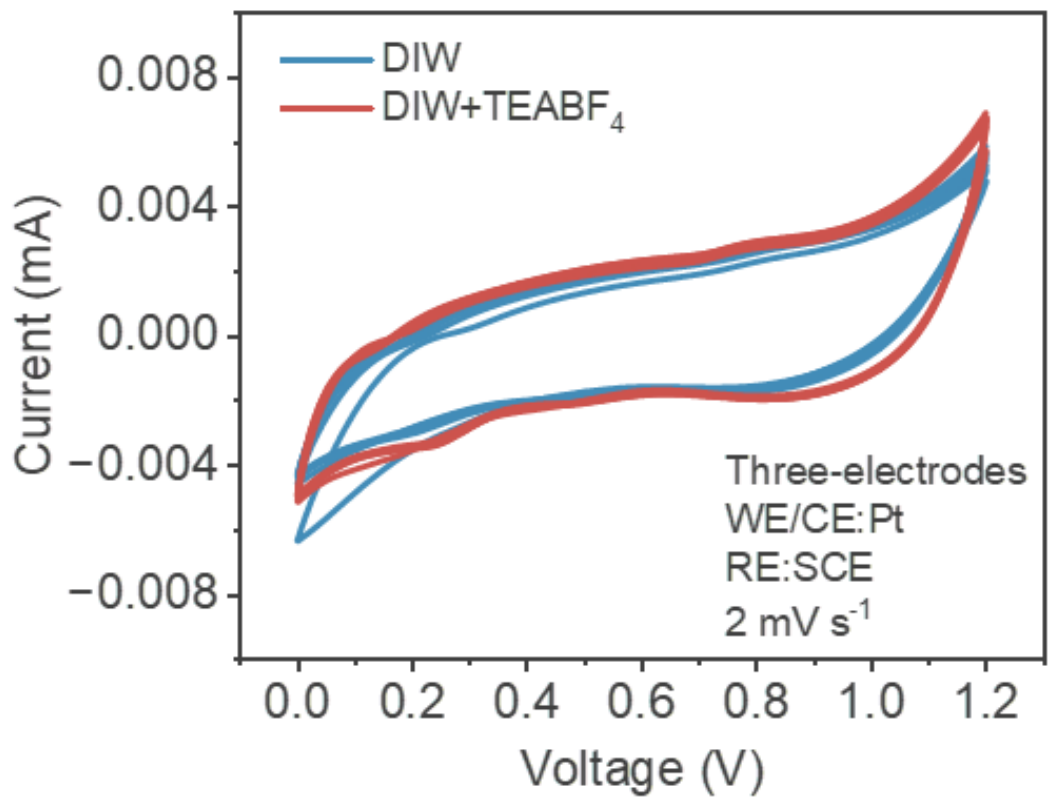

Supplementary Fig. 12. The CV curves of the DIW with or without 0.01 M $TEABF_4$ were measured within the potential range of 0–1.2 V.

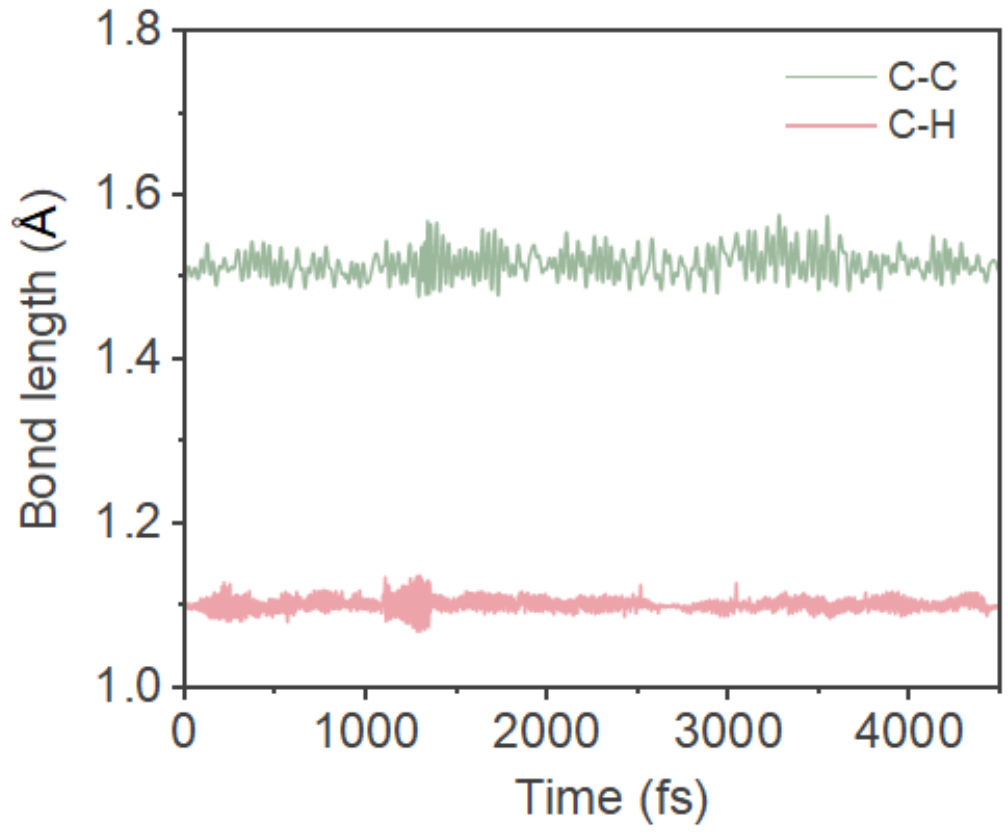

Supplementary Fig. 13. Changes in the C-C and C-H bond length during AIMD simulations.

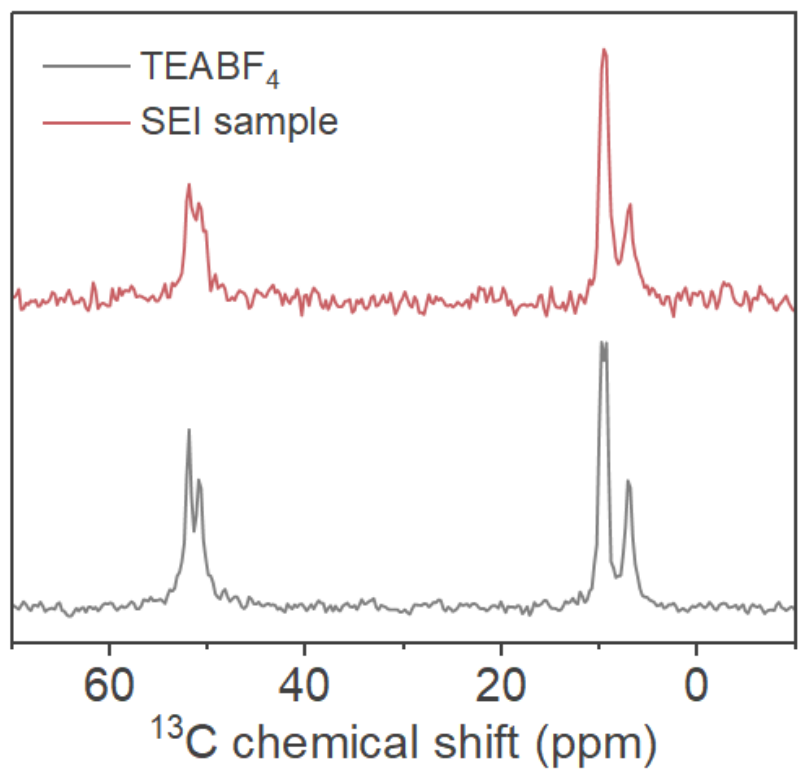

Supplementary Fig. 14. The ^{13}C NMR spectra of SEI sample derived from cycled zinc electrode and $TEABF_4$ powder. The zinc electrode was retrieved from Zn||Zn symmetric cell after 10 cycles under the condition of 5 mA cm^{-2}@2.5 mAh cm^{-2}.

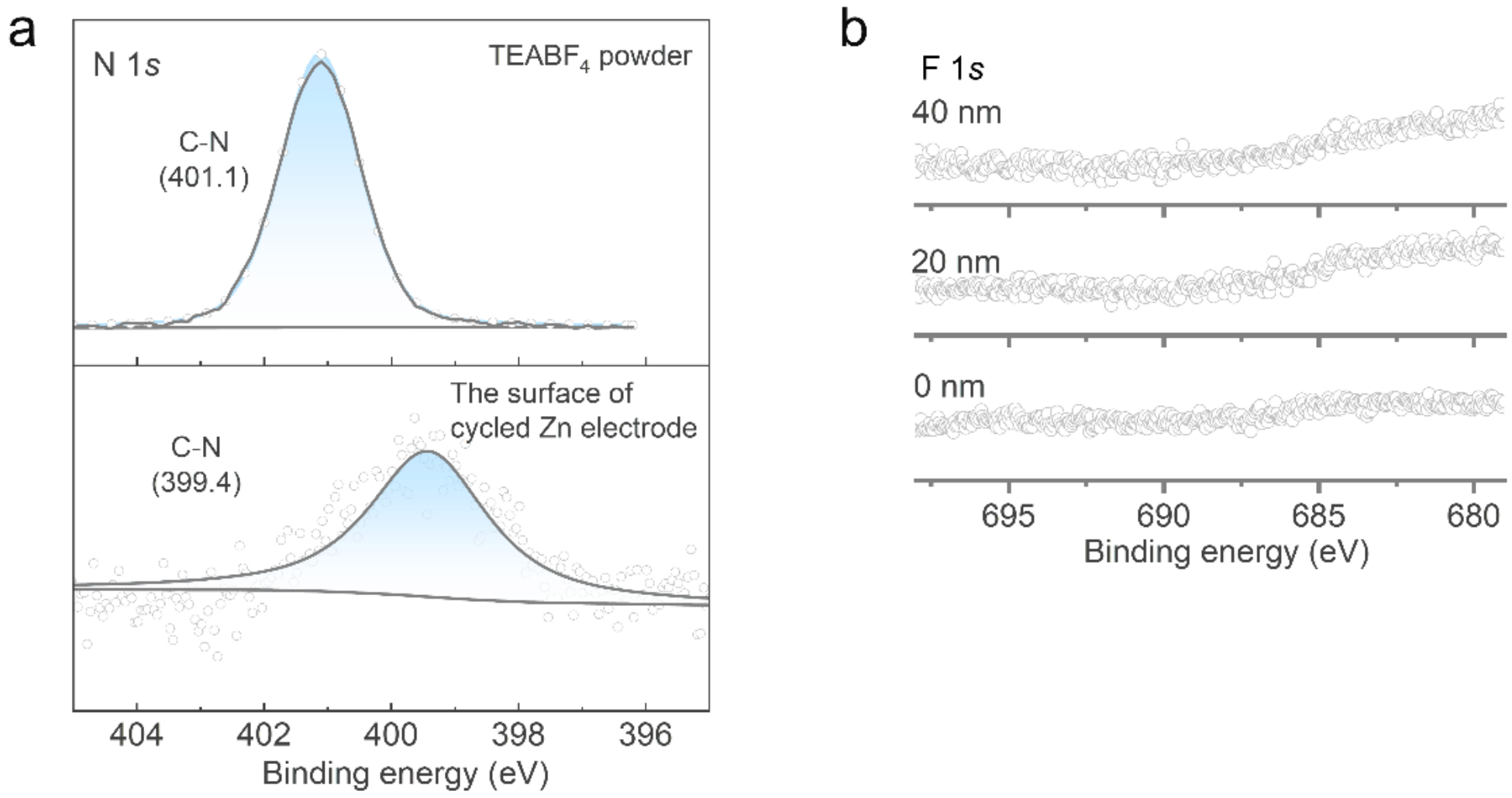

Supplementary Fig. 15. (a) N 1*s* spectral analysis of the $TEABF_4$ powder and cycled Zn electrode surface. (b) F 1*s* of cycled Zn electrode in ZS+TEABF electrolyte.

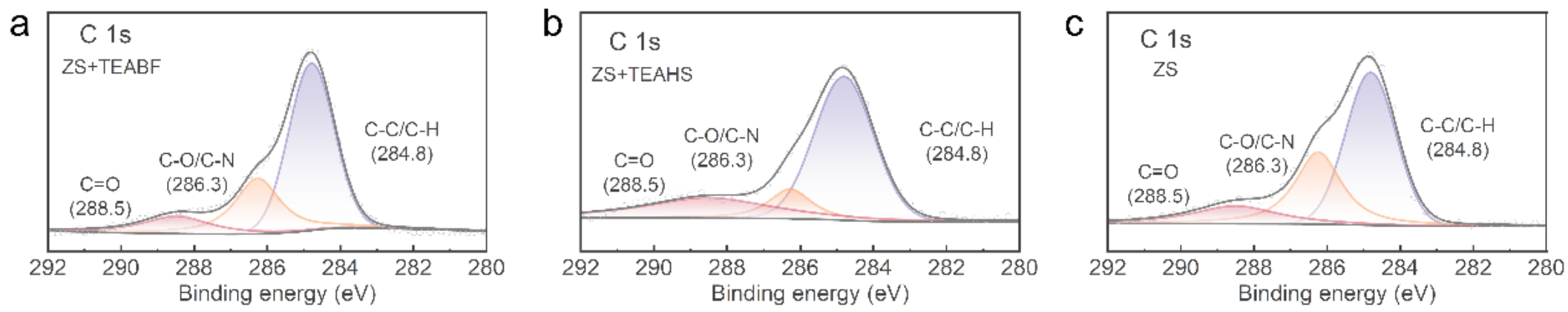

Supplementary Fig. 16. XPS spectra of C 1*s* for Zn electrode cycled in (a) ZS+TEABF, (b) ZS+TEAHS, and (c) ZS electrolytes.

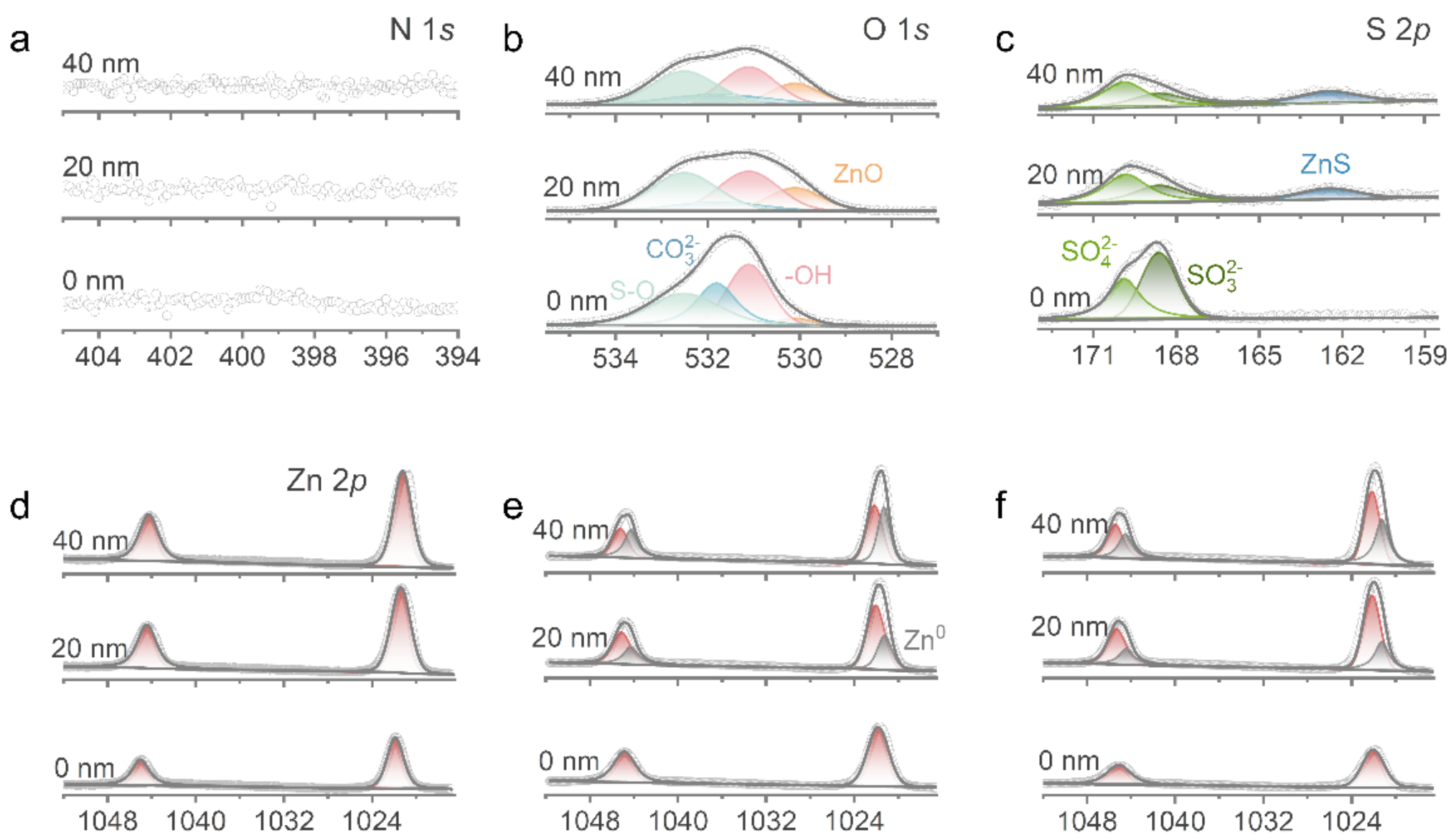

Supplementary Fig. 17. The XPS depth profile for (a) N 1*s*, (b) O 1*s*, (c) S 2*p* of Zn negative electrodes after 10 cycles at a current density of 2 mA cm^{-2} for 1 mAh cm^{-2} in ZS electrolyte. (d-f) Zn 2*p* of cycled Zn in (d) ZS+TEABF, (e) ZS+TEAHS and (f) ZS electrolyte.

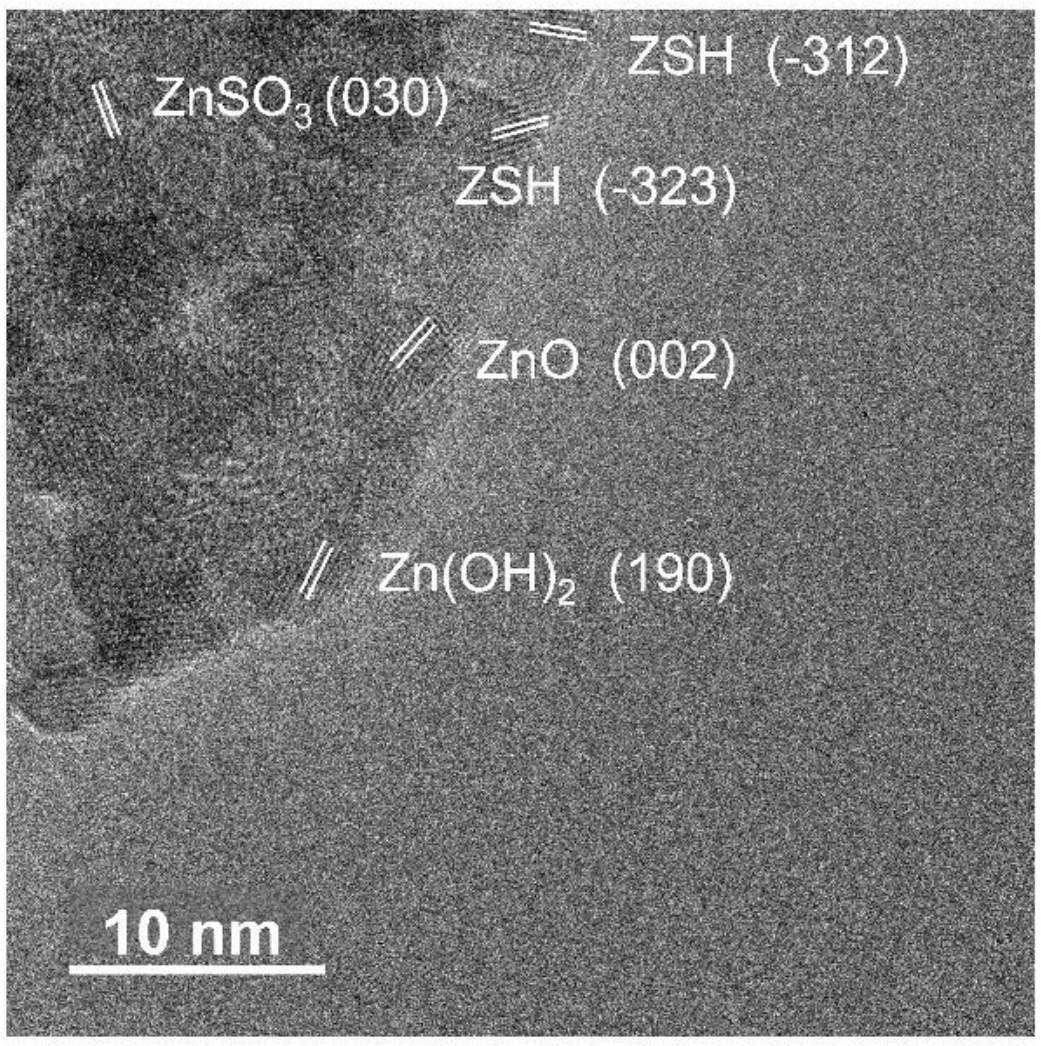

Supplementary Fig. 18. HRTEM image of the formed SEI in the ZS+TEAHS electrolyte.

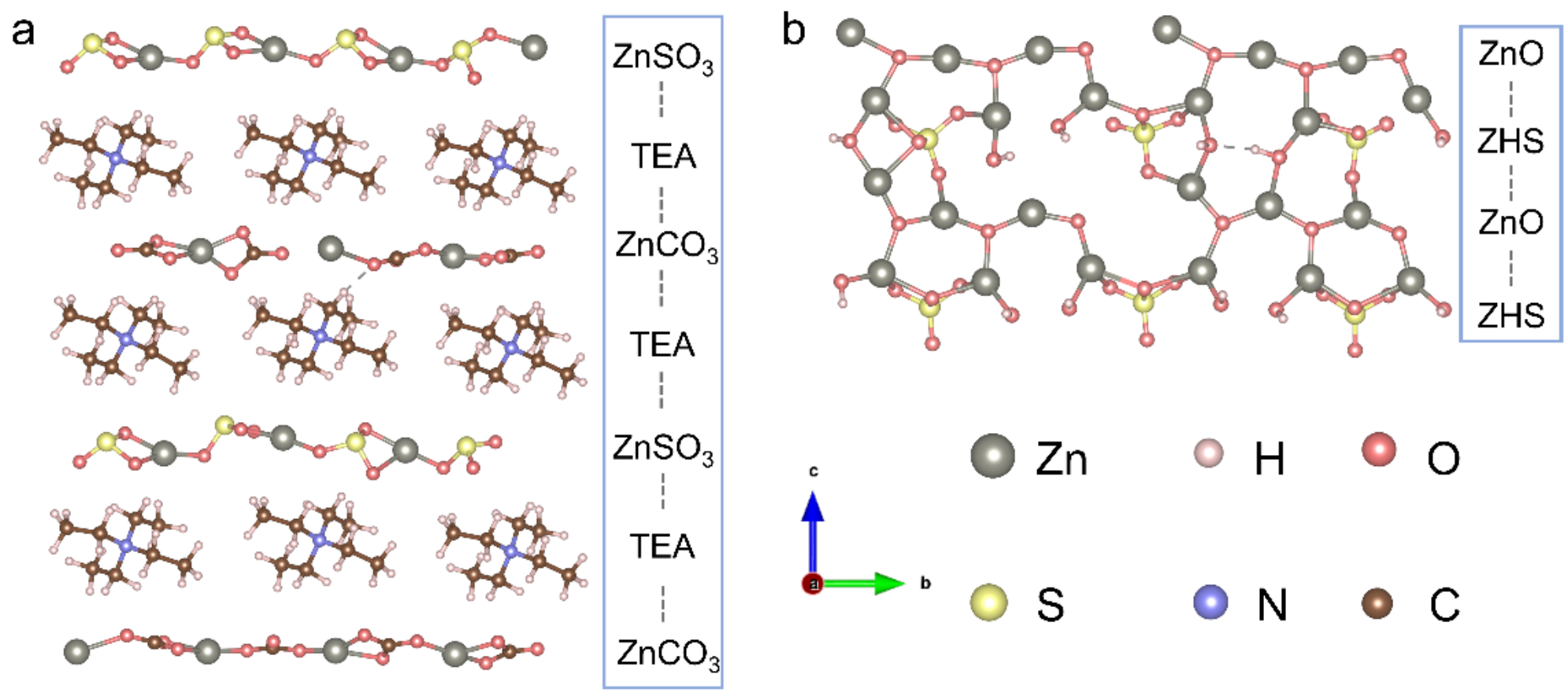

Supplementary Fig. 19. Top view of the simplified SEI molecular models derived in (a) ZS+TEABF and (b) ZS+TEAHS electrolytes.

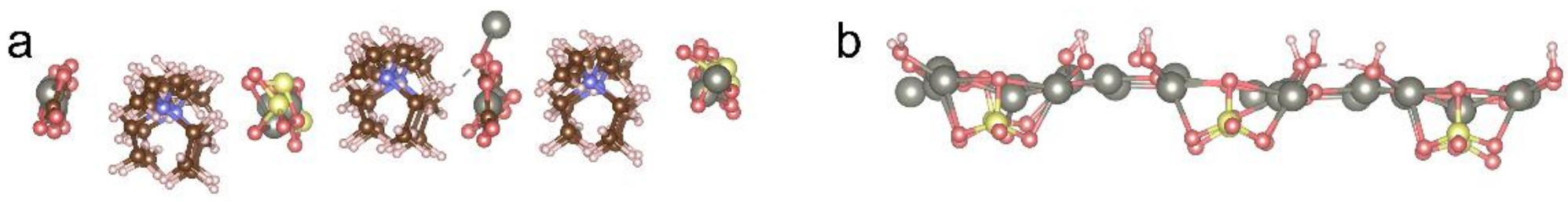

Supplementary Fig. 20. Main view of the simplified SEI molecular models derived in (a) ZS+TEABF and (b) ZS+TEAHS electrolytes.

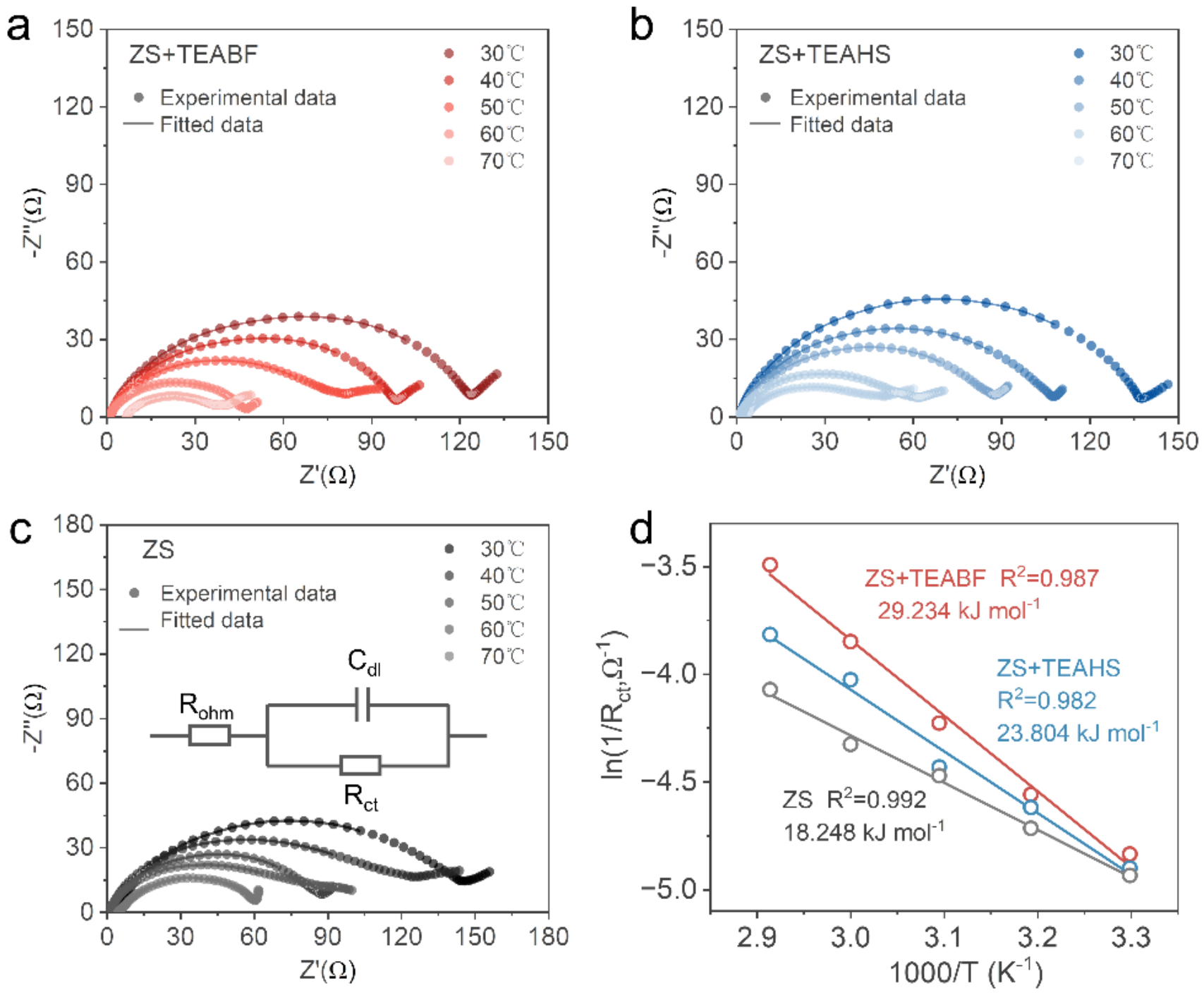

Supplementary Fig. 21. (a-c) EIS test of symmetric batteries in different electrolytes at different temperatures and (d) the activation energy fitted by the Arrhenius equation.

According to Arrhenius equation[1],

$$\frac{1}{R_{ct}}=A\exp(-\frac{E_a}{RT})$$

the activation energy (E_a) can be simulated by the change of charge transfer resistance (R_{ct}) at different temperatures, which can effectively evaluate the leaching kinetics of Zn^{2+}. Where A, R, and T stand for Arrhenius constant, molar gas constant, and test temperature respectively.

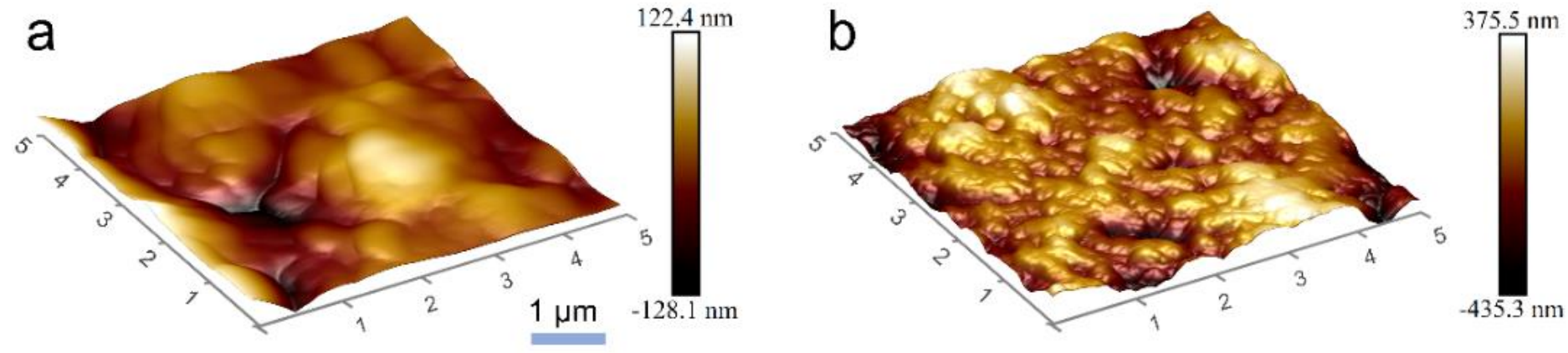

Supplementary Fig. 22. 3D topographical AFM images of the cycled Zn electrodes in (a) ZS+TEABF and (b) ZS+TEAHS electrolyte. The zinc electrodes utilized for the test were collected in the charged state after 50 Zn deposition/stripping cycles conducted at a current density of 2 mA cm^{-2} and areal capacity of 1 mAh cm^{-2}.

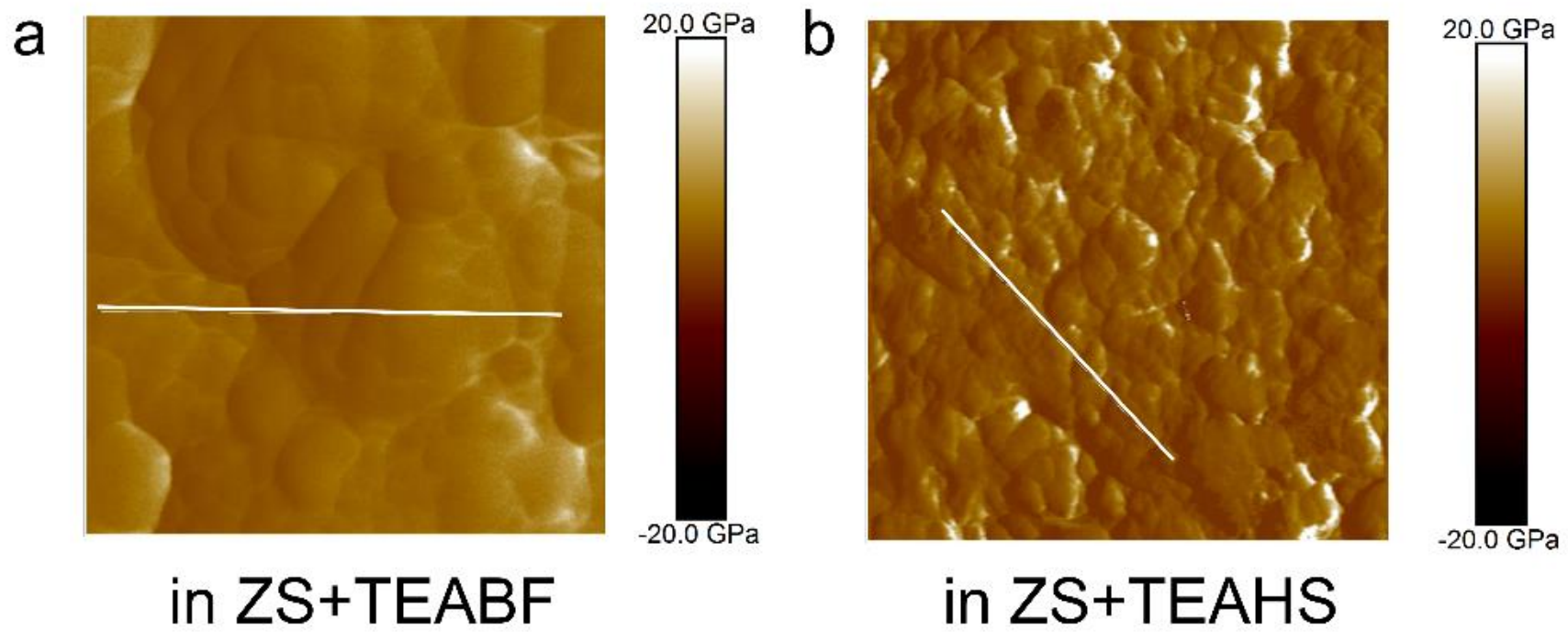

Supplementary Fig. 23. Selection diagram of the mechanical modulus distribution of the outside surficial SEI formed in (a) ZS+TEABF and (b) ZS+TEAHS electrolytes.

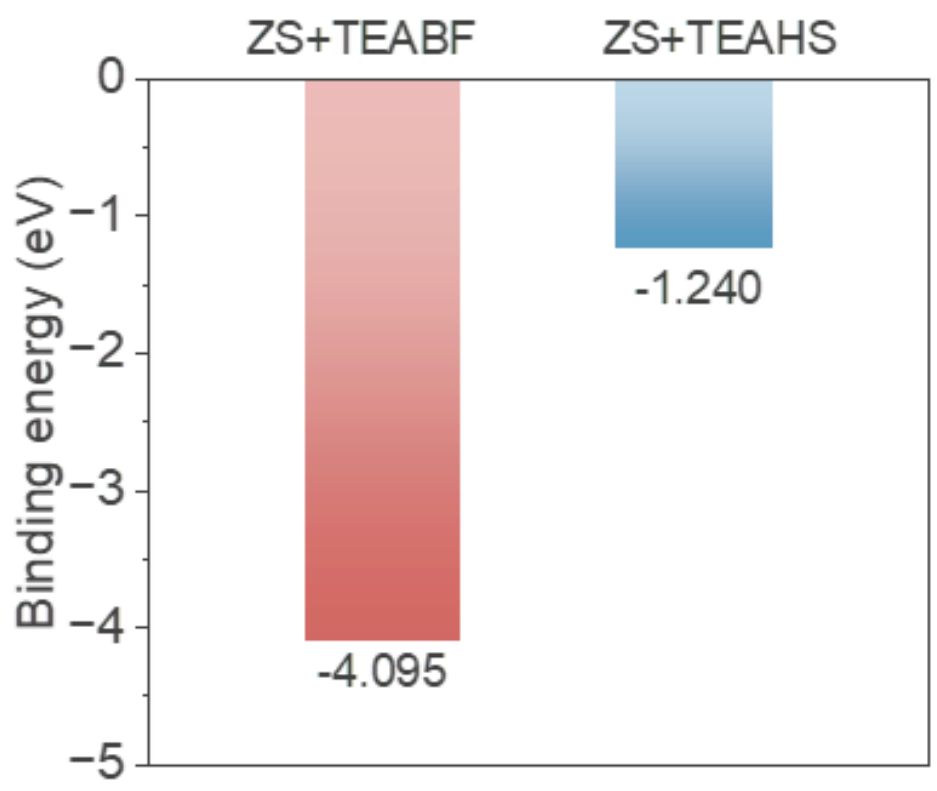

Supplementary Fig. 24. The binding energy calculation of the SEI components derived from different electrolytes.

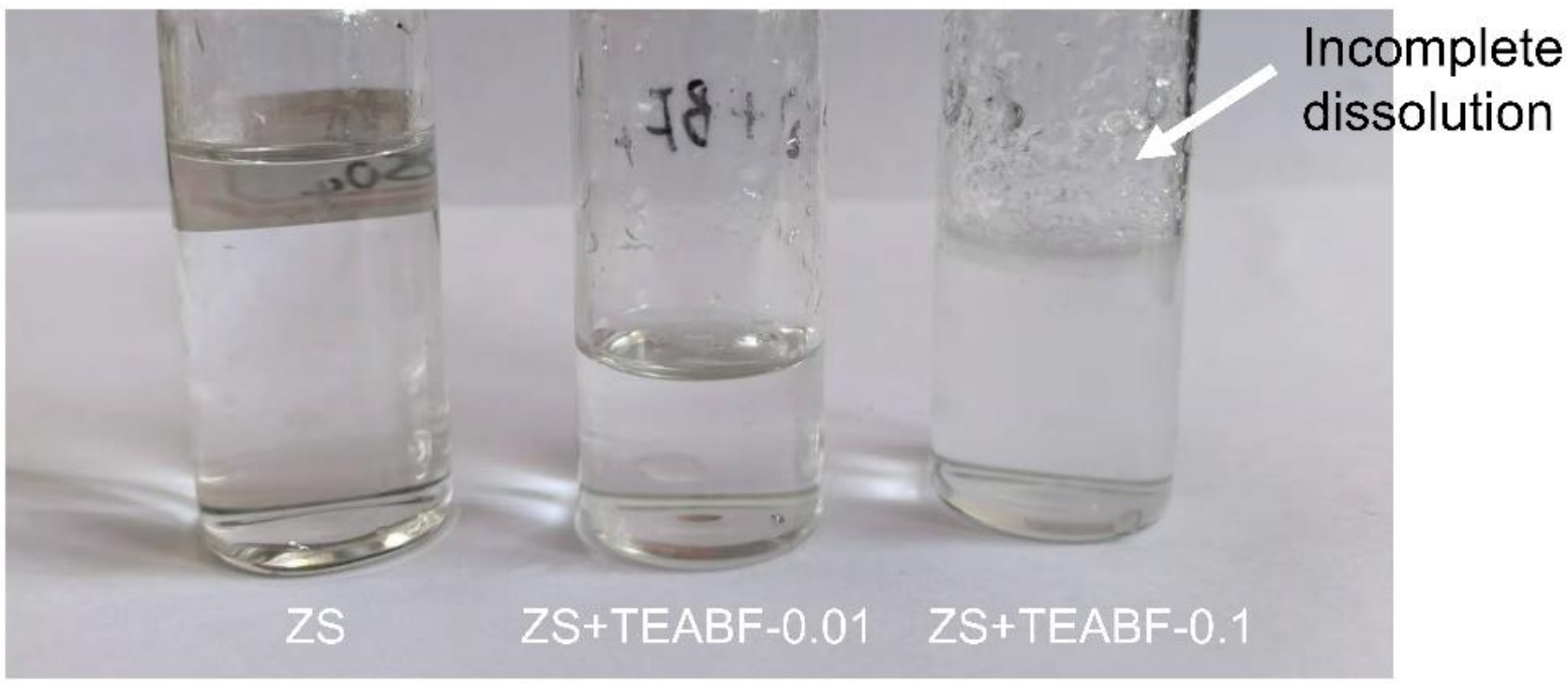

Supplementary Fig. 25. Optical photographs of electrolytes with different concentrations of additive.

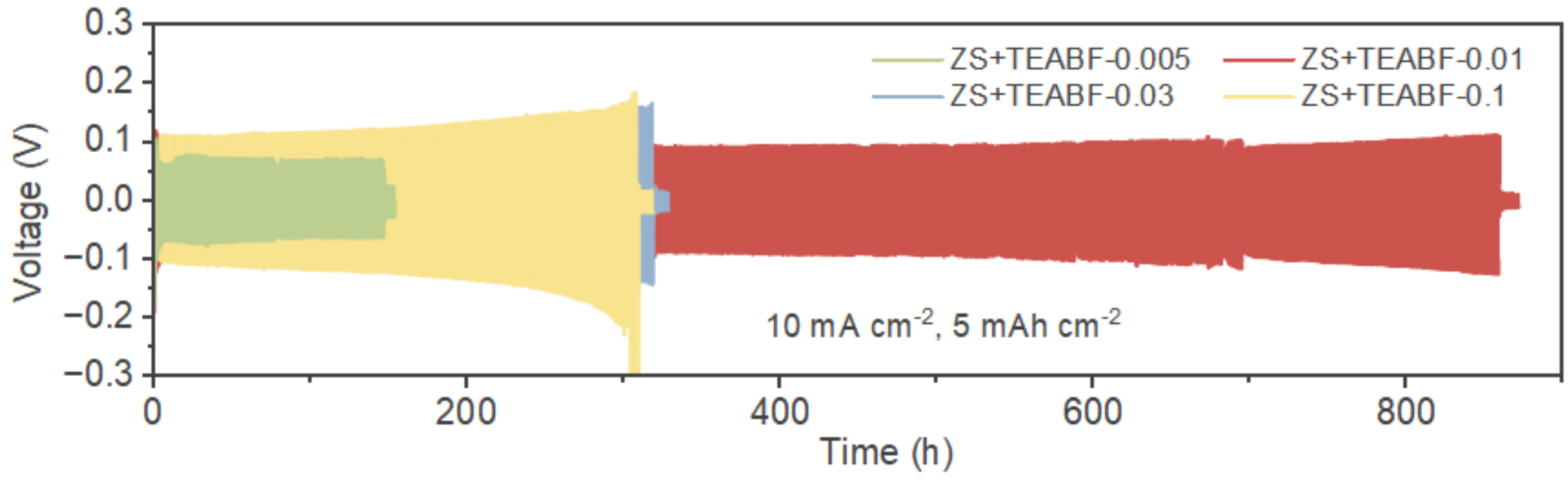

Supplementary Fig. 26. Long-term galvanostatic cycling performances of Zn||Zn cells in Zn+TEABF electrolytes containing varying concentrations of $TEABF_4$ at 10 mA cm^{-2}, 5 mAh cm^{-2}.

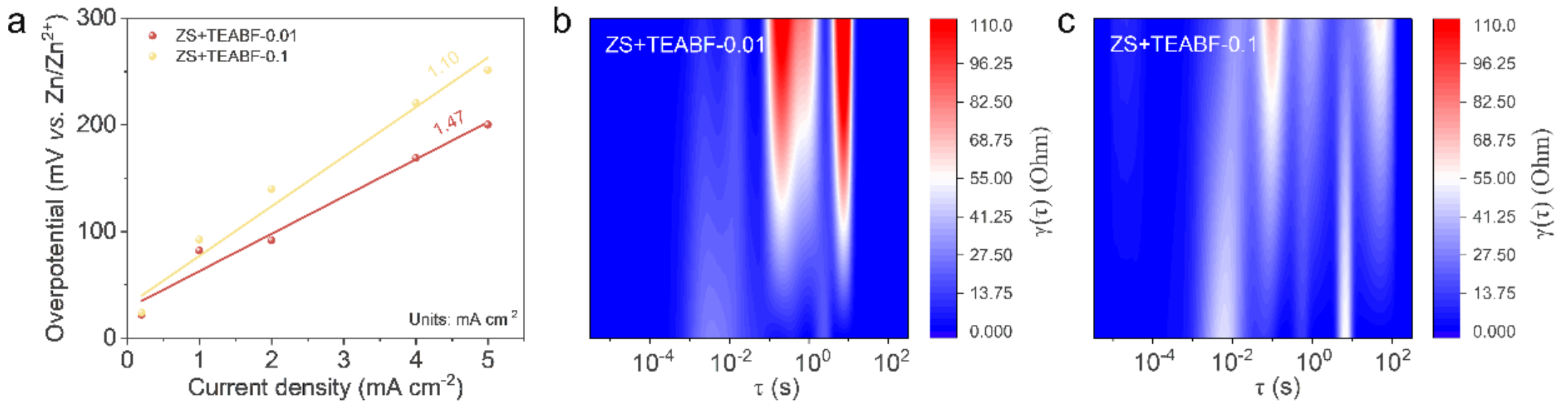

Supplementary Fig. 27. (a) Exchange current densities and (b-c) analysis of DRT of Zn negative electrodes in (b) ZS+TEABF-0.01 and (c) ZS+TEABF-0.1 electrolyte. The DRT data was acquired from the EIS results measured subsequent to the Zn||Zn symmetric cells undergoing 1 and 5 cycles of Zn plating/stripping at a current density of 2 mA cm^{-2} and capacity density of 1 mAh cm^{-2} under 25 °C.

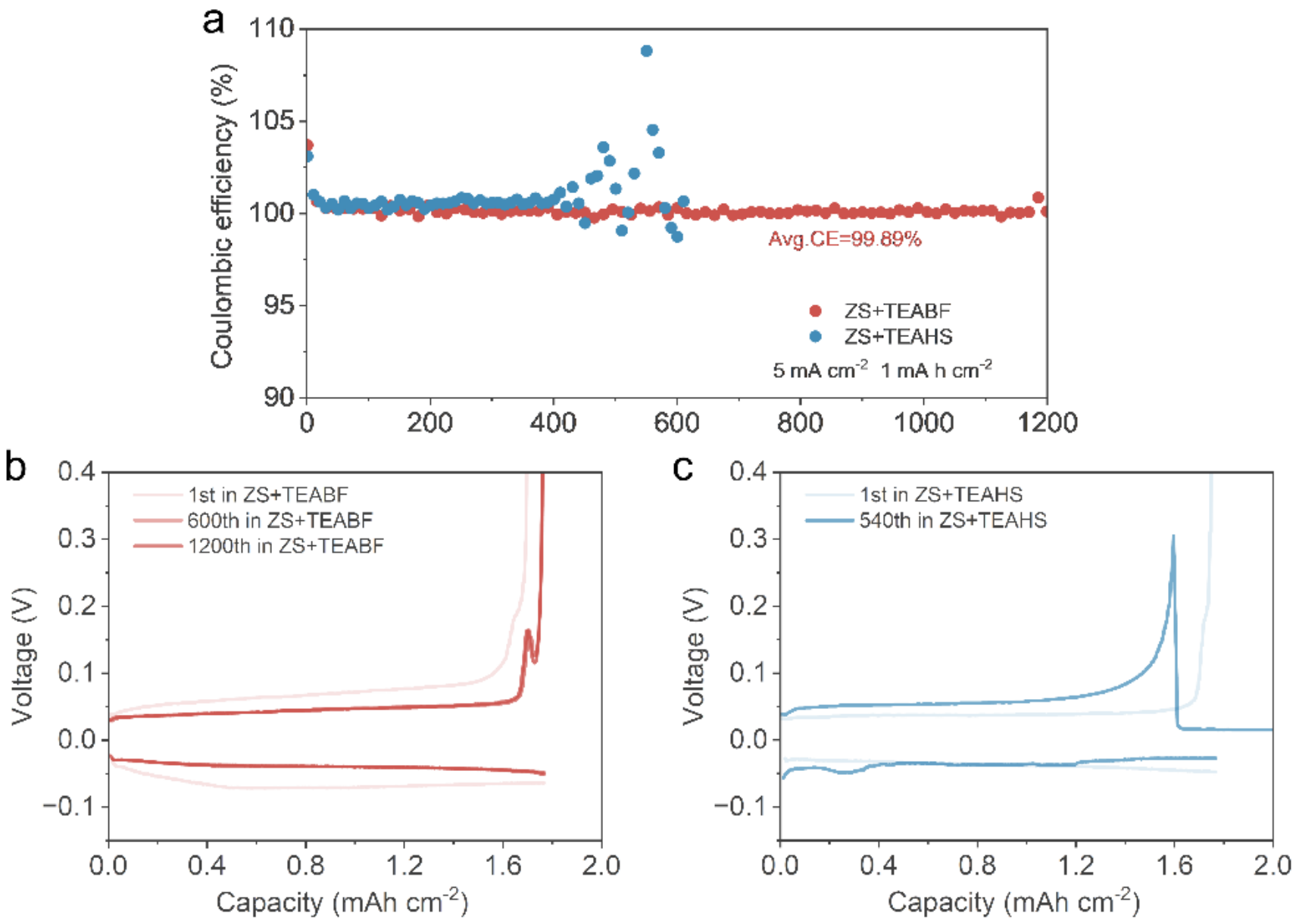

Supplementary Fig. 28. (a) Coulombic efficiency (CE) measurements of Zn||Cu cells in different electrolytes. (b-c) Enlarged voltage curves of Zn||Cu asymmetric cells with different cycle number.

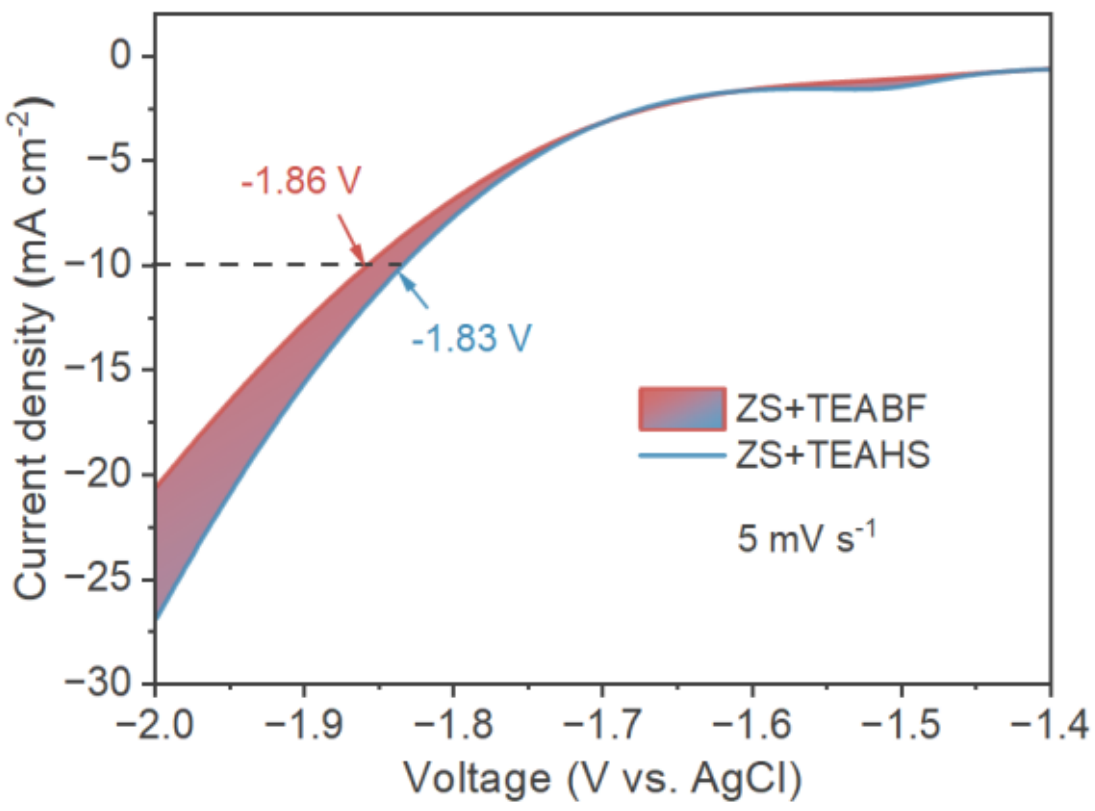

Supplementary Fig. 29. LSV curves of Zn foil in different electrolytes at a scan rate of 5 mV s^{-1}.

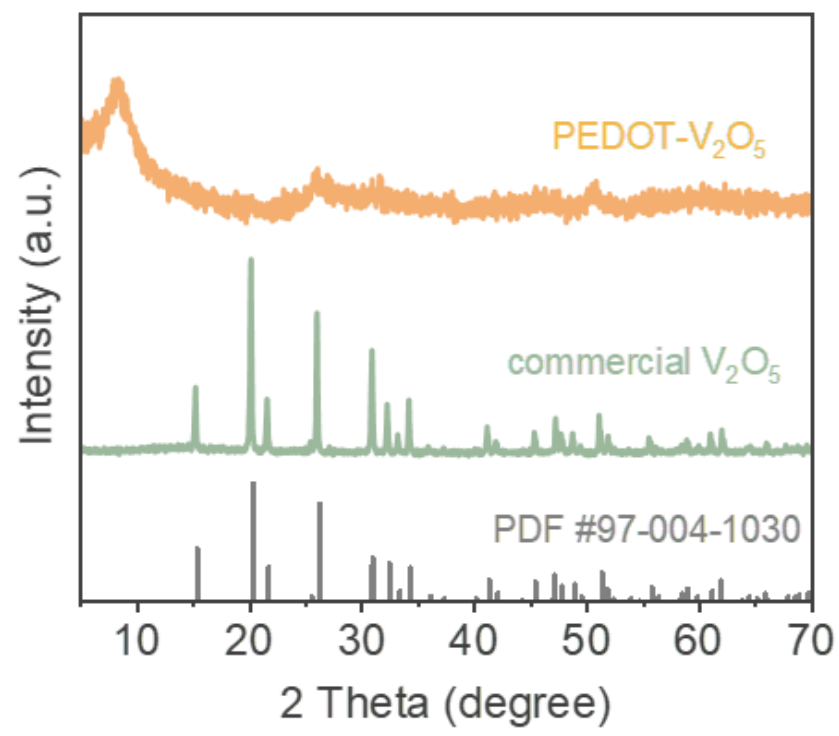

Supplementary Fig. 30. XRD pattern of the PEDOT-V_2O_5.

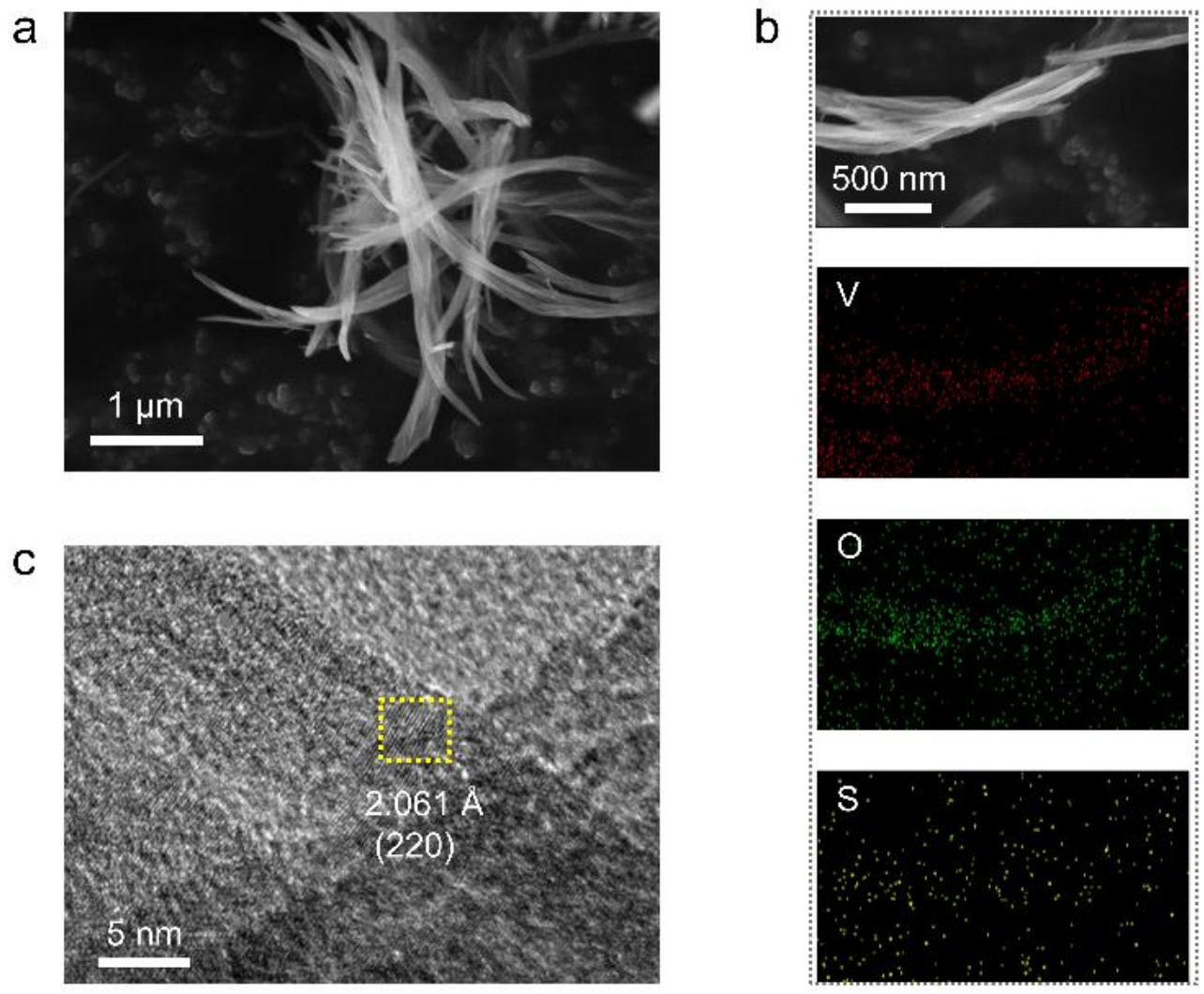

Supplementary Fig. 31. (a) SEM image and (b) EDS elemental mapping of the PVO. (c) The lattice spacing of the V_2O_5 (220) plane increases from 2.044 to 2.061 Å due to the intercalation and polymerization of EDOT.

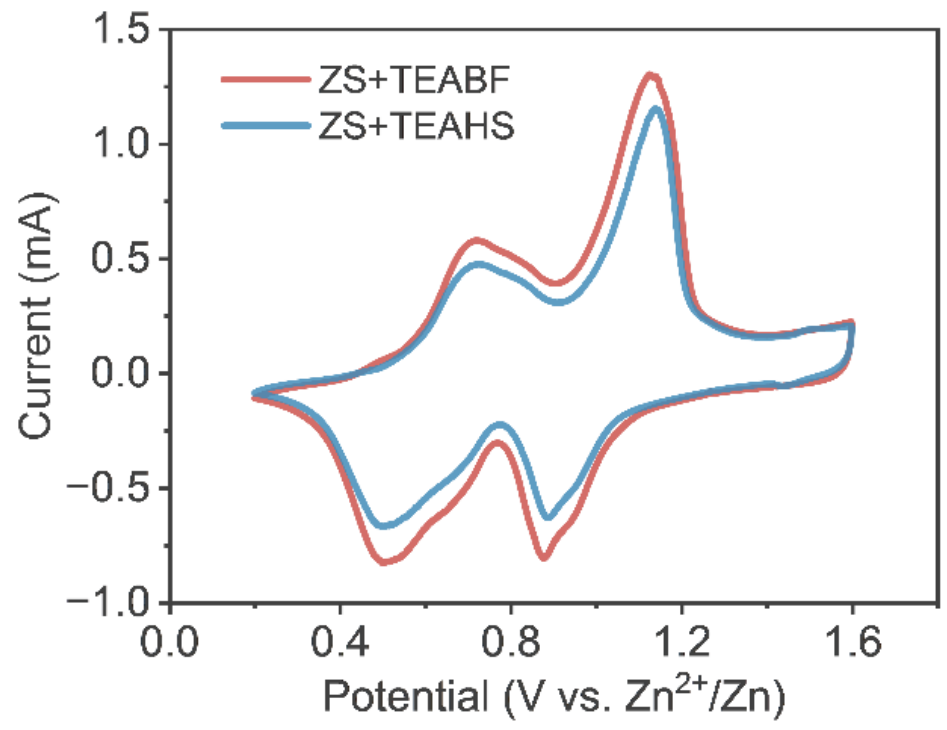

Supplementary Fig. 32. CV curves of Zn||PVO full cells in different electrolytes at a scan rate of 0.1 mV s^{-1}.

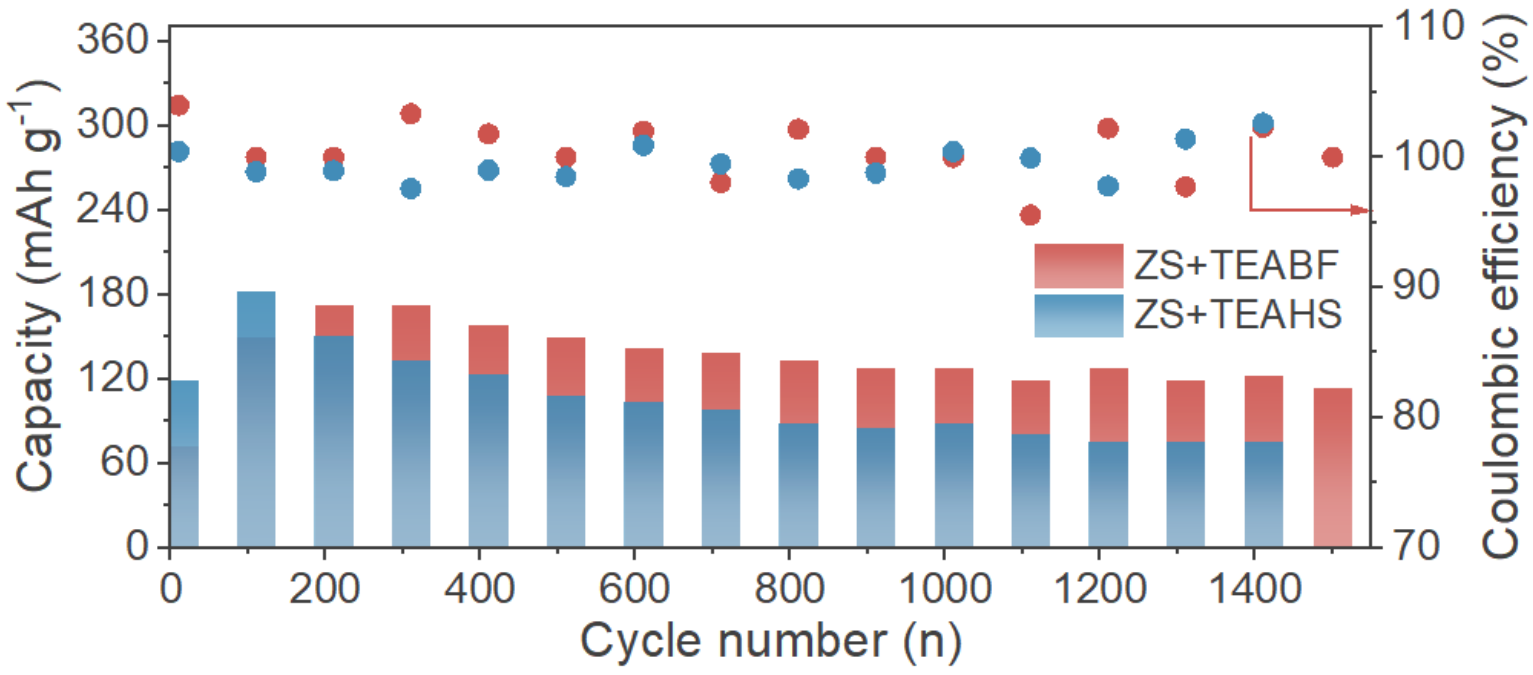

Supplementary Fig. 33. The cyclic behavior of Zn||PVO coin cells in various electrolytes at a current density of 10 A g^{-1}.

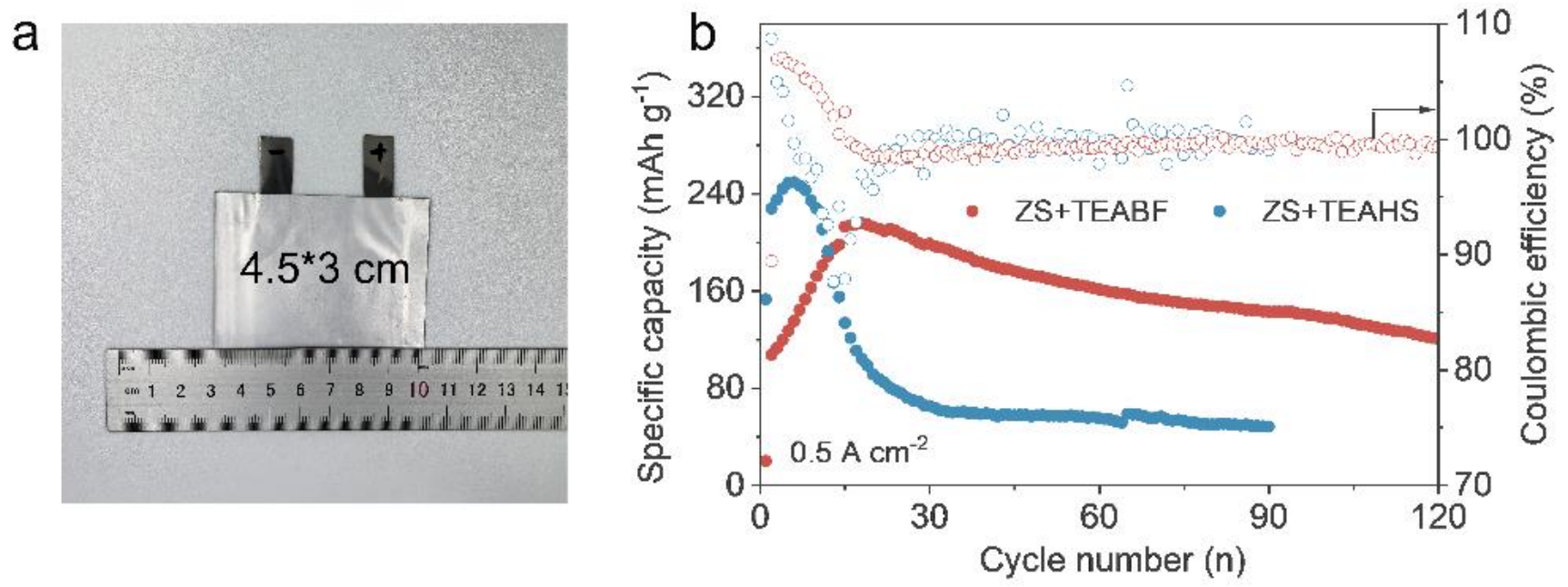

Supplementary Fig. 34. (a) The optical images of pre-deposited zinc on Ti||PVO pouch cell and (b) cycling performance at a current density of 0.5 mA cm^{-2}. The specific capacity is calculated based on the mass of the coated PVO.

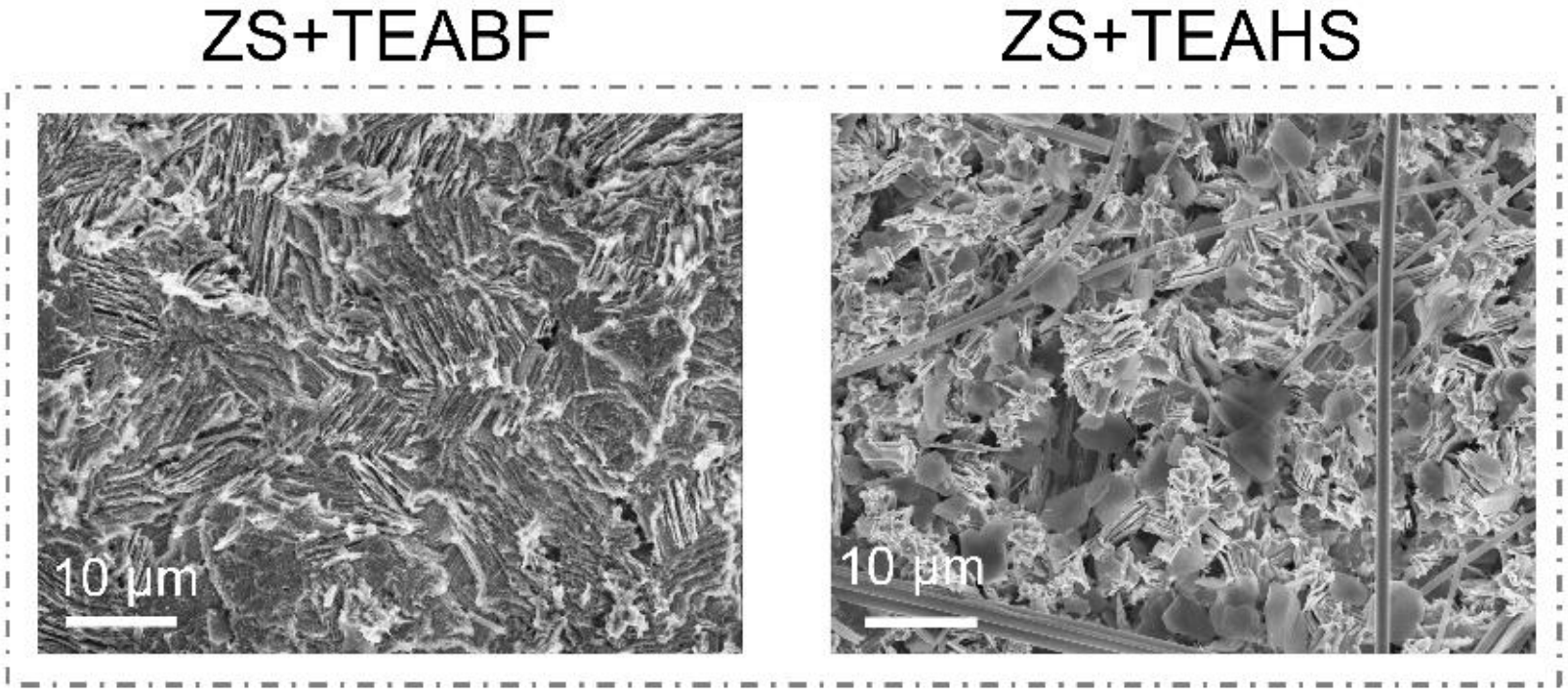

Supplementary Fig. 35. SEM images of Zn negative electrodes in the Zn||PVO coin cells after 50 cycles at 1 A g^{-1} using ZS+TEABF and ZS+TEAHS electrolyte.

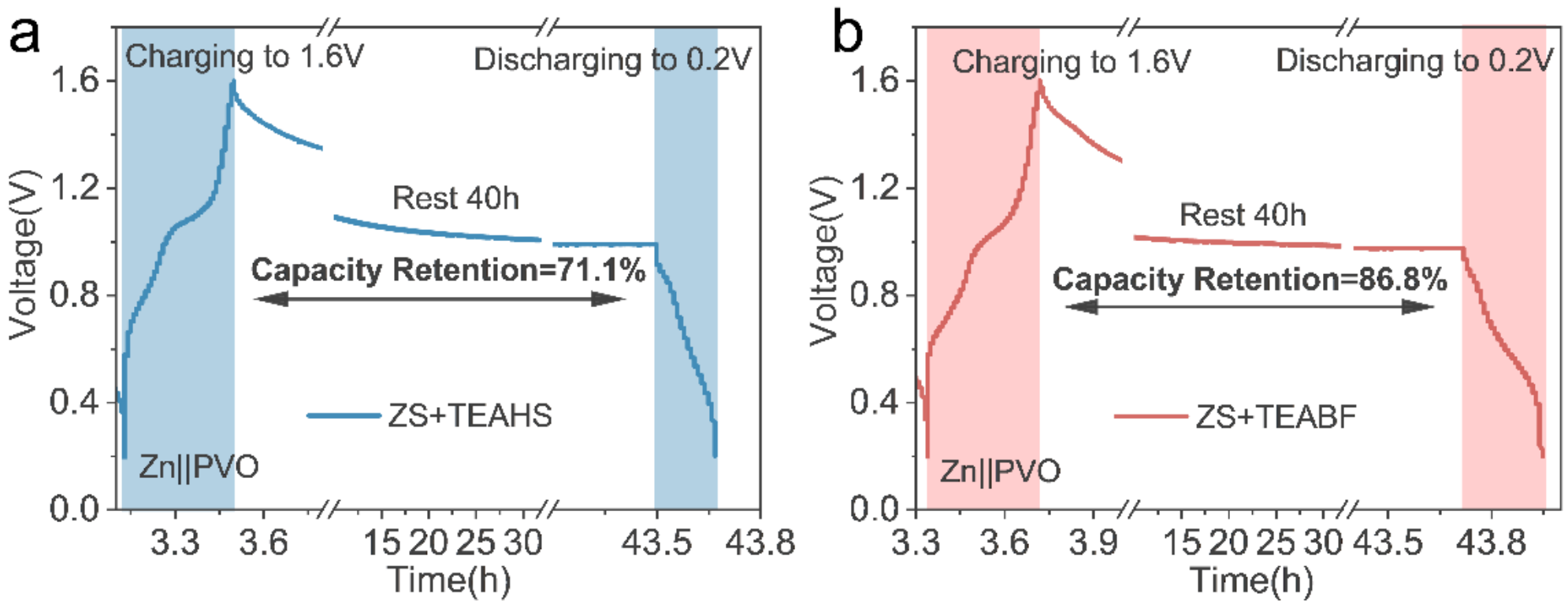

Supplementary Fig. 36. Zn||PVO coin cells using (a) ZS+TEAHS and (b) ZS+TEABF electrolytes were first fully charged to 1.6 V at 1.0 A g^{-1}, and then the cells were rested for 40 h, followed by full discharging to 0.2 V.

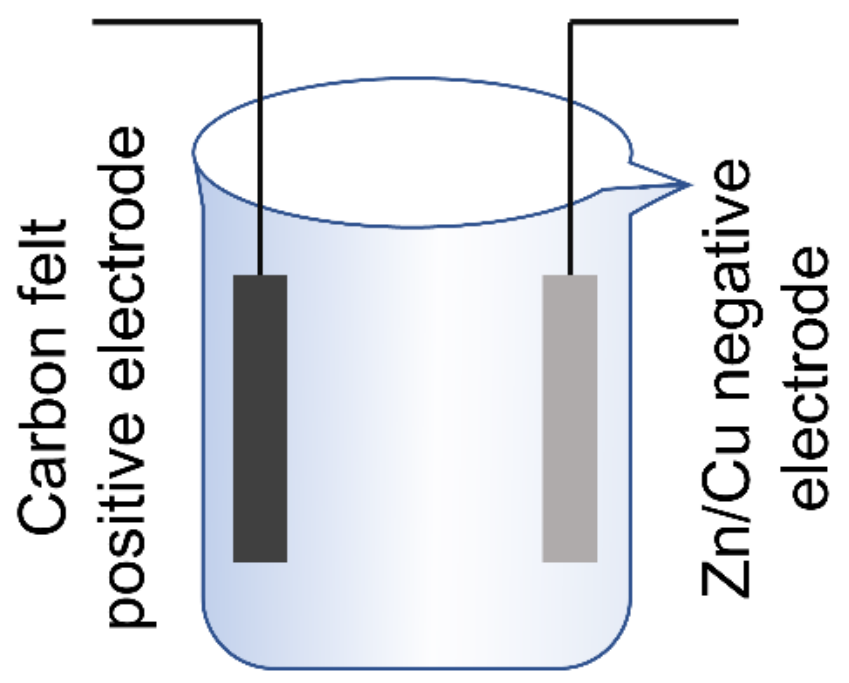

Supplementary Fig. 37. Schematic diagram of a self-assembling beaker type Zn-Mn battery.

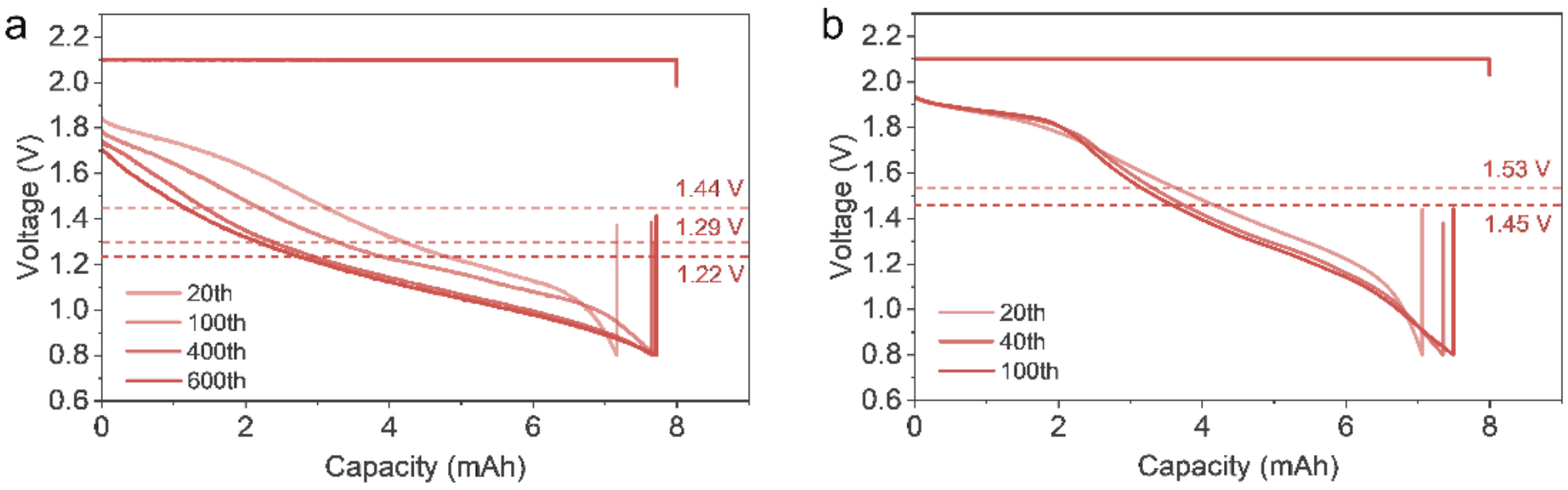

Supplementary Fig. 38. The voltage profile of the Mn-based (a) initially positive electrode-free cell and (b) initially dual-electrode-free cells in the ZS+TEABF electrolyte.

Supplementary Table 1. EDS analysis data of cycled zinc electrodes in different electrolytes after 30 cycles under 5 mA cm^{-2}/ 2.5 mAh cm^{-2}.

wt%	Zn	O	C	S
ZS+TEABF	83.8	4.5	11.3	0.3
ZS+TEAHS	82.1	12.6	2.9	2.4
ZS+TEAAc	85.7	9.7	2.9	1.8
ZS+TEABr	85.0	9.7	3.3	1.9
ZS	78.2	15.7	3.3	2.6

Supplementary Table 2. MSD results of Zn^{2+} in different electrolytes.

Electrolytes	D_{Zn2+} (Å^2 ps^{-1})	R^2
ZS+TEABF	0.652	0.99946
ZS+TEAHS	0.608	0.99817
ZS+TEABr	0.378	0.98563
ZS+TEAAc	0.108	0.94752
ZS	0.250	0.98488

Supplementary Table 3. Adsorption energy of different neutral species on Zn (002).

	TEA	Zn	H_2O	CH_3COO	Br	SO_4	BF_4
E_{ad} (eV)	-3.32	-0.64	-0.39	-2.62	-2.78	-2.92	-1.31

Supplementary Table 4. The fitted EIS results of the Zn||Zn symmetrical cells under different electrolytes and at different temperatures.

Electrolyte	Test temperature (°C)	R_{ct} (Ω)	Error (%)
ZS+TEABF	30	125.90	4.1743
	40	95.51	3.4923
	50	68.55	2.9608
	60	46.94	3.2446
	70	32.85	1.0332
ZS+TEAHS	30	134.40	3.4182
	40	101.40	2.5321
	50	84.08	2.7934
	60	56.05	3.3889
	70	45.43	2.1491
ZS	30	139.10	2.7566
	40	111.70	3.7900
	50	87.54	3.3820
	60	75.66	4.4864
	70	58.66	2.0716

Supplementary Table 5. Comparison of Zn||Zn cells performance from this work and other works based on trace additive strategies.

Additive	Concentration	Current density ($mA\ cm^{-2}$)	Capacity ($mAh\ cm^{-2}$)	Cycling time (h)
TA-Na[2]	10 mM	0.5	0.25	1500
TG[3]	10%	1<br>2	0.5<br>0.67	1100<br>335
AT[4]	2 Vol%	5	5	550
Ni^{2+} [5]	4 mM	1	1	900
TDFND [6]	5 mM	10	5	430
Zwitterion [7]	5 wt%	1<br>5<br>10	1<br>5<br>10	1800<br>700<br>600
NSQDs [8]	10 ppm	20	1	600
Gln [9]	0.3 M	1<br>3	1<br>3	700<br>700
Gly [10]	0.2 M	1<br>4	0.5<br>2	2000<br>600
$TEABF_4$ (this work)	10 mM	1<br>10	0.5<br>5	2400<br>850

Supplementary Table 6. ICP results of V concentration in different electrolytes after 50 cycles of Zn||PVO full cell at 1 A g^{-1}.

Electrolyte	Concentration (mg/L)
ZS+TEAHS	0.2015
ZS+TEABF	0.0802

Supplementary References:

1. He, J. et al. Synergistic effects of lewis acid–base and coulombic interactions for high-performance Zn–I_2 batteries. *Energy Envir. Sci.* **17**, 323-331 (2024).
2. Wan, J. et al. A double-functional additive containing nucleophilic groups for high-performance Zn-ion batteries. *ACS Nano* **17**, 1610−1621 (2023).
3. Liu, Z. et al. A dual-functional organic electrolyte additive with regulating suitable overpotential for building highly reversible aqueous zinc ion batteries. *Adv. Funct. Mater*. **34**, 2214538 (2023).
4. Cao, H. et al. Unraveling the solvation structure and electrolyte interface through carbonyl chemistry for durable and dendrite-free Zn anode. *Adv. Funct. Mater*. **33**, 2305683 (2023).
5. Dai, Y. et al. Reversible Zn metal anodes enabled by trace amounts of underpotential deposition initiators. *Angew. Chem., Int. Ed.* **62**, No. e202301192 (2023).
6. Li, T. et al. Engineering fluorine-rich double protective layer on Zn anode for highly reversible aqueous zinc-ion batteries. *Angew. Chem., Int. Ed.* **62**, No. e202314883 (2023).
7. Zha, Z. et al. Zwitterion as electrical double layer regulator to in-situ formation of fluorinated interphase towards stable zinc anode. *Energy Storage Mater*. **64**, 103059 (2024).
8. Wang, F. et al. Mitigating the interfacial concentration gradient by negatively charged quantum dots toward dendrite-free Zn anodes. *Energy Storage Mater*. **58**, 215-221 (2023).
9. Yin, J. et al. Unveiling the multifunctional regulation effect of a glutamine additive for highly reversible Zn metal anodes. *J. Mater. Chem. A.* **12**, 1543-1550 (2024).
10. Gou, Q. et al. Electrolyte regulation of bio-inspired zincophilic additive toward high-performance dendrite-free aqueous zinc-ion batteries. *Small* **19**, No. e2207502 (2023).
