# Supplementary material for: Non-solvating additives regulate zinc deposition through anion-gated Stern-layer competition
Source: Nat Commun. 2026 Aug 13;17:8260. doi: 10.1038/s41467-026-76659-1 (PMC13473634; doi:10.1038/s41467-026-76659-1)
Supplement: Supplementary file 2 [file 41467_2026_76659_MOESM2_ESM.pdf]

Description of Additional Supplementary Files

Supplementary Data 1. Molecular dynamics (MD) simulations of ZS+TEAHS, ZS+TEABF, ZS, ZS+TEABr, and ZS+TEAAc electrolytes.

Supplementary Data 2. Optimized atomic structure of Ac, BF_4, Br, H_2O, SO_4, TEA, and Zn adsorbed on Zn (002) from density functional theory (DFT) calculations.

Supplementary Data 3. Molecular dynamics (MD) simulations of interfacial distribution in the ZS+TEAHS and ZS+TEABF electrolytes.

Supplementary Data 4. ab initio molecular dynamics (AIMD) simulations of TEA on Zn (002).

Supplementary Data 5. Optimized atomic structure of SEI derived from ZS+TEAHS and ZS+TEABF electrolytes from density functional theory (DFT) calculations.
