# Supplementary material for: Non-solvating additives regulate zinc deposition through anion-gated Stern-layer competition
Source: Nat Commun. 2026 Aug 13;17:8260. doi: 10.1038/s41467-026-76659-1 (PMC13473634; doi:10.1038/s41467-026-76659-1)
Supplement: Supplementary file 3 [file 41467_2026_76659_MOESM4_ESM.pdf]

nature portfolio

Peer Review File

Non-Solvating Additives Regulate Zinc Deposition through Anion-Gated Stern-Layer Competition

Corresponding Author: Dr Hang Yang

This file contains all reviewer reports in order by version, followed by all author rebuttals in order by version.

A version of this paper was originally rejected for publication by Nature Communications, however that decision was reconsidered after appeal by the authors.

Version 1:

Reviewer comments:

Reviewer #1

(Remarks to the Author)
The authors reported an anion-released interfacial engineering strategy by replacing high-affinity SO42−, Ac−, and Br− anions with the surface-avoiding BF4−. This strategy enriches the distribution of TEA+ within the Stern layer and enables its active incorporation into the SEI. Spectroscopic and MD simulations suggest that the significant electrochemical differences stem from competitive cation/anion assembly within the EDL. Meanwhile, the rearranged interfacial structure suppressed parasitic reactions and yielded an inorganic–organic hybrid SEI with superior ion transport. Combined in-situ spectra and theoretical calculations results reveals the key kinetic processes in Zn deposition, which indicates that fast Zn2+ transport mechanistic within the SEI and moderated desolvation at the interface underpin the dendrite-free and highly reversible cycling. This study offers new insights into the interfacial function of non-solvating additives for high-performance AZIBs. Therefore, I recommend publication of this work providing that the authors appropriately addressing the comments listed below.
1: While the present work builds upon the established understanding of TMA+-induced H-bond disruption and TEA+-mediated dynamical Janus interfaces, novelty points need to be stated more clearly in introduction. Also, an optimized schematic detailing the process of trace additive incorporation and the competition between TEA+ and different anions would clarify the assembly mechanism and strengthen the manuscript.
2: In the description of overpotentials and exchange current densities for Zn//Zn cells in Figures 1a-b, the authors attribute the comparative trends observed when adding TEABF and other TEA+-based additives versus blank electrolyte to the differing effects of various anions on TEA+-mediated Zn2+ deposition. However, this interpretation appears to be inconsistent with the data in Figure 2a, which shows that Br− and Ac− ions also exhibit alienation from the Zn anode, like BF4−. The discrepancy requires a coherent explanation.
3: The authors need to provide an explanation for the sharpening and slight downfield shift of the 2H-NMR peak for water in the ZS+TEABF electrolyte (Figure 1e). Additionally, the decrease in the Raman signal intensity of O-H bond from ZS+TEABF (Figure 1f) also requires further clarification.
4: In the study of interphase Kinetics, the authors mention that the organic components of the SEI plays a crucial role in facilitating Zn2+ mobility. However, they do not specify the exact composition—is it TEA+ or its decomposition products? Additional analysis is recommended to determine the organic compositions, as this is critical for validating the potential mechanisms that accelerate Zn2+ transport.
5: In lines 248-253 and Figure S10, further validation of the competitive ion distribution mechanism was provided by a control zeta potential experiment, in which ZnSO4 was partially replaced by Zn(BF4)2. However, for logical alignment, the relevant paragraph would more closely with the descriptions in Figure 2b, and adjustments are recommended.
6: The observed shifts in the C-C/C-H peaks across different electrolytes in Figure S13 suggest that charge correction may not have been consistently applied during XPS peak fitting. This calls the accuracy of the entire analysis into question. To ensure validity, the peak fitting in Figures 4a-d and S13 must be carefully reviewed and corrected. For instance: a) The S-O/C-O and C=O/–OH signals in the O 1s spectra should not be assigned to identical binding energies. b) The ZnO peak positions must be aligned among various O 1s and Zn 2p XPS depth profiles.

Reviewer #2

(Remarks to the Author)
Comments:

The authors present an anion-released interfacial engineering strategy for aqueous Zn-ion batteries, where BF4- replacement enriches TEA+ in the Stern layer, forming a robust inorganic-organic SEI. In situ spectroscopy and simulations show this interface suppresses side reactions, couples slower desolvation with faster Zn2+ transport, and enables dendrite-free, highly reversible Zn plating, improving cycling stability and providing insights into ion-specific interfacial design. However, the concept of interfacial additives has been published a lot in this field. Especially this work does not show every compelling electrochemical performance.
In addition, I have several suggestions and clarifications that would strengthen the manuscript:
1. Be careful and serious about the font you selected for your Fig or Schematic. This is for publication in NC not for fiction.
2. In the introduction, several abbreviations (e.g., EDL, DRT, EEL, etc.) are used without definition. Please ensure all terminology is clearly introduced when first mentioned.
3. TEA+ should be introduced earlier in line 81, rather than explaining later in line 104.
4. Figure captions should be more descriptive and consistent. The use of brackets and formatting across subtitles should be standardized.
5. Line 114-116: SEM alone cannot confirm zinc sulfate by-products (ZSH). It provides only morphological information. Techniques such as XRD, EDS, or XPS should be used for chemical identification.
6. Please clarify the meaning of the yellow dashed line in Supplementary Fig. 1.
7. In Supplementary Fig. 2, specify whether the ionic conductivities were obtained experimentally or through MD simulations. It would be ideal if the authors could verify and compare the ionic conductivities and diffusion coefficients of the electrolytes between experimental measurements and MD simulations.
8. Clarify the difference between the ZS+TEAHS data shown in Fig. 1a and Supplementary Fig. 3.
9. A brief introduction should be added before line 166 to guide the reader into the next section.
10. In Fig. 2b, confirm whether the label should be “ZS+TEAHS” or “ZA+TEAHS.”
11. In Fig. 2c, please justify the use of Na2SO4-based electrolytes.
12. In Fig. 2d, please indicate the scan rate and potential range used for cyclic voltammetry.
13. Fig. 3f is not referenced in the text. Please ensure it is properly discussed and includes the time scale on the y-axis.
14. Supplementary Fig. 7, please provide the equivalent circuit model used to fit the EIS spectra and specify the frequency range. Please explain why the temperature range of 30-70 °C was chosen instead of 0-50 °C.
15. Fig. 5g, R2 value should be included.
16. Define all chemical abbreviations (e.g., EDOT, PEDOT, PVDF, NMP, CNT, etc.) when first introduced.
17. Include the source and purity of all chemicals used in the experiments.
18. The material characterization section should be expanded with sufficient details for the work to be reproduced.
19. The procedure for HRTEM and FFT indexing should be included in the material characterization section.
20. Experimental details for AFM, DRT, and EIS measurements should also be included in the Methods section.

Reviewer #3

(Remarks to the Author)
Thank you for sharing interesting work. I have several recommendations to validate the methods you used to support the claim on mechanisms.

1. The authors need to identify the surface observed in the experiment, at the moment the justification of adsorbate interaction with the Zn 002 surface is not provided. Different additives used in the battery set-up can lead to preferential formation of 002, 101, or 100 surfaces. Without clear experimental justification, the trends observed from experiment cannot be independently verified.
2. The authors must clearly mention whether they calculate neutral adsorbate or charged adsorbate on the Zn surface. It appears to me that the calculations were done in neutral adsorbate, so the discussion must be revised accordingly, by not explicitly mention anions, but rather neutral adsorbate. But, if its not the case, the authors need to better explain how they calculate the anion adsorption on the surface.
3. The authors conducted very nice FFMD simulations both in the electrolyte and at the interface. However, they use different methods for both simulations. Can the authors better explain the reason for using different methods, especially the use of Forcite Module? Can the authors give justification on how their models consistent with experiment results? Probably by comparing the density, component analysis (solvation structure), and so on?
4. Regarding the validation of electrochemistry kinetics, did the authors able to verify whether the electrolyte components are stable on the surface? Are you able to check, probably through the CV or any surface characterisation? Although the systems are simplified, but the use of such model if not verified can lead to poor justification of mechanisms.
5. The authors can also better support the SEI model using longer FFMD simulations, at the moment almost all FFMD simulations were conducted in a very short time. Probably using AIMD is a more appropriate method, as the AIMD will allow the review of molecular stability on the surface, due to the bond-breaking/forming mechanisms are captured. Nonetheless, if the FFMD are used, the better model of SEI on the surface can be achieved through longer simulation time at the interface and the results can be used to understand the migration barrier of Zn transport, statistically. This will give you better estimation in SEI from ZS-TEAHS and ZS-TEABF, rather than using controlled migration using DFT, which are limited and even the model of SEI is unrealistic.

Overall, the authors show a promising strategy to boost the performance of Zn batteries, however, the clear rationale on the

mechanisms cannot be fully explained. The justification taken from the simulations, both FFMD and DFT, are not really backed up from the observations on experiments, and the models used are too simple. The authors need either stronger experiments or a more refined models for the simulations.

Reviewer #4

(Remarks to the Author)
I co-reviewed this manuscript with one of the reviewers who provided the listed reports. This is part of the Nature Communications initiative to facilitate training in peer review and to provide appropriate recognition for Early Career Researchers who co-review manuscripts.

Reviewer #5

(Remarks to the Author)
I co-reviewed this manuscript with one of the reviewers who provided the listed reports. This is part of the Nature Communications initiative to facilitate training in peer review and to provide appropriate recognition for Early Career Researchers who co-review manuscripts.

Version 2:

Reviewer comments:

Reviewer #1

(Remarks to the Author)
After checking this revised manuscript, I can see that the authors have well addressed referees' comments and largely improved the quality of the paper: 1, It's much more clear for the presence of the novelty of non-solvation additives, with discussion on a broad context to stand out the proposed strategy herein. 2, More control tests along with characterization and modeling results are supplemented to make interface analysis (particularly EDL) and mechanistic explanation convincing. 3, Demonstration of full cells are provided to show the considerable applicability of TEAHS and TEABF electrolyte additives. 4, More experimental and simulation details are described to enrich the information. Overall, I admire the authors' sincere efforts to strength this piece of elegant work. I thus recommend publication of the updated version as it is.

Reviewer #2

(Remarks to the Author)
thanks for the revision. it is ok to accept

Reviewer #3

(Remarks to the Author)
Thanks for addressing the previous comments.
I really appreciate your response on the issues on clarifying mechanisms.

I would like further clarification on the additional AIMD simulations conducted. What software are you using? Any details on the set-up? Can the authors provide details how the structure is developed, and at what temperature the simulation was conducted?

It is important because the stability of your adsorbate can be highly influenced either by simulation time or temperatures. If the authors are limited with computational resources, increasing simulation temperature may be able to capture faster kinetics. At the moment, simulations conducted for 1 ps (very short) compared to other studies with longer simulation time up to 3-5 ps, and no information on temperature. Also, please explain the set-up of supercell, how many layers of Zn, and how many waters, and why you introduce certain configuration of TEA. Please carefully address this.

Reviewer #4

(Remarks to the Author)
I co-reviewed this manuscript with one of the reviewers who provided the listed reports. This is part of the Nature Communications initiative to facilitate training in peer review and to provide appropriate recognition for Early Career Researchers who co-review manuscripts.

Reviewer #5

(Remarks to the Author)
I co-reviewed this manuscript with one of the reviewers who provided the listed reports. This is part of the Nature Communications initiative to facilitate training in peer review and to provide appropriate recognition for Early Career

Researchers who co-review manuscripts.

Version 3:

Reviewer comments:

Reviewer #1

(Remarks to the Author)
I have gone through the revised manuscript and compared it with the previous version. Measuring the capacitance data under rigorously controlled pH conditions is necessary to yield reliable result for fair comparison and reasonable conclusion. Thus, in my opinion, the EDL analysis and related statement in this updated version is more convincing. I am in favor of this further clarification, and therfore the publication of this revised manuscript.

Reviewer #3

(Remarks to the Author)
Thank you for addressing the previous question. With the extended simulation time to 4.5 ps, at least the trend is still consistent. Despite the limitation of simulation condition and time, your work have given some preliminary indication of the stability of TEA, which can be complementary of other characterisations.

Open Access This Peer Review File is licensed under a Creative Commons Attribution 4.0 International License, which permits use, sharing, adaptation, distribution and reproduction in any medium or format, as long as you give appropriate credit to the original author(s) and the source, provide a link to the Creative Commons license, and indicate if changes were made.
In cases where reviewers are anonymous, credit should be given to 'Anonymous Referee' and the source.
The images or other third party material in this Peer Review File are included in the article’s Creative Commons license, unless indicated otherwise in a credit line to the material. If material is not included in the article’s Creative Commons license and your intended use is not permitted by statutory regulation or exceeds the permitted use, you will need to obtain permission directly from the copyright holder.
To view a copy of this license, visit https://creativecommons.org/licenses/by/4.0/

Point-by-point response

Reviewer #1:

The authors reported an anion-released interfacial engineering strategy by replacing high-affinity SO_4^{2-}, Ac^-, and Br^- anions with the surface-avoiding BF_4^-. This strategy enriches the distribution of TEA^+ within the Stern layer and enables its active incorporation into the SEI. Spectroscopic and MD simulations suggest that the significant electrochemical differences stem from competitive cation/anion assembly within the EDL. Meanwhile, the rearranged interfacial structure suppressed parasitic reactions and yielded an inorganic–organic hybrid SEI with superior ion transport. Combined in-situ spectra and theoretical calculations results reveals the key kinetic processes in Zn deposition, which indicates that fast Zn^{2+} transport mechanistic within the SEI and moderated desolvation at the interface underpin the dendrite-free and highly reversible cycling. This study offers new insights into the interfacial function of non-solvating additives for high-performance AZIBs. Therefore, I recommend publication of this work providing that the authors appropriately addressing the comments listed below.

Response:

We sincerely express our gratitude for the reviewers' recognition of our research work and their invaluable suggestions. In response to the questions posed by the reviewers, we have further verified the organic components in the SEI formed in the ZS+TEABF electrolyte through the supplementation of necessary ab initio molecular dynamics (AIMD) simulations and solid-state NMR experiments. Additionally, we have re-examined or analyzed some of the previous experimental data. We earnestly hope that the revised manuscript (R-MS) will comprehensively address all the concerns put forward by the reviewers. The detailed responses to each point of opinion are presented below, listed in blue font. All revisions in the R-MS and revised supplementary information (R-SI) have been highlighted in yellow for ease of review.

1: While the present work builds upon the established understanding of TMA^+-induced H-bond disruption and TEA^+-mediated dynamical Janus interfaces, novelty points need to be stated more clearly in introduction. Also, an optimized schematic detailing the process of trace additive incorporation and the competition between TEA^+ and different anions would clarify the assembly mechanism and strengthen the manuscript.

Response:

We sincerely appreciate your valuable suggestions, which have significantly contributed to enhancing the academic expression and readability of our manuscript. In accordance with your opinions, we have implemented targeted revisions to the introduction section, accentuating the core innovation of this research: specifically, the proposition of a novel regulatory mechanism that diverges from the traditional solvation

theory. This mechanism clearly underscores the competitive occupation behavior of functional cation in the additives and the counter-anion in the Stern layer, which serves as the primary cause for the notable disparities in their electrochemical regulatory effects. Moreover, we have systematically optimized the Scheme 1 (**Fig. 1 in R-MS**). While preserving the core characteristic of the non-solvation effect of the trace additives in this study, we have incorporated a schematic diagram to visually illustrate the competitive arrangement mechanism of the TEA^+ and cations in the Stern layer. Through a concrete presentation, we have highlighted the competitive edge of TEA^+ over weakly surface-adsorbed anions, further emphasizing their regulatory efficacy in the subsequent electrochemical process. Below are the corresponding modifications and updated Scheme 1 in the R-MS.

R-MS, Page 3: '*In recent years, electrolyte engineering has focused predominantly on modulating the Zn^{2+} solvation structure to improve anode stability. Highly concentrated salts[7], water-in-salt systems[8,9], asymmetric anion clusters[10], and various organic/ionic-liquid cosolvents[11,12] have been shown to reduce water activity, strengthen anion coordination, or rearrange the primary solvation shell (Fig. 1a). These solvation-based strategies have undeniably advanced the field and are now widely used to rationalize enhancements in Coulombic efficiency (CE), dendrite suppression, and SEI formation. Yet, a critical blind spot remains. Not all performance improvements originate from solvation restructuring. Many electrolyte additives are employed at concentrations far below the threshold required to modify the Zn^{2+} solvation shell or the bulk hydrogen-bond network, but still show pronounced effects on interfacial morphology and long-term cycling[13,14]. The mechanistic origin of such improvements is often interpreted, even sometimes implicitly, under the solvation framework, despite lacking direct evidence of any solvation change. This reveals a gap in current understanding: the field lacks a conceptual basis for interpreting the behavior of non-solvating additives, i.e., species that do not enter the solvation shell yet exert a strong influence on interfacial behavior. Here we establish a mechanism that fundamentally departs from the solvation-centric view. We show that trace-level, non-solvating electrolyte additives can regulate Zn deposition not by altering bulk solvation, but by selectively accessing the Stern layer and participating in interfacial assembly.*

Using a family of quaternary amine salts containing tetraethylamine cation and different anions (TEA^+X^-, where $X^- = BF_4^-$, HSO_4^-, CH_3COO^-, and Br^-), we demonstrate that at 10 mM, the Zn^{2+} solvation shell, water structure, and bulk electrolyte speciation remain unchanged, as confirmed by molecular dynamics (MD) simulations and diverse spectral detection. Instead, whether an additive can influence Zn deposition is determined by an entirely different factor: the competitive occupation of the Stern layer between the functional cation and its counter-anion. Different anions possess distinct intrinsic affinities toward the zinc electrode. By substituting the strongly-adsorbing anions with weakly surface-oriented anions, the distribution density of TEA^+ in the Stern layer was effectively enhanced, thus facilitating the formation of a high-quality SEI rich in TEA^+ (Fig. 1b). Moreover, based on in-situ distribution of

relaxation times (DRT) analysis, we elucidated the distinct roles of various SEI components and electric double layer (EDL) structure in affecting the zinc deposition behavior.'

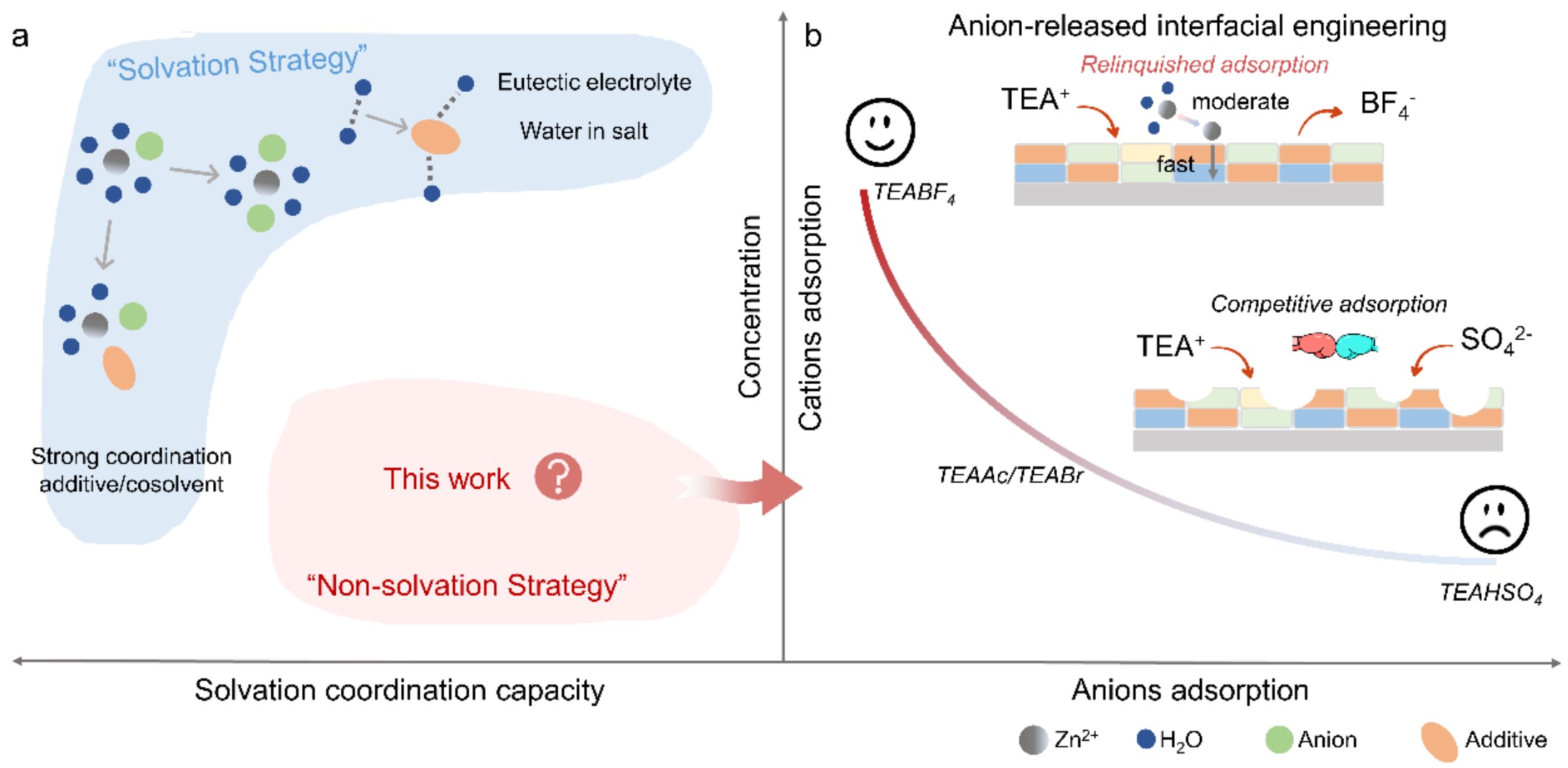

Fig. 1 | The mechanism diagram illustrating the synergistic modulation effect of cation and anions in the non-solvation additives employed in this work.

2: In the description of overpotentials and exchange current densities for Zn//Zn cells in Figures 1a-b, the authors attribute the comparative trends observed when adding TEABF and other TEA^+-based additives versus blank electrolyte to the differing effects of various anions on TEA^+-mediated Zn^{2+} deposition. However, this interpretation appears to be inconsistent with the data in Figure 2a, which shows that Br^- and Ac^- ions also exhibit alienation from the Zn anode, like BF_4^-. The discrepancy requires a coherent explanation.

Response:

Thank you for your valuable suggestions, which have significantly contributed to understanding the competitive distribution of anions and cations of the additives within the Stern layer. In response to this, the following is our explanation:

(I) Comparison among TEA^+-containing electrolytes (constant additive concentration). As depicted in Fig. 1a-b (**Fig. 2a-b in R-MS**), the electrochemical kinetics (measured by deposition overpotential and exchange current density) of this series of electrolytes demonstrated a high level of consistency with the adsorption energy data in Fig. 2a (**Fig. 3a in R-MS**). Specifically, BF_4 exhibited the weakest tendency for adsorption on the zinc surface, enabling the electrode/electrolyte interface (EEL) region to accumulate a greater amount of TEA^+, which consequently led to the slowest zinc deposition kinetics in the ZS+TEABF electrolyte. In contrast, Br and Ac had a moderate competitive adsorption strength at the interface active sites, and thus their deposition kinetics rate was between that of the ZS+TEAHS electrolyte (the fastest) and the ZS+TEABF electrolyte (the slowest). In conclusion, the electrochemical kinetic results obtained

experimentally in this study (**Fig. 2**) were reasonably corroborated by the theoretical calculation (**Fig. 3a**).

(II) Why do the ZS+TEABr and ZS+TEAAc electrolytes demonstrate opposite kinetic transformation compared to that of ZS+TEABF?

The reviewers noted that **Fig. 3a** suggests that Br and Ac ions also exhibit alienation from the Zn anode, like BF. However, in the ZS baseline electrolyte there is no additional 10 mM anion introduced, whereas in the ZS+TEABr and ZS+TEAAc electrolytes the total concentration of specifically adsorptive anions (SO_4^{2-}+Br^- or SO_4^{2-}+Ac^-) is increased. Furthermore, the DFT calculation results demonstrated that although the adsorption affinity of Br and Ac on the zinc surface was slightly weaker than that of SO_4, the differences in the adsorption energies among the three were extremely minor (adsorption energy values: Ac: −2.62 eV, Br: −2.78 eV, SO: −2.92 eV, BF: −1.31 eV). Therefore, adding TEABr or TEAAc effectively increases the number of strongly adsorptive anions available to occupy the interfacial region. This leads to a more compact, anion-rich Stern layer, as supported by the more negative zeta potentials (**Fig. 3b**). In this scenario, TEA^+ was significantly excluded from the Stern inner layer because of its competitive adsorption disadvantage with Br^-/Ac^-. In conclusion, the electrochemical behavior of the ZS+TEABr/TEAAc electrolyte is primarily governed by this concentration-driven anion crowding effect, which also results in a slight enhancement of its kinetic characteristics compared to the ZS electrolyte, in contrast to the situation in the ZS+TEABF electrolyte.

3: The authors need to provide an explanation for the sharpening and slight downfield shift of the ^{2}H-NMR peak for water in the ZS+TEABF electrolyte (Figure 1e). Additionally, the decrease in the Raman signal intensity of O-H bond from ZS+TEABF (Figure 1f) also requires further clarification.

Response:

Thank you for your valuable suggestions.

Clarification regarding NMR:

We hypothesize that the notable differences in the water peak widths of these three electrolytes result from the non-simultaneous conduction of NMR measurements. To mitigate errors stemming from disparities in experimental conditions, we repeated all measurements in a single session, with identical instrument settings, lock/shim parameters， and pulse sequence selection.

The revised data (now updated in **Fig. 2e**) demonstrate that subsequent to normalization, the ^{2}H chemical shifts of ZS, ZS+TEABF, and ZS+TEAHS electrolytes are essentially identical, with a difference of less than 0.003 ppm. This discovery further corroborates our conclusion that TEA^+ assumes a non-solvent role, and its effect occurs at the EDL/Stern layer, rather than through bulk solvation or hydrogen-bond restructuring, consistent with our MD simulations and Raman analysis. Moreover, the width differences of the water peak are also indistinguishable within the instrument's

resolution range. Thus, the earlier apparent "sharpening and downfield shift" of ZS+TEABF was not intrinsic to the electrolyte but originated from inter-session instrumental drift.

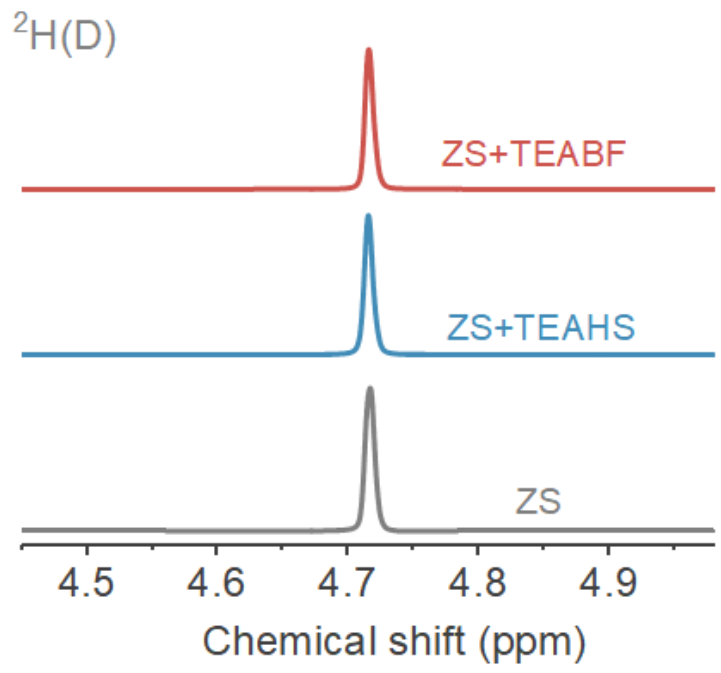

Fig. 2 | e The normalized ^{2}H NMR obtained from ZS, ZS+TEAHS, and ZS+TEABF electrolytes.

Clarification regarding Raman:

We appreciate the reviewer's question about the slightly lower O-H intensity observed for the ZS+TEABF electrolyte. We attribute this to standard local-field and scattering-volume effects that arise from changes of ionic strength and refractive index in electrolyte when $TEABF_4$ is introduced (*J. Phys. Chem. B 2003, 107, 7788–7794*). In general, the solvent environment of an electrolyte is assessed through peak shifts or relative area ratios of characteristic bands in Raman spectra. To clarify this point, we initially normalized the Raman spectra and conducted a re-analysis, as depicted in the revised **Fig. 2f**. Both the peak positions and the relative area fractions of O–H_1 (strong hydrogen bonds) and O–H_2 (weak hydrogen bonds) are essentially identical for ZS, ZS+TEABF and ZS+TEAHS (54.2%/45.8%). This demonstrates that the distribution between strongly and weakly hydrogen-bonded water species, i.e. the hydrogen-bond network topology, is not measurably altered by the addition of TEA^+-based additives at 10 mM. Therefore, the Raman data are fully consistent with our conclusion from ^{2}H-NMR and MD simulations that $TEABF_4$ behaves as a non-solvating additive and does not substantially perturb the bulk hydrogen-bond network of water.

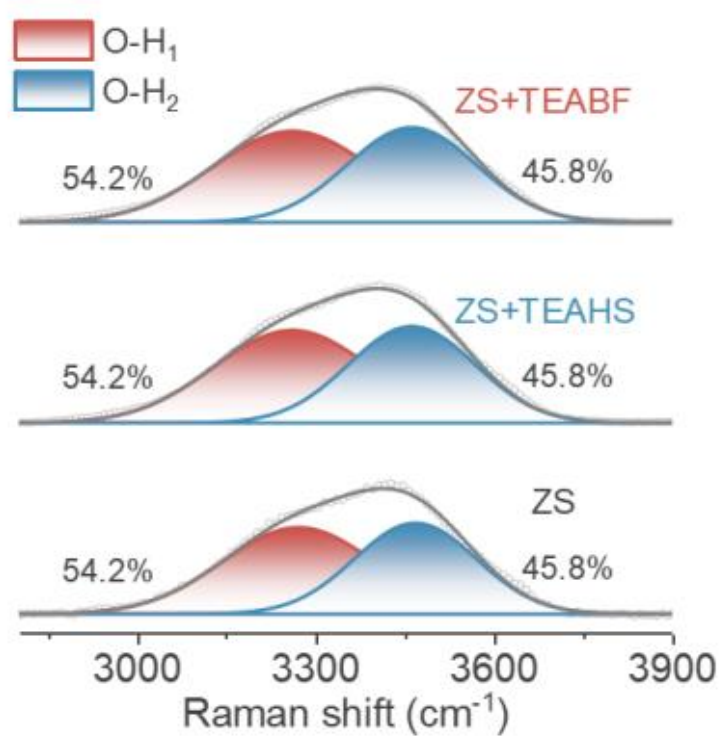

Fig. 2 | f The fitted Raman spectra of the O-H band obtained from ZS, ZS+TEAHS, and ZS+TEABF electrolytes.

4: In the study of interphase Kinetics, the authors mention that the organic components of the SEI plays a crucial role in facilitating Zn^{2+} mobility. However, they do not specify the exact composition—is it TEA^+ or its decomposition products? Additional analysis is recommended to determine the organic compositions, as this is critical for validating the potential mechanisms that accelerate Zn^{2+} transport.

Response:

Sincerely thank you for your valuable suggestions. These suggestions have significantly contributed to improving the comprehensiveness and scientific rigor of our research. Consequently, we have supplemented experimental tests and Ab Initio Molecular Dynamics (AIMD) simulations to precisely and qualitatively analyze the existence form of the organic components in the SEI. Initially, we carried out CV tests in deionized water (DIW) with and without 0.01 M $TEABF_4$ added. A three-electrode system was employed, and the scanning was conducted within a voltage window of 0–1.2 V. The results indicated that the CV curves in both liquid environments demonstrate high repeatability. Except for a minor disparity in the response current, no other redox peaks were identified, suggesting that TEA^+ exhibits excellent electrochemical stability within the actual working voltage range of aqueous batteries. Furthermore, we carried out AIMD simulations to investigate the decomposition behavior of TEA on the zinc surface in aqueous solution. Throughout the 1000 fs simulation period, none of the diverse chemical bonds in TEA (C–N, C–C, C–H) were broken, which suggests that it exhibits relatively low reactivity at the interface.

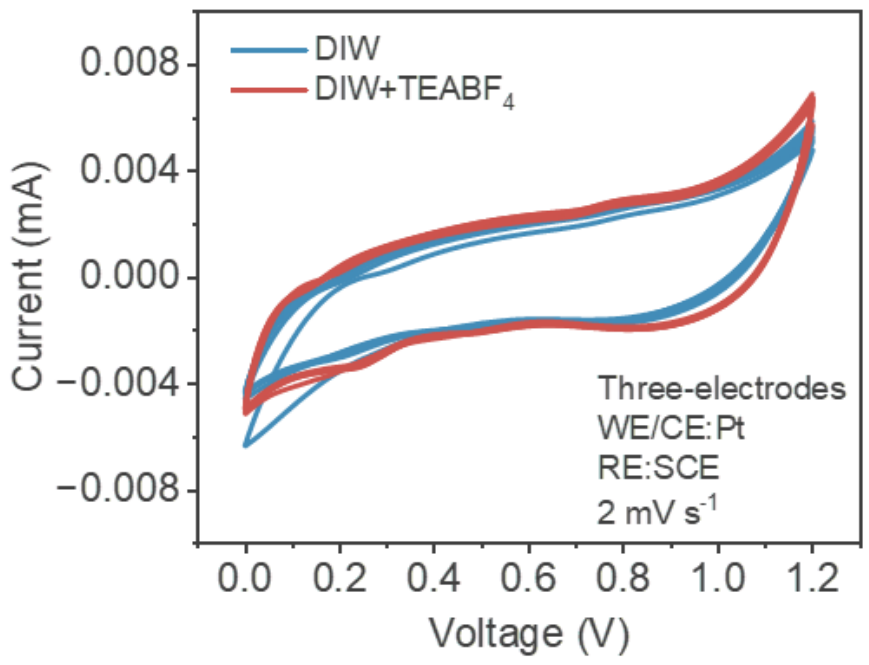

Supplementary Fig. 12. The CV curves of the DIW with or without 0.01 M $TEABF_4$ were measured within the potential range of 0–1.2 V.

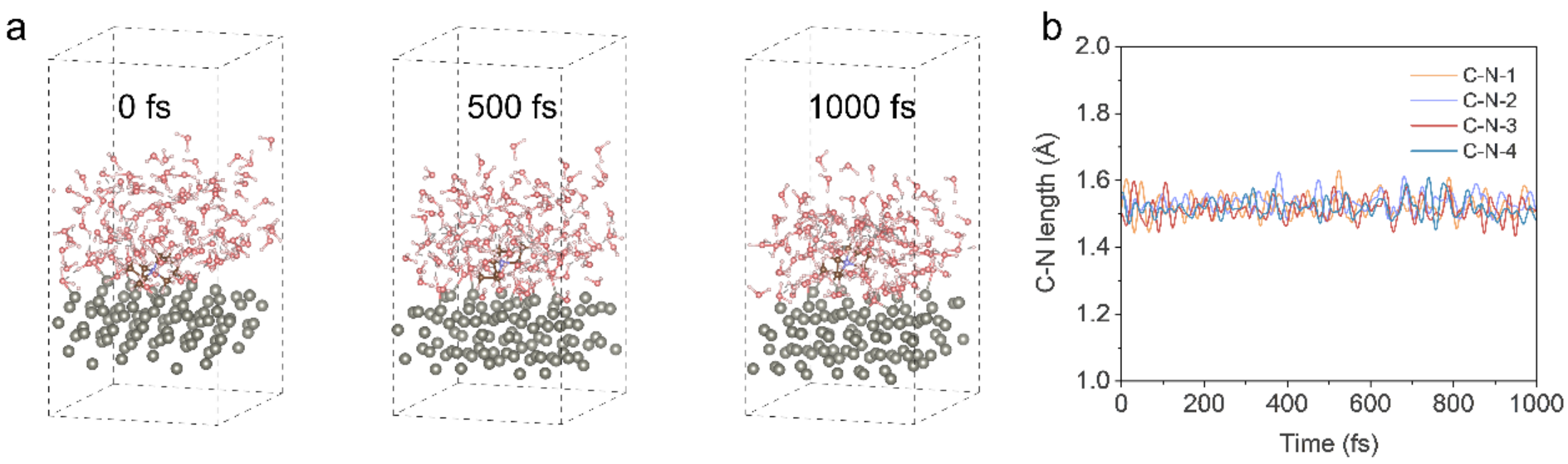

Fig. 5 | a Snapshots from AIMD simulations depicting TEA existence on the Zn surface.

b Changes in the length of C-N bond during MD simulation.

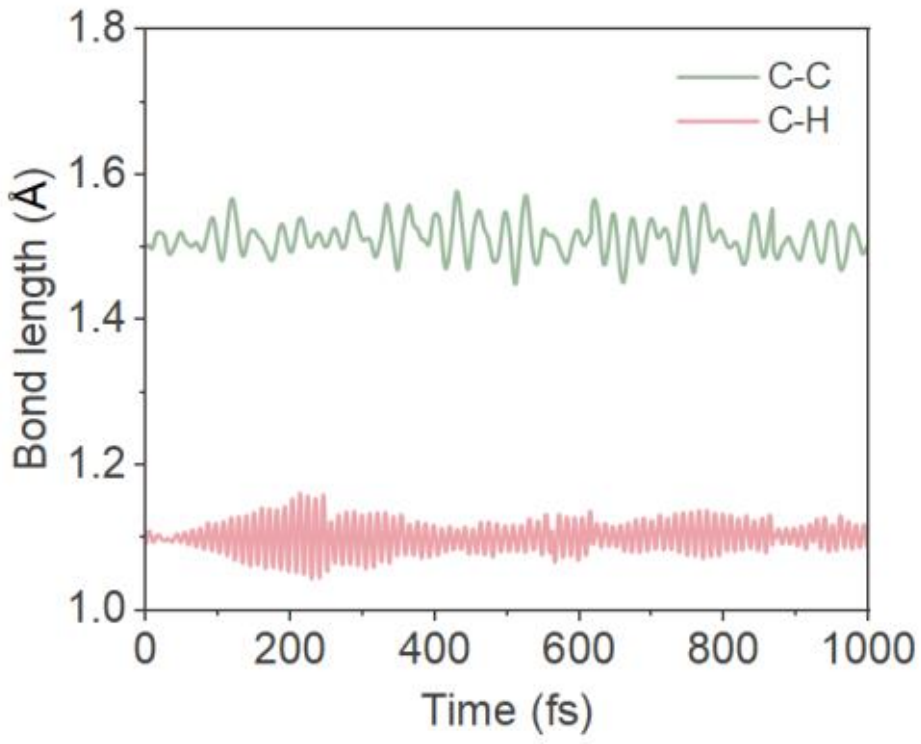

Supplementary Fig. 13. Changes in the C-C and C-H bond length during AIMD simulations.

What is of particular significance is that we performed a magic-angle spinning (MAS) solid-state NMR analysis on the surface film of cycled zinc electrode. The ^{13}C spectrum of the SEI sample exhibited only two characteristic peaks that were in line with the $TEABF_4$ powder: one at 50.9 ppm (corresponding to N^+-CH_2) and the other at 9.8 ppm (corresponding to -CH_3) (*Chem. Mater. 2000, 12, 3052-3059*). This result strongly corroborated the presence of TEA^+ in its unfragmented state within the SEI.

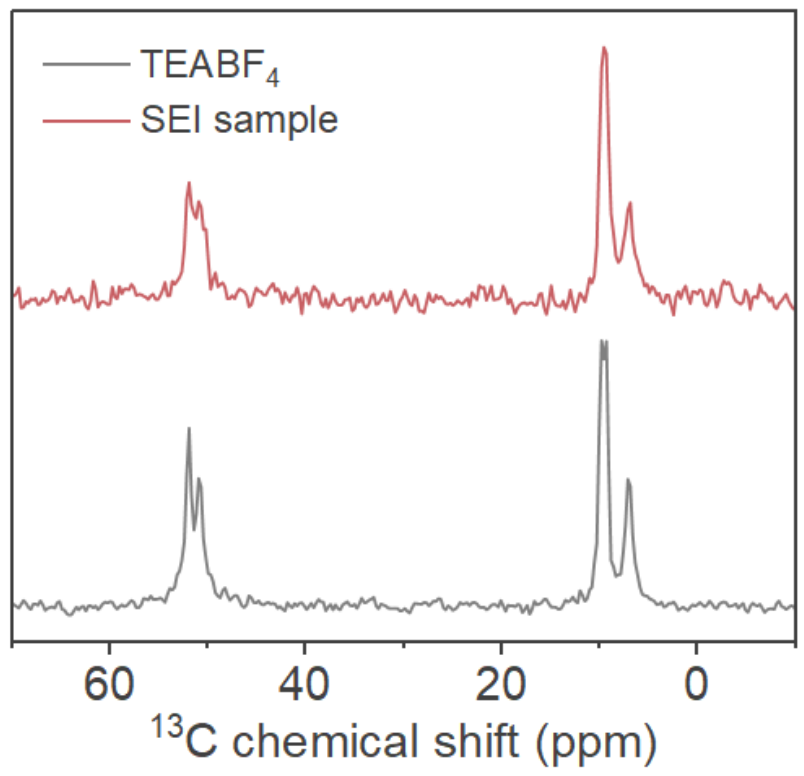

Supplementary Fig. 14. The ^{13}C NMR spectra of SEI sample derived from cycled zinc electrode and $TEABF_4$ powder. The zinc electrode was retrieved from Zn||Zn symmetric cell after 10 cycles under the condition of 5 mA cm^{-2}@2.5 mAh cm^{-2}.

R-MS, Page 11: '*Before this, we initially assessed the potential decomposition behavior of TEA^+ on the electrode surface and its possible contribution to the formation of the SEI. Cyclic voltammetry (CV) tests were initially conducted within a wide voltage window (0–2.0 V) using deionized water (DIW) and DIW containing 0.01 M $TEABF_4$ in a three-electrode system to simulate the actual working voltage environment of the battery (Supplementary Fig. 12). The results indicated that no other distinct redox signals were detected in the latter system, suggesting that $TEABF_4$ exhibits good electrochemical stability in aqueous electrolytes. To further examine the dynamic behavior of TEA^+ at the zinc electrode, ab initio molecular dynamics (AIMD) simulations were performed, with a focus on the real-time changes in the bond lengths of TEA+ in aqueous solutions (Fig. 5a). The simulation results demonstrated that within 1000 fs simulation time, the C–N, C–C, and C–H bonds remained intact, indicating that TEA^+ is structurally stable in aqueous electrolytes (Fig. 5b and Supplementary Fig. 13). More significantly, the zinc surface films were collected after cycling for magic-angle spinning (MAS) solid-state NMR detection analysis. The ^{13}C spectrum presented only two main signals: the α-C (N^+–CH_2) peak at approximately 50.9 ppm and the β-C (–CH_3) peak at approximately 9.8 ppm[23], which were consistent with the spectrum of $TEABF_4$ powder (Supplementary Fig. 14). This indicates that even after long-term cycling, TEA^+ can still exist in the SEI in its original structure. Above results underscore that, in contrast to prior studies on analogous ammonium salt additives[24], TEA^+ is unlikely to undergo a decomposition reaction akin to the Hoffmann elimination in this study, owing to its high symmetry and the relatively weak acidity of the β-H.*'

5: In lines 248-253 and Figure S10, further validation of the competitive ion distribution mechanism was provided by a control zeta potential experiment, in which $ZnSO_4$ was partially replaced by $Zn(BF_4)_2$. However, for logical alignment, the relevant paragraph would more closely with the descriptions in Figure 2b, and adjustments are recommended.

Response:

We sincerely appreciate the reviewer's insightful suggestion, which indeed helped us improve the logical continuity between Fig. 2b (**Fig. 3b in R-MS**) and the supplementary zeta-potential experiments.

[redacted]

ζ

)

[redacted] $^{2-}_{4}$ and BF_4^-. To decouple these two effects and isolate the role of anion adsorption, we redesigned the control experiment. Specifically, we examined the change in zeta potential (Δζ) induced by adding 0.01 M $TEABF_4$ to a series of mixed electrolytes containing varying molar ratios of $ZnSO_4$ and $Zn(BF_4)_2$ (**R-SI, Supplementary Fig. 10**). This approach ensures that any observed Δζ originates from the relative interfacial

c

o

m

p

e

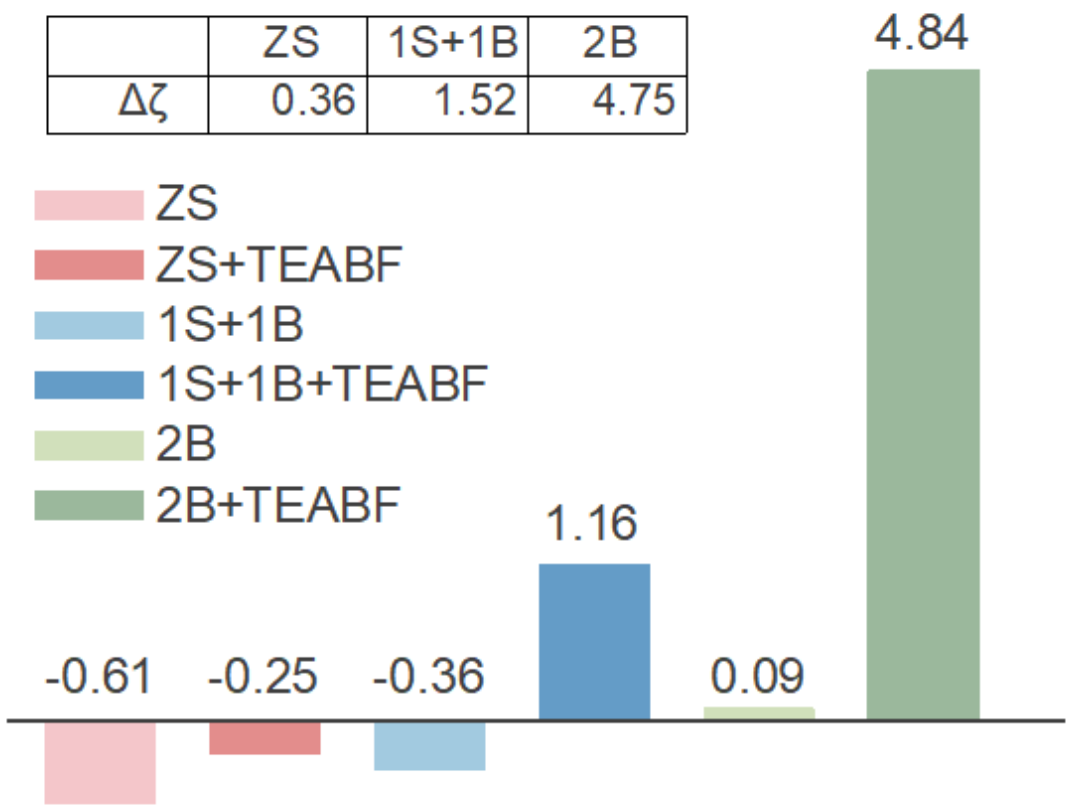

Supplementary Fig. 10. The zeta potential of Zn anode in electrolytes containing varying ratios of $Zn(BF_4)_2$ and $ZnSO_4$ ratios, measured under conditions with and without 0.01 M $TEABF_4$.

The revised data show a clear and monotonic trend: as the fraction of $Zn(BF_4)_2$ increases, the positive shift in ζ potential upon adding $TEABF_4$ becomes progressively larger. This behavior directly reflects the mechanism illustrated in **Fig. 3b**, namely, that weakly zincophilic anions such as BF_4^- occupy the Stern layer less effectively, thereby creating interfacial "vacancy space" that facilitates TEA^+ enrichment. In contrast, electrolytes dominated by strongly adsorbing SO_4^{2-} exhibit only a minimal Δζ, which is consistent with the weak competitive status of TEA^+ in the inner EDL.

Thus, the redesigned zeta-potential experiment provides direct and quantitative validation of the proposed ion competitive-distribution mechanism: the weaker anion's affinity for Zn electrode, the more effectively TEA^+ can accumulate at the interface. To improve coherence, we have incorporated the following revised description into the **R-MS, Page 7**:

R-MS, Page 7: '*This competitive ion distribution mechanism was further validated by a control experiment in which $ZnSO_4$ was partially replaced by $Zn(BF_4)_2$. With increasing proportions of BF_4, upon addition of 0.01 M $TEABF_4$, the ζ potential of all systems exhibited a positive shift, and the magnitude of this shift increased progressively with higher BF_4^- content (Supplementary Fig. 10). These results provide direct experimental evidence supporting the proposed interfacial restructuring effect driven by anion selection.*'

6: The observed shifts in the C-C/C-H peaks across different electrolytes in Figure S13 suggest that charge correction may not have been consistently applied during XPS peak fitting. This calls the accuracy of the entire analysis into question. To ensure validity, the peak fitting in Figures 4a-dand S13 must be carefully reviewed and corrected. For instance: a) The S-O/C-O and C=O/–OH signals in the O 1s spectra should not be assigned to identical binding energies. b) The ZnO peak positions must be aligned among various O 1s and Zn 2p XPS depth profiles.

Response:

Thank you very much for pointing out this important issue. We have carefully reprocessed all XPS spectra and fully corrected the charge-referencing procedure. The revised C 1s spectra for all three electrolytes are now consistently calibrated to the standard carbon peak (284.8 eV), as shown in the updated **Supplementary Fig. 16**. After applying this unified charge correction, the peak positions of C–C/C–H are aligned across all samples, ensuring the reliability of subsequent deconvolutions.

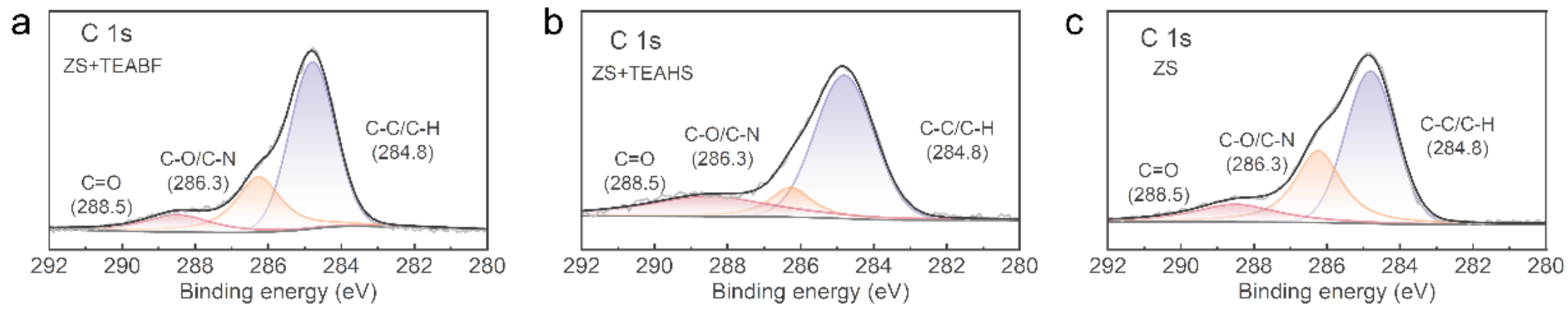

Supplementary Fig. 16. XPS spectra of C 1s for Zn electrode cycled in (a) ZS+TEABF, (b) ZS+TEAHS, and (c) ZS electrolytes.

Following recalibration, we re-examined the O 1s and Zn 2p spectra. The corrected O 1s fitting now clearly distinguishes all the components contributions. The same components exhibit nearly identical binding energies in different electrolytes. In addition, the ZnO peak positions in the O 1s and Zn 2p depth profiles are now fully consistent among all samples, resolving the discrepancies noted in the original version. The reprocessed spectra reveal the following:

The characteristics of SEI formed in the ZS and ZS+TEAHS electrolytes:

The SEI are dominated by hydroxyl/water-derived species and sulfate-related decomposition products. A small amount of $ZnCO_3$ is present at the outermost surface but progressively diminishes with sputtering depth, eventually disappearing. These observations agree well with our HRTEM analysis, confirming that the inorganic SEI components primarily consist of basic zinc sulfate (ZSH) and ZnO.

The characteristics of SEI formed in the ZS+TEABF electrolyte:

After correction, the depth-resolved spectra clearly show the presence of $ZnCO_3$ and $ZnSO_3$, which constitute the main inorganic components in this system. Their assignments are consistent across O 1s, C 1s, and Zn 2p spectra and corroborated by the HRTEM and TOF-SIMS results.

Overall, the updated XPS analysis fully addresses the reviewer's concerns. The charge-corrected and consistently fitted spectra validate the compositional differences we report for the SEI layers in the three electrolytes.

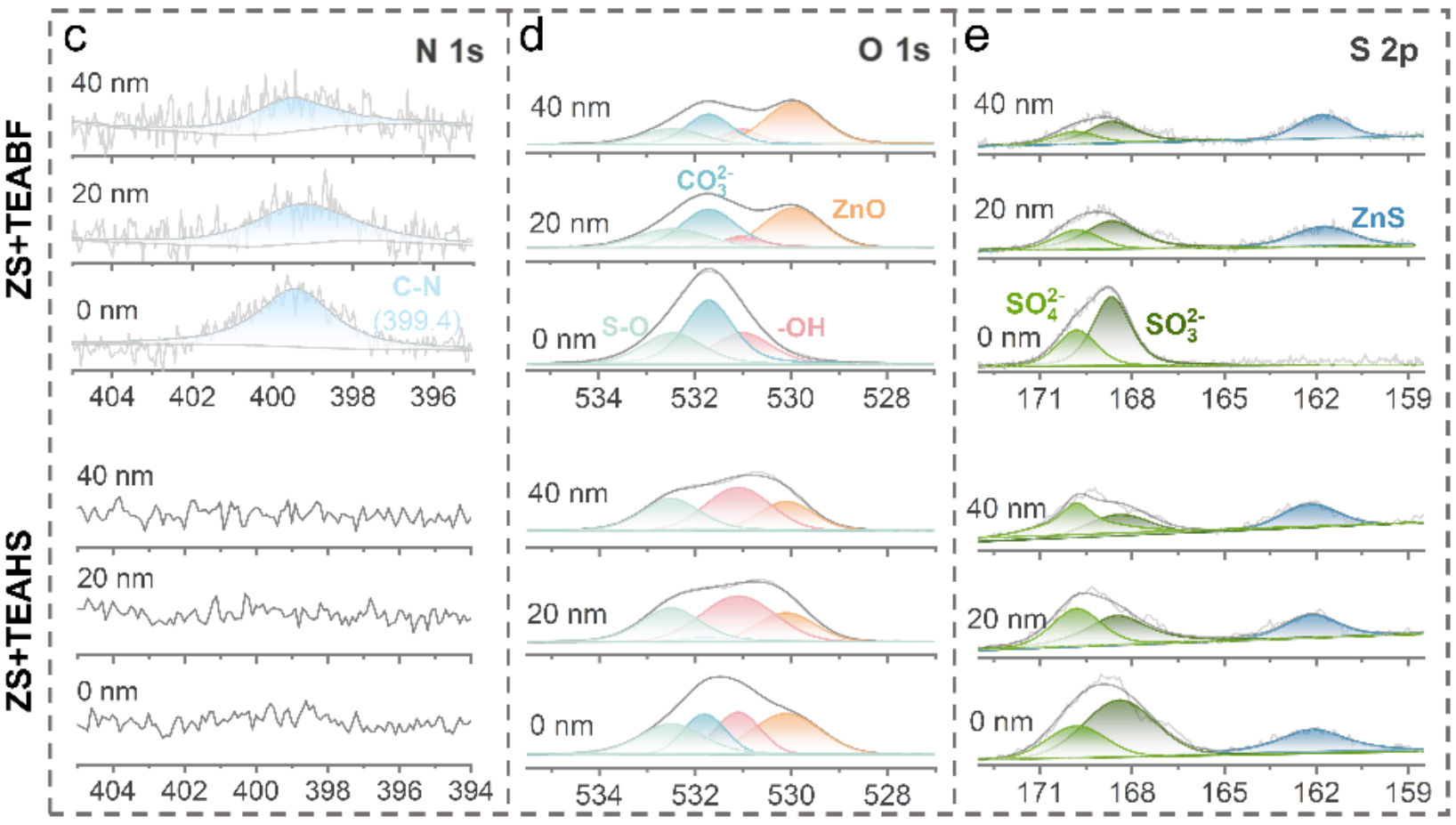

Fig. 5 | c N 1s, **d** O 1s and **e** S 2p XPS depth profiles of Zn anodes after 10 cycles in ZS+TEABF and ZS+TEAHS electrolytes.

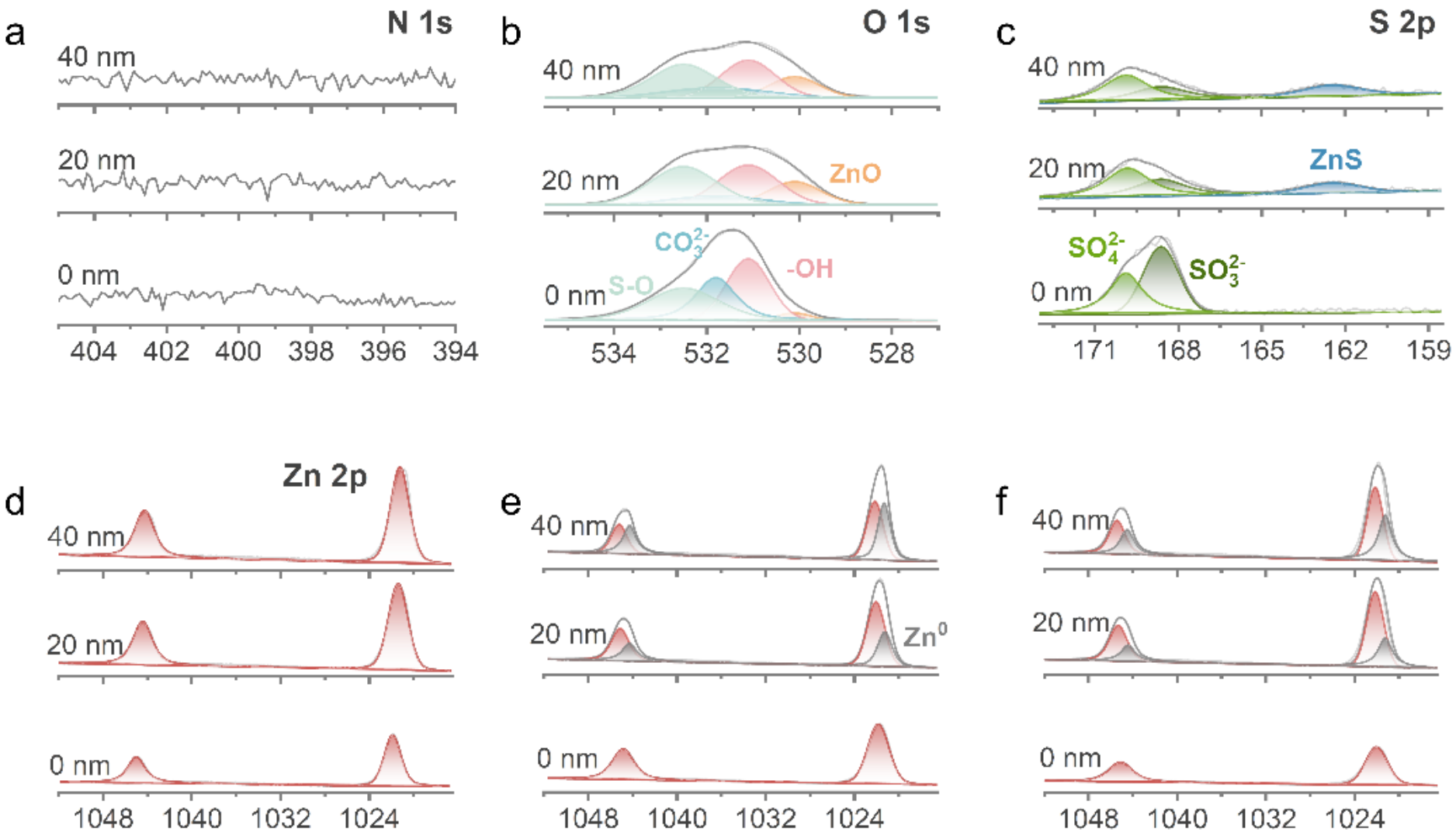

Supplementary Fig. 17. The XPS depth profile for (a) N 1s, (b) O 1s, (c) S 2p of Zn anodes after 10 cycles in ZS electrolyte. (d-f) Zn 2p of cycled Zn in (d) ZS+TEABF, (e) ZS+TEAHS and (f) ZS electrolyte.

Reviewer #2:

Comments:

The authors present an anion-released interfacial engineering strategy for aqueous Zn-ion batteries, where BF_4^- replacement enriches TEA^+ in the Stern layer, forming a robust inorganic-organic SEI. In situ spectroscopy and simulations show this interface suppresses side reactions, couples slower desolvation with faster Zn^{2+} transport, and enables dendrite-free, highly reversible Zn plating, improving cycling stability and providing insights into ion-specific interfacial design. However, the concept of interfacial additives has been published a lot in this field. Especially this work does not show every compelling electrochemical performance.
In addition, I have several suggestions and clarifications that would strengthen the manuscript:

Response:

We sincerely thank the reviewers for their thoughtful comments. With regard to the reviewers' comment that "the concept of interfacial additives has been published a lot in this field", we have carried out a comprehensive review of the recent research on trace additives in aqueous batteries. Drawing on the research papers incorporated in the Web of Science database, it is evident that the majority of current studies concentrate on the regulation of the solvation shell structure, or the optimization of the orientation arrangement of water molecules and additive molecules within the Helmholtz layer. In contrast, research on the mechanism of non-solvation additives remains scarce, and the existing relevant reports predominantly confine themselves to the formation of a single specific component in the SEI induced by the additive. Therefore, the anion–cation competitive distribution mechanism in this work has not been previously reported in the related research field.

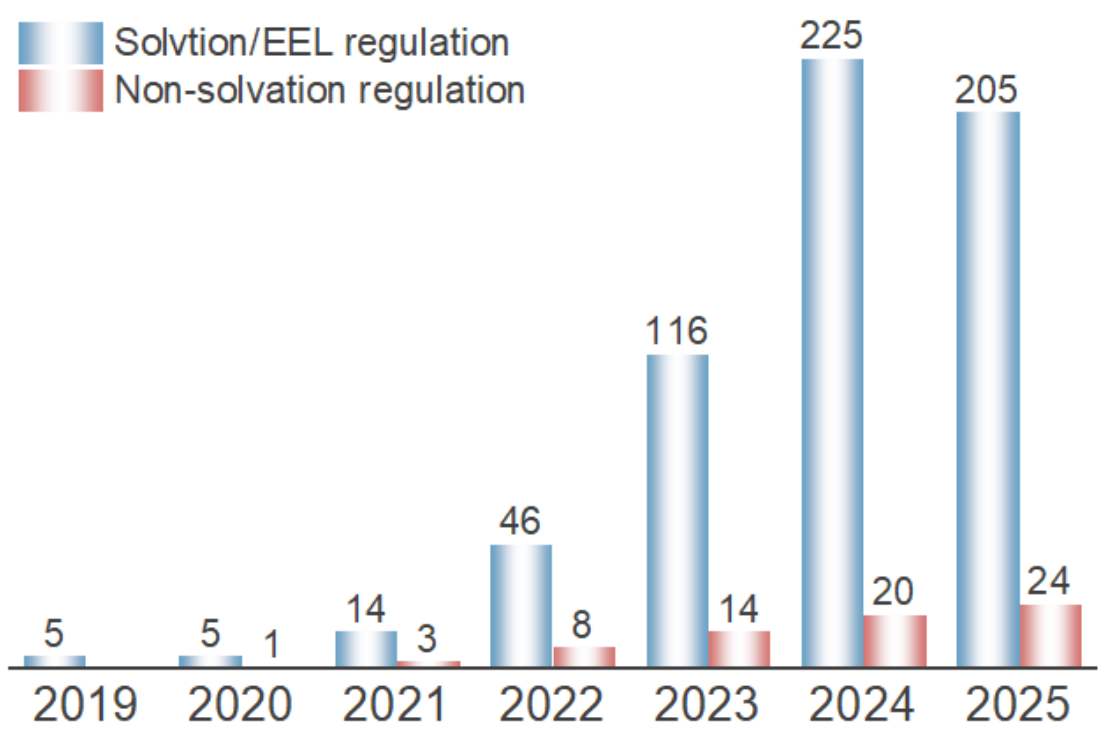

The number of publications related to additives in aqueous batteries, with the data collected from the Web of Science in December 2025.

We would like to further clarify the novelty and scientific value of this work by outlining the actual progression of our study. Our research began not from a conceptual hypothesis, but from an experimental observation: among several TEA^+-based

additives screened at trace concentrations, $TEABF_4$ consistently produced markedly improved Zn reversibility, whereas additives with the same cation but different anions (HSO_4^-, Br^-, Ac^-) did not. This immediately revealed a contradiction with existing understanding. Since the TEA^+ concentration was kept extremely low, and the fact that diverse spectra analyses of the electrolyte and molecular dynamics (MD) simulations showed no detectable changes in Zn^{2+} solvation or the bulk hydrogen-bond network, the commonly used solvation-based explanations could not account for why only $TEABF_4$ was effective. This gap highlighted a blind spot in current additive research, where additive efficacy is often attributed solely to solvation-shell modification, without considering whether the additive actually gains interfacial access.

To resolve this contradiction, we systematically examined the interfacial behavior of the TEA^+-anion pairs and discovered a previously unrecognized principle: the effectiveness of an additive is governed not by its ability to change solvation, but by whether the accompanying anion allows the functional cation to enter the Stern layer. Through interfacial MD density profiles, differential capacitance, zeta-potential mapping with $ZnSO_4/Zn(BF_4)_2$ gradient electrolytes, and in-situ Raman signatures, we revealed that strongly zincophilic anions (SO_4^{2-}, HSO_4^-, Br^-, Ac^-) crowd the Stern layer and exclude TEA^+, whereas the weakly adsorbing BF_4^- creates an "anion-released window" that enables TEA^+ accumulation at the interface. This experimentally validated phenomenon forms the basis of the anion–cation competitive distribution mechanism proposed in this work, that is a mechanism absent from prior additive literature, which has largely emphasized solvation modulation rather than interfacial accessibility.

Building on this mechanistic insight, we further established a quantitative structure–function correlation for the TEA^+-enabled hybrid SEI. Using XPS depth profiling, TOF-SIMS mapping, HRTEM, and ab initio MD, we reconstructed the full SEI architecture and demonstrated how $ZnCO_3/ZnSO_3$-rich hybrid SEI incorporating TEA^+ support faster Zn^{2+} migration and more uniform nucleation, whereas ZnO/ZSH-dominated inorganic SEI formed in the absence of TEA^+ leads to poorer kinetics and side reactions. This level of chemical–structural quantification is rarely achieved in previous studies regarding aqueous battery additives, and it directly links SEI composition to plating behavior.

Regarding the reviewer's comment that "this work does not show every compelling electrochemical performance", we emphasize that this manuscript focuses on mechanistic understanding under dilute, practically relevant electrolyte conditions, rather than device-level performance optimization. Even so, the TEA^+-riched hybrid SEI exhibits remarkable durability, maintaining zinc activity after two months of static storage, which is exceeding most state-of-the-art additive systems, and shows consistent improvements across coin cells, pouch cells, electrolytic systems, and various cathode chemistries. These results highlight the generality, robustness, and mechanistic significance of the proposed anion-regulated interfacial strategy.

Once again, we thank the reviewers for their constructive feedback, which has

greatly helped to refine the scientific clarity and presentation of this work. In the following pages, detailed responses to each of the reviewers' comments are provided in blue text. Corresponding changes in the revised manuscript (R-MS) are highlighted in yellow.

1. Be careful and serious about the font you selected for your Fig or Schematic. This is for publication in NC not for fiction.

Response:

Thank you for your valuable suggestion. We regret the oversight regarding the font selection. The primary purpose of employing the handwritten-style fonts was intended to convey a sense of approachability and visual appeal, however, we acknowledge that this choice may have compromised the professional tone of the manuscript. Accordingly, we have conducted comprehensive revisions to the font style and layout format of the schematic diagrams, and also implemented optimization of the line style and color to guarantee that all diagrams are presented with clarity, simplicity, and standardization, fully meeting the stringent editorial standards of Nature Communications. Moreover, to further enhance the scientific logic and core innovation of this research, we have redrawn Scheme 1 (**R-MS, Fig. 1**), aiming to visually and precisely illustrate the competitive arrangement mechanism of cation-anion in the non-solvation additive at the electrode/electrolyte interface, offering stronger support for the visual interpretation of the research conclusions.

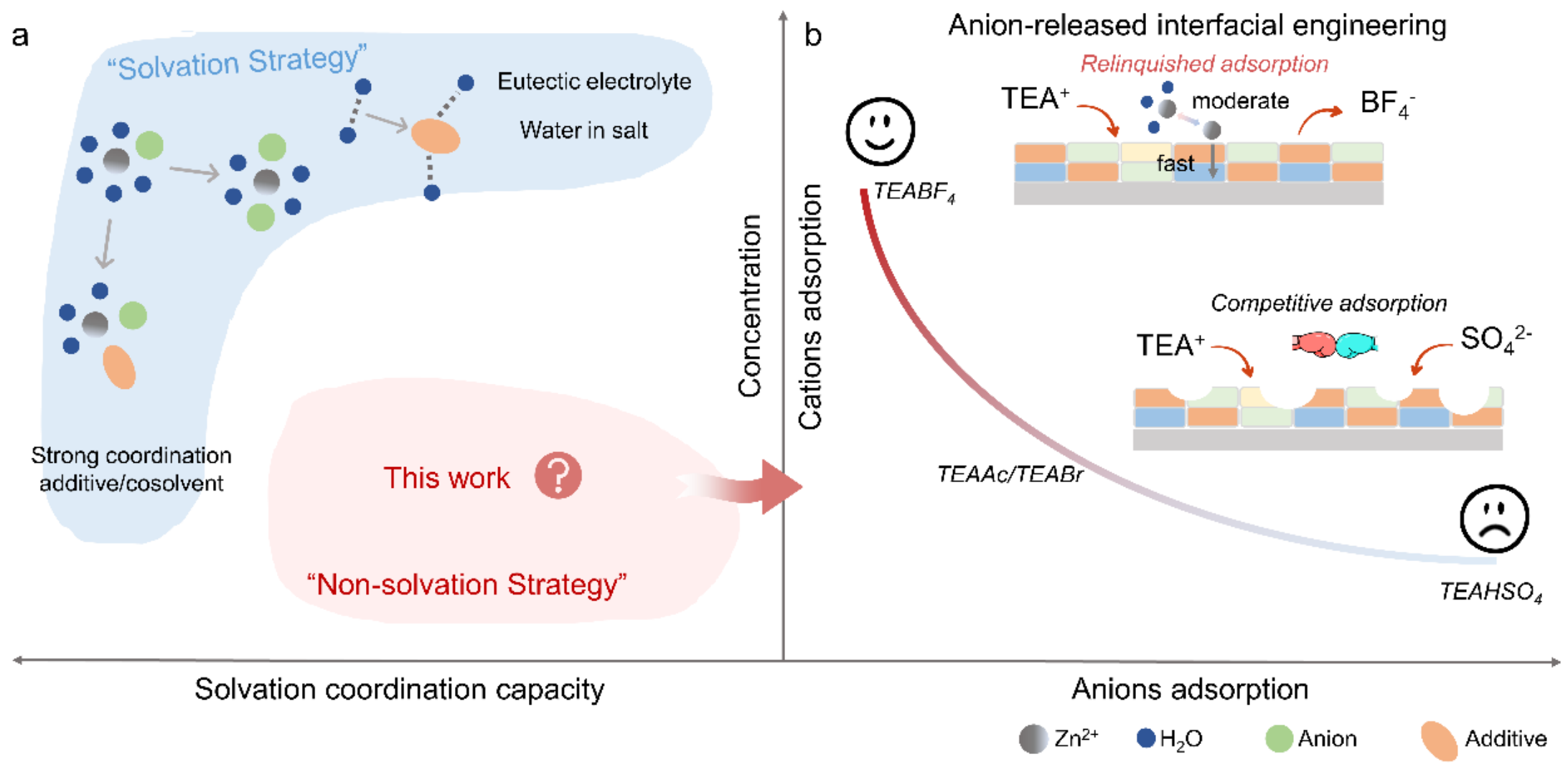

Fig. 1 | The mechanism diagram illustrating the synergistic modulation effect of cation and anions in the non-solvation additives employed in this work.

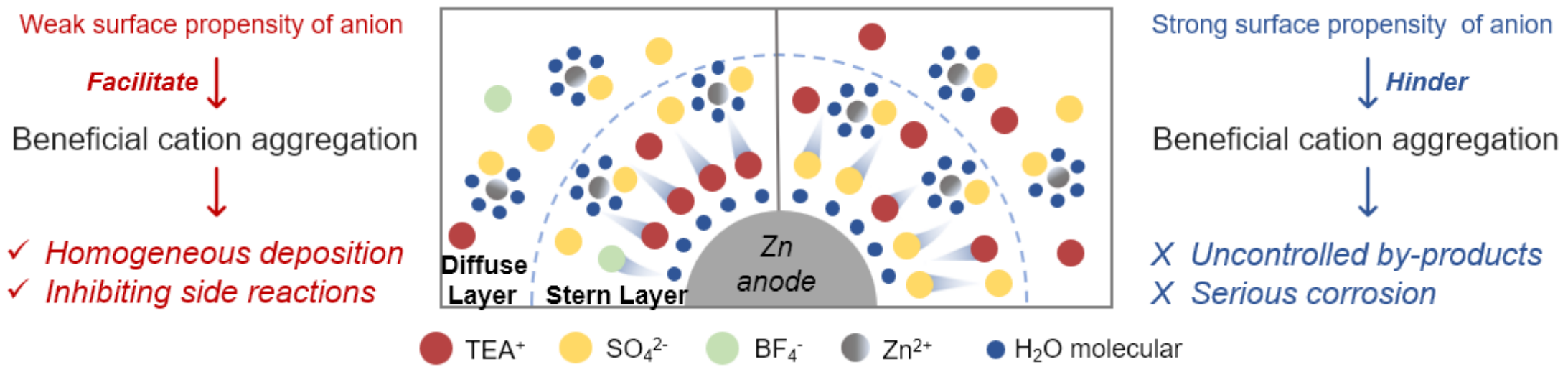

Fig. 4 | i Schematic illustration of the structural differences in molecular participation at the EEL, induced by competitive behavior between anion and cation.

2. In the introduction, several abbreviations (e.g., EDL, DRT, EEL, etc.) are used without definition. Please ensure all terminology is clearly introduced when first mentioned.

Response:

Thank you for your suggestions. The manuscript has been thoroughly revised to ensure that all abbreviations are clearly defined upon their first appearance. The following outlines the specific modifications implemented in R-MS.

R-MS, Page 4: '*Moreover, based on in-situ distribution of relaxation times (DRT) analysis, we elucidated the distinct roles of various SEI components and electric double layer (EDL) structure in affecting the zinc deposition behavior. This study establishes a clear correlation between the competition distribution behavior of additive and the evolution of the electrode-electrolyte layer (EEL) chemistry along with functional SEI formation.*'

3. TEA^+ should be introduced earlier in line 81, rather than explaining later in line 104.

Response:

Thank you for your suggestion. The full name of TEA^+ has been provided upon its first appearance in the manuscript. The revised section is presented below.

R-MS, Page 3: '*Using a family of quaternary amine salts containing tetraethylamine cation and different anions (TEA^+X^-, where $X^- = BF_4^-$, HSO_4^-, CH_3COO^-, and Br^-)…*'

4. Figure captions should be more descriptive and consistent. The use of brackets and formatting across subtitles should be standardized.

Response:

We sincerely appreciate your valuable suggestion, which has significantly contributed to enhancing the quality of our manuscript. In response, we have carefully reviewed the latest publication guidelines of NC and thoroughly revised the captions throughout the manuscript. Furthermore, we have rephrased the titles of several figures to ensure they accurately and clearly reflect the corresponding visual content. Here is the revised content in R-MS.

R-MS, Page 4: '***Fig. 1 | The mechanism diagram illustrating the synergistic modulation effect of cation and anions in the non-solvation additives employed in***

this work.'

R-MS, Page 6: '***Fig. 2 | Influence of trace TEA$^+$-based additives on Zn^{2+} solvation and electrochemical performance. a*** *Galvanostatic cycling performance of Zn||Zn symmetric cells in 10 mA cm^{-2}/ 5 mAh cm^{-2} and* ***b*** *exchange current densities of the zinc plating/stripping using different electrolytes.* ***c*** *Coordination numbers of H_2O and anions in the primary solvation shell of Zn^{2+}, and* ***d*** *corresponding MD snapshots showing electrolyte configurations in the presence of different additives.* ***e*** *The normalized ^{2}H NMR and* ***f*** *fitted Raman spectra of the O-H band obtained from ZS, ZS+TEAHS, and ZS+TEABF electrolytes.*'

R-MS, Page 10: '***Fig. 4 | Interfacial chemical environments regulated by anion-dependent adsorption in the Stern layer.*** *MD simulation snapshots showing molecular distributions near the Zn surface in* ***a*** *ZS+TEABF and* ***b*** *ZS+TEAHS electrolytes. Radial distribution profiles of* ***c*** *H_2O,* ***d*** *SO_4^{2-}, and* ***e*** *TEA$^+$ highlighting differential participation in the EEL.* ***f*** *Galvanostatic voltage response during initial Zn deposition and in-situ Raman spectra tracking TEA$^+$ and SO_4^{2-} evolution at the Zn interface in* ***g*** *ZS+TEABF and* ***h*** *ZS+TEAHS electrolyte.* ***i*** *Schematic illustration of the structural differences in molecular participation at the EEL, induced by competitive behavior between anion and cation.*'

R-MS, Page 13: '***Fig. 5 | Simulation and characterization of TEA$^+$ and SEI components. a*** *Snapshots from AIMD simulations depicting TEA existence on the Zn surface.* ***b*** *Changes in the length of C-N bond during MD simulation.* ***c*** *N 1s,* ***d*** *O 1s and* ***e*** *S 2p XPS depth profiles of Zn anodes after 10 cycles in ZS+TEABF and ZS+TEAHS electrolytes.* ***f*** *Three-dimensional view of elemental C-N distributions for cycled Zn in the TOF-SIMS sputtered volumes.* ***g*** *The HRTEM images of after three cycles of zinc plating/stripping on the Cu mesh under 2 mA cm^{-2}/1 mAh cm^{-2} in ZS+TEABF electrolyte. In-situ FTIR spectra of Zn||Cu cells in the* ***h*** *ZS+TEABF and* ***i*** *ZS+TEAHS electrolytes.*'

R-MS, Page 15: '*Fig. 6 | Mechanical robustness and Zn^{2+} transport properties of SEI.* *Representative Zn^{2+} migration pathways through SEIs derived from* ***a*** *ZS+TEAHS and* ***b*** *ZS+TEABF electrolytes, constructed based on experimental HRTEM and XPS data.* ***c*** *Corresponding Zn^{2+} migration energy barriers in the two SEIs. In-situ DRT spectra for Zn||Zn symmetric cells with* ***d*** *ZS+TEAHS and* ***e*** *ZS+TEABF electrolyte.* ***f*** *The calculated adsorption energy and desolvation energy of hydrated Zn^{2+} on SEIs and* ***g*** *activation energy fitted by the Arrhenius formula in different electrolytes.* ***h*** *AFM-derived modulus distributions of the outer SEI layers.*'

5. Line 114-116: SEM alone cannot confirm zinc sulfate by-products (ZSH). It provides only morphological information. Techniques such as XRD, EDS, or XPS should be used for chemical identification.

Response:

Thank you for your valuable suggestion. To identify the composition of the by-products, we performed supplementary experiments on the cycled zinc anodes. First, energy-

dispersive spectroscopy (EDS) surface mapping of a representative region (marked as the red area in **R-SI, Supplementary Fig. 1e**) revealed significantly enhanced signals from O and S, while the signal from Zn was comparatively reduced. Combined with its distinct polygonal flake-like morphology, these observations support the identification of this material as zinc sulfide hydroxide (ZSH). Furthermore, XRD analysis confirmed notable variations in ZSH diffraction peak intensities across different electrolytes. This trend aligns well with the ZSH density observed via SEM and EDS (**R-SI, Supplementary Table 1**), where all electrolytes containing TEA^{+} additives exhibited lower ZSH peak intensities compared to the ZS electrolyte. Collectively, these results demonstrate that the incorporation of TEA^{+}-based additives effectively suppresses the formation of ZSH by-products.

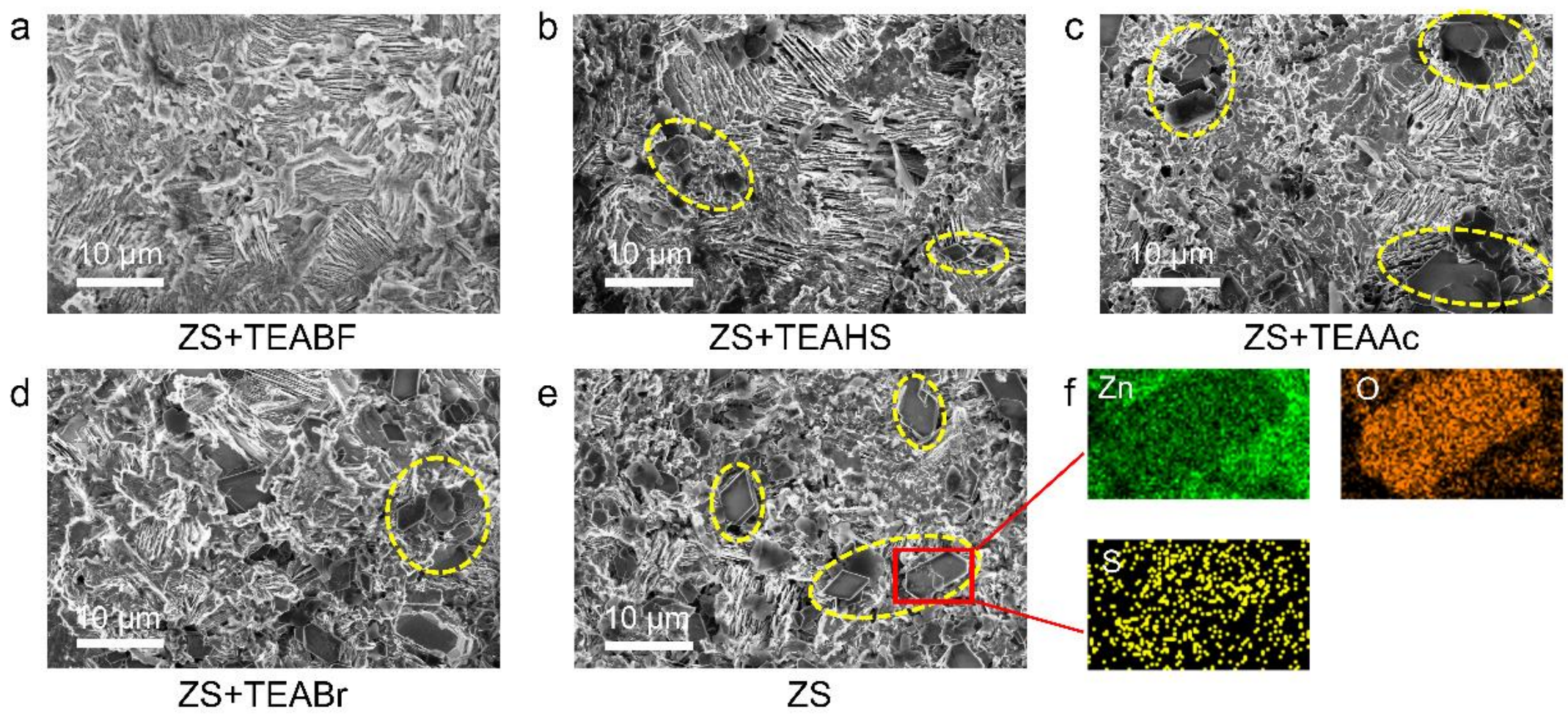

Supplementary Fig. 1. (a-e) SEM images of zinc anodes in different electrolytes after 30 cycles under 5 mA cm^{-2}/ 2.5 mAh cm^{-2}. (f) The elemental mappings of the polygonal thin sheets on the surface of the cycled zinc electrode.

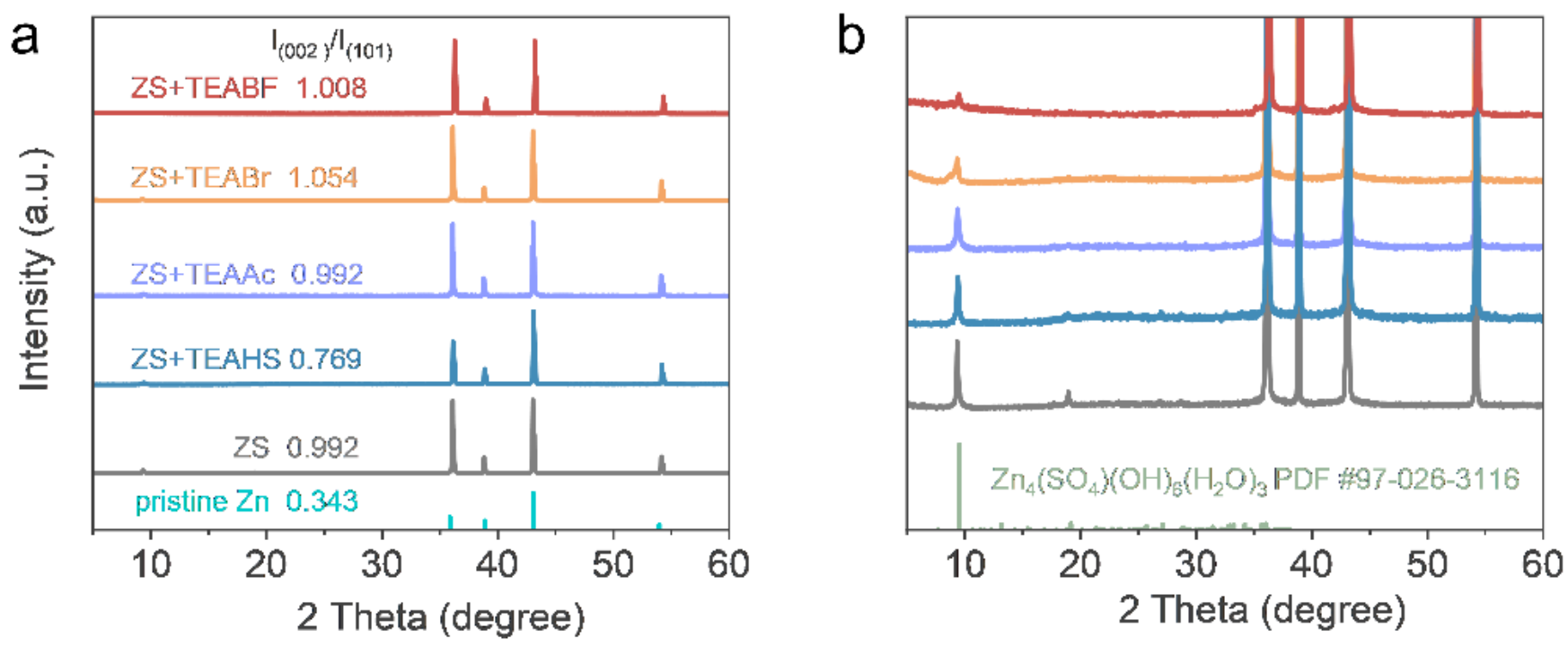

Supplementary Fig. 2. (a) XRD patterns and (b) locally magnified images of cycled zinc anodes in various electrolytes under 5 mA cm^{-2}/ 2.5 mAh cm^{-2}.

Supplementary Table 1. EDS analysis data of cycled zinc electrodes in different electrolytes.

wt%	Zn	O	C	S
ZS+TEABF	83.8	4.5	11.3	0.3
ZS+TEAHS	82.1	12.6	2.9	2.4
ZS+TEAAc	85.7	9.7	2.9	1.8
ZS+TEABr	85.0	9.7	3.3	1.9
ZS	78.2	15.7	3.3	2.6

The following provides the corresponding revised text description in R-SI.

R-MS, Page 5: '*The scanning electron microscopy (SEM) images indicate that, with the exception of the ZS+TEABF electrolyte, polygonal thin sheets can be detected on the surface of the cycled zinc electrode (indicated by the yellow markings in Supplementary Fig. 1). Based on combined local energy dispersive spectroscopy (EDS) mapping and X-ray diffraction (XRD) patterns of cycled Zn (Supplementary Fig. 2), this structure was identified as basic zinc sulfate (ZSH), a typical by-product formed during the zinc plating/stripping process. Notably, the introduction of additives into the electrolyte significantly reduced the formation of ZSH, suggesting that additives effectively mitigates side reactions (Supplementary Figs. 1, 2 and Supplementary Table 1).*'

6. Please clarify the meaning of the yellow dashed line in Supplementary Fig. 1.

Response:

Thank you for your valuable suggestions. By integrating the supplementary regional EDS surface scanning with XRD analysis results, we have identified the substance in this area as a typical by-product of basic zinc sulfate (ZSH). This finding has been clarified in the R-MS:

R-MS, Page 5: '*The scanning electron microscopy (SEM) images indicate that, with the exception of the ZS+TEABF electrolyte, polygonal thin sheets can be detected on the surface of the cycled zinc electrode (indicated by the yellow markings in Supplementary Fig. 1). Based on combined local energy dispersive spectroscopy (EDS) mapping and X-ray diffraction (XRD) patterns of cycled Zn (Supplementary Fig. 2), this structure was identified as basic zinc sulfate (ZSH), a typical by-product formed during the zinc plating/stripping process. Notably, the introduction of additives into the electrolyte significantly reduced the formation of ZSH, suggesting that additives effectively mitigates side reactions (Supplementary Figs. 1, 2 and Supplementary Table 1).*'

7. In Supplementary Fig. 2, specify whether the ionic conductivities were obtained experimentally or through MD simulations. It would be ideal if the authors could verify and compare the ionic conductivities and diffusion coefficients of the electrolytes between experimental measurements and MD simulations.

Response:

Thank you for your valuable suggestions. The ionic conductivity data presented in **Supplementary Fig. 2** were obtained through instrumental measurements. To further validate these results, we have supplemented the analysis with zinc ion diffusion coefficients calculated from the mean square displacement (MSD) in five different electrolyte systems. The calculated diffusion coefficients show good agreement with the experimentally measured ionic conductivities. Notably, the ZS+TEABF electrolyte exhibits higher ionic conductivity, which can be attributed to the relatively weak hydration tendency of BF_4^- anions, their reduced interaction with zinc ions, and lower charge density. In contrast, the ionic conductivity of the ZS+TEAAc electrolyte is lower than that of the baseline ZS electrolyte. This decrease may be due to the presence of polar functional groups in Ac^-, which can strongly coordinate with zinc ions, combined with significant steric hindrance that likely increases solution viscosity. The supplementary MSD-derived diffusion coefficients are provided below.

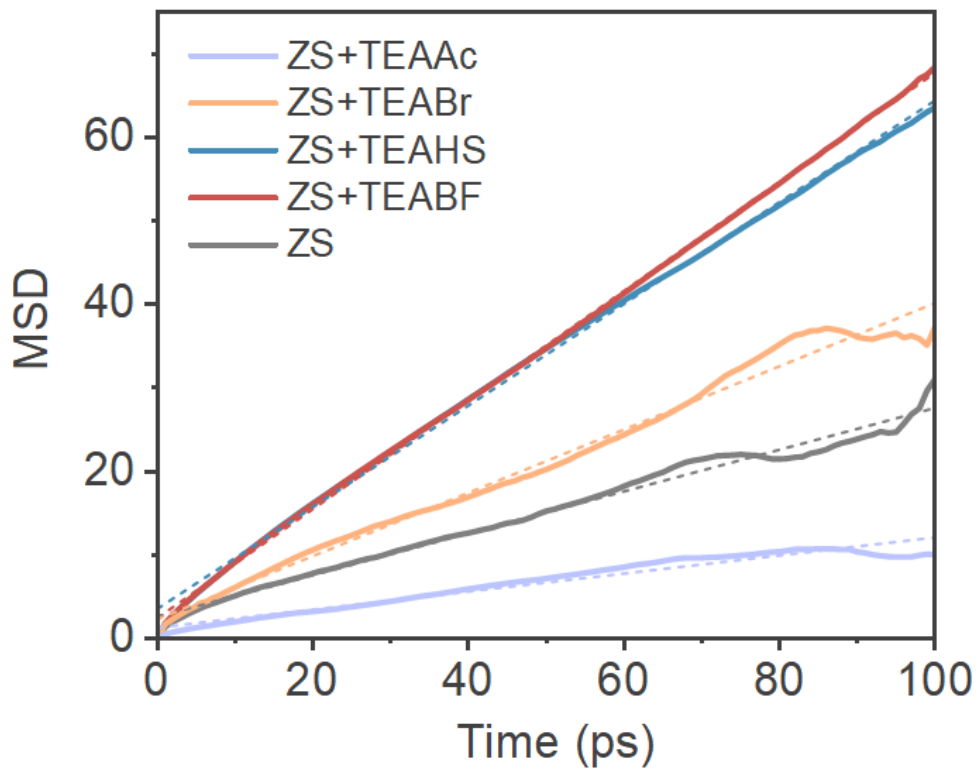

Supplementary Fig. 3. (b) The mean square displacement (MSD) results of Zn^{2+} in different electrolytes.

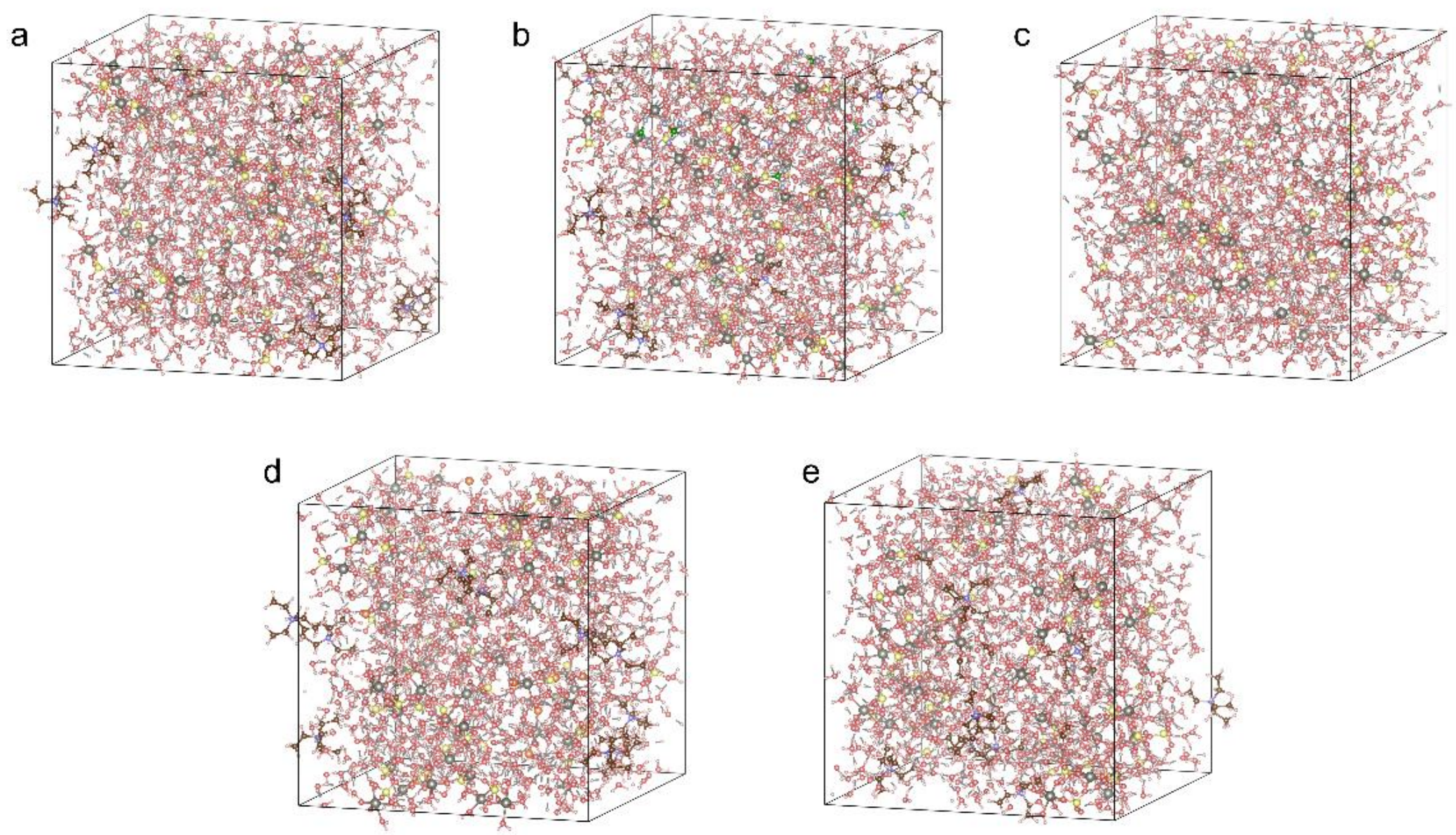

Supplementary Fig. 4. The MD results of (a) ZS+TEAHS, (b)ZS+TEABF, (c) ZS, (d)

ZS+TEABr and (e) ZS+TEAAc electrolytes.

Supplementary Table 2. MSD results of Zn^{2+} in different electrolytes.

**Electrolytes**	**D_{Zn2+} (Å^2 ps^{-1})**	**R^2**
ZS+TEABF	0.652	0.99946
ZS+TEAHS	0.608	0.99817
ZS+TEABr	0.378	0.98563
ZS+TEAAc	0.108	0.94752
ZS	0.250	0.98488

The following presents the corresponding textual description in R-MS.

R-MS, Page 5: '*In addition to the ZS+TEAAc electrolyte, the incorporation of various additives has generally enhanced the ionic conductivity of the electrolyte to some extent, albeit with minor differences among them. This trend is consistent with the results of Zn^{2+} diffusion coefficients derived from molecular dynamics (MD) simulations (Supplementary Figs. 3, 4 and Supplementary Table 2).*'

8. Clarify the difference between the ZS+TEAHS data shown in Fig. 1a and Supplementary Fig. 3.

Response:

Thank you for your suggestion. The data presented in Fig. 1a (**R-MS, Fig. 2a**) illustrate the cycle life of the Zn||Zn symmetric cell employing an untreated ZS+TEAHS electrolyte with a pH of 2.27. The observed cycling stability of the zinc anode in this electrolyte is relatively limited, which may be attributed to the highly acidic environment. To mitigate the influence of pH variations, $Zn(OH)_2$ was introduced into the electrolyte to adjust its pH to 2.8—matching that of the ZS+TEABF electrolyte. Subsequently, the cycling performance of the zinc anode was re-evaluated, as shown in Supplementary Fig. 3 in original SI. The corresponding statements in the manuscript are as follows.

R-MS, Page 5: '*In contrast, significant variations in pH were observed, which could be attributed to differences in the degree of anion hydrolysis (Supplementary Fig. 3a). Although the pH of the ZS+TEAHS electrolyte was adjusted to match that of the ZS+TEABF electrolyte by adding $Zn(OH)_2$, the performance of the zinc anode demonstrated considerable discrepancies (276 h vs. 850 h, Fig. 2a and Supplementary Fig. 5), highlighting that anion identity plays a crucial role even at low additive concentrations (10 mM).*'

9. A brief introduction should be added before line 166 to guide the reader into the next section.

Response:

We sincerely appreciate your valuable suggestions. In response, we have incorporated additional narrative elements into R-MS to enhance contextual coherence. The revised

content is provided below.
R-MS, Page 7: '*The ions and molecules present in the electrolyte serve as key components of the EDL. Owing to their distinct affinities for the electrode surface, these species exhibit varying concentrations within the EDL, thereby significantly influencing the electrochemical behavior. To elucidate the surface adsorption behavior of different additives, we first performed theoretical analyses using density functional theory (DFT) calculations.*'

10. In Fig. 2b, confirm whether the label should be "ZS+TEAHS" or "ZA+TEAHS."
Response:
We sincerely appreciate your valuable suggestion and apologize for the errors resulting from our oversight. The necessary revisions have been duly implemented.

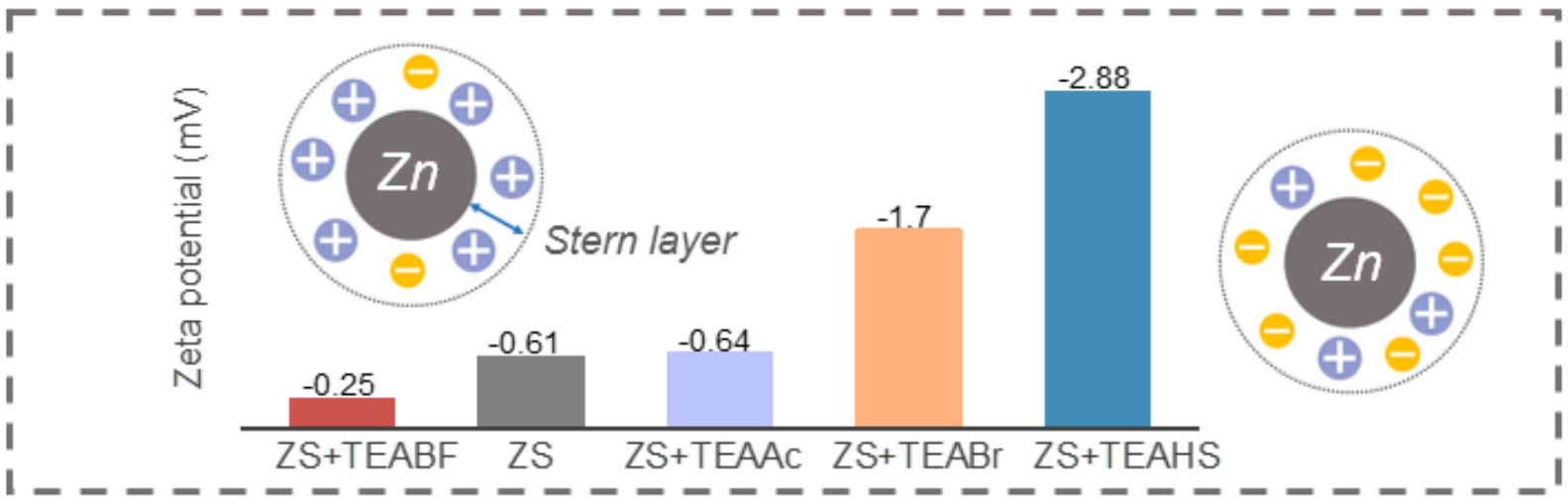

Fig. 3 | b Zeta potentials of Zn surfaces in different electrolytes, reflecting variations in ion distributions within the Stern layer.

11. In Fig. 2c, please justify the use of Na_2SO_4-based electrolytes.
Response:
Thank you for your valuable suggestions. The primary objective of measuring differential capacitance is to examine the distribution of species within the electrical double layer (EDL) under varying electrolytes. In zinc salt-containing electrolytes, the Faraday current arising from the zinc plating/stripping process can induce irreversible changes in the electrode surface structure and condition, thereby compromising the accurate characterization of the EDL. To eliminate this interference, $ZnSO_4$ was substituted with Na_2SO_4 (*Nat. Commun., 2025, 16, 3142*). The corresponding explanations have been supplemented and clarified in the R-MS.
R-MS, Page 7: '*Differential capacitance measurements in Na_2SO_4-based electrolytes further corroborate this behavior. Na_2SO_4 was employed as an alternative to $ZnSO_4$ to eliminate potential interference from Faraday currents.*'

12. In Fig. 2d, please indicate the scan rate and potential range used for cyclic voltammetry.
Response:
We appreciate your valuable suggestions. Providing comprehensive details is essential for improving the rigor of the manuscript. In Fig. 2d (**R-MS, Fig. 3d**), the sweep rate was set to 1 mV s^{-1} with a voltage range spanning from -0.2 V to 0.2 V. This information

has been added to the R-MS.

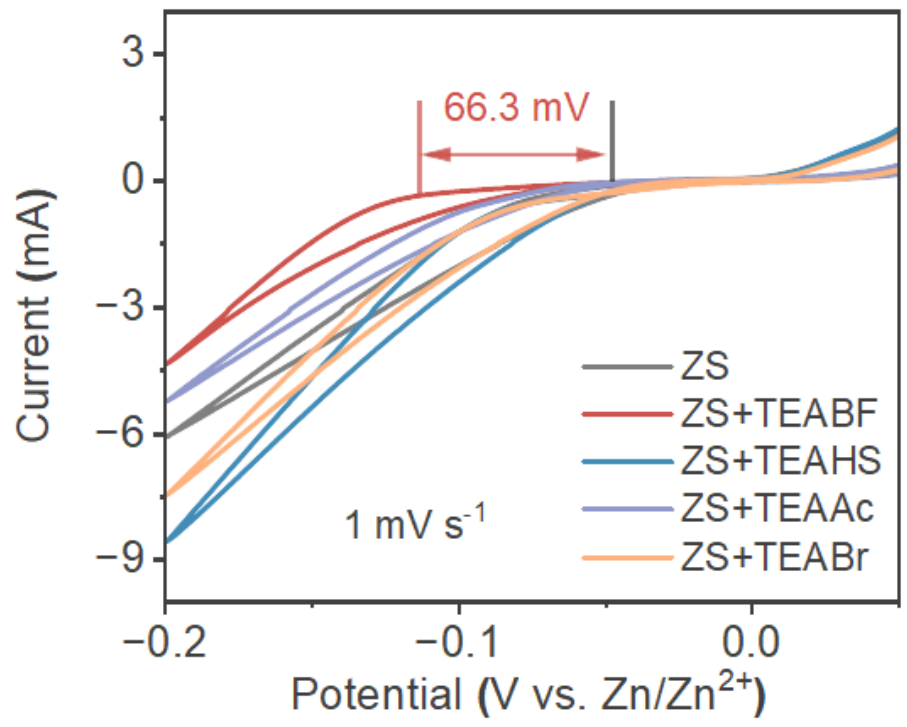

Fig. 2 | d Cyclic voltammetry of Zn||Zn symmetric cells conducted from -0.2 to 0.2 V.

13. Fig. 3f is not referenced in the text. Please ensure it is properly discussed and includes the time scale on the y-axis.

Response:

Thank you for highlighting this matter. As illustrated in Fig. 3f (**R-MS, Fig. 4f**), the plot depicts the voltage-time profile obtained from constant current cycling applied to the electrolytic cell during the in-situ Raman spectra measurements, which were performed to investigate the electrode–electrolyte interface. Clarifying explanations have been added in the R-MS to enhance the understanding of this experimental procedure.

R-MS, Page 9: '*To further validate the competitive localization of anions and TEA^+ in the Stern layer, in-situ Raman spectra was employed to probe species distribution on the zinc surface during the initial constant-current charge-discharge process (Fig. 4f).*'

14. Supplementary Fig. 7, please provide the equivalent circuit model used to fit the EIS spectra and specify the frequency range. Please explain why the temperature range of 30-70 °C was chosen instead of 0-50 °C.

Response:

We sincerely appreciate your valuable suggestions. We have supplemented the equivalent circuit diagrams used in this study. Furthermore, the frequency range for the EIS measurements in this work was set between 0.01-100,000 Hz.

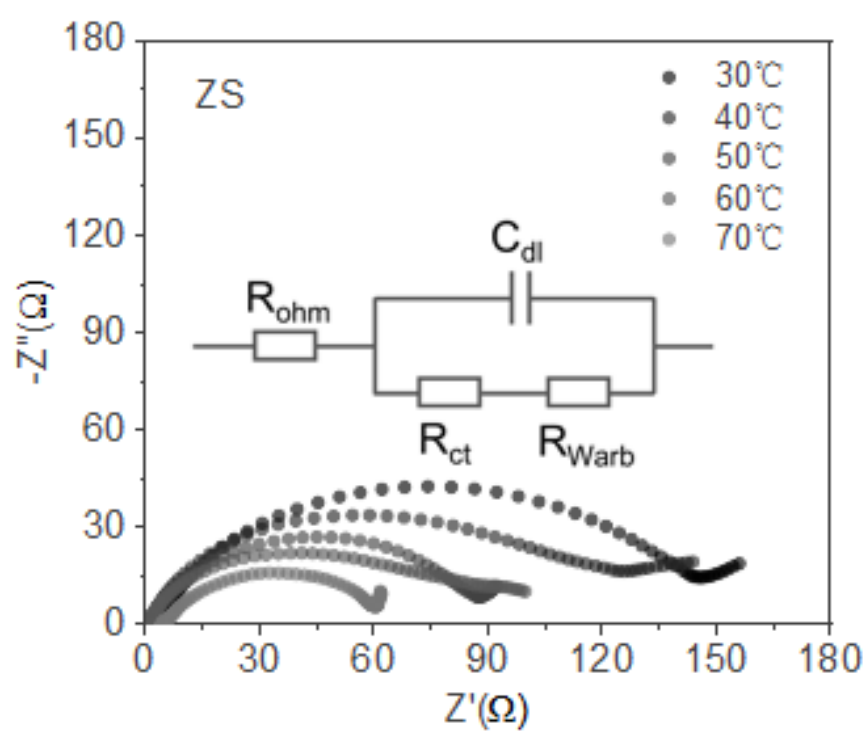

Supplementary Fig. 21. (c) EIS test of symmetric batteries in different electrolytes at

different temperatures

R-MS, Page 21: '*EIS measurements were performed using CHI660E electrochemical workstation over a frequency range of 0.01 to 100,000 Hz. The EIS tests at different temperatures were performed by placing the coil-symmetric cells in a temperature-controlled oven. The obtained EIS data were analyzed using 'DRT tools'.*'

In the preliminary experiments, the temperature range was limited to 30–70 °C due to the constraints of the constant temperature furnace, which could not operate below ambient temperature (25 °C). In subsequent experiments, a wide-temperature-range environmental chamber was introduced, enabling supplementary EIS measurements within the 0–50 °C range. The re-tested data show that the activation energy (E_a) of Zn^{2+} is highest in the ZS+TEABF electrolyte and lowest in the ZS electrolyte, consistent with prior experimental findings. It should be noted that the E_a, derived from temperature-dependent EIS measurements, can be considered approximately constant across the investigated temperature intervals. However, due to the pronounced slowing kinetic of battery at lower temperatures, the E_a values obtained in the 0–50 °C range are systematically higher than those observed in the 30–70 °C range.

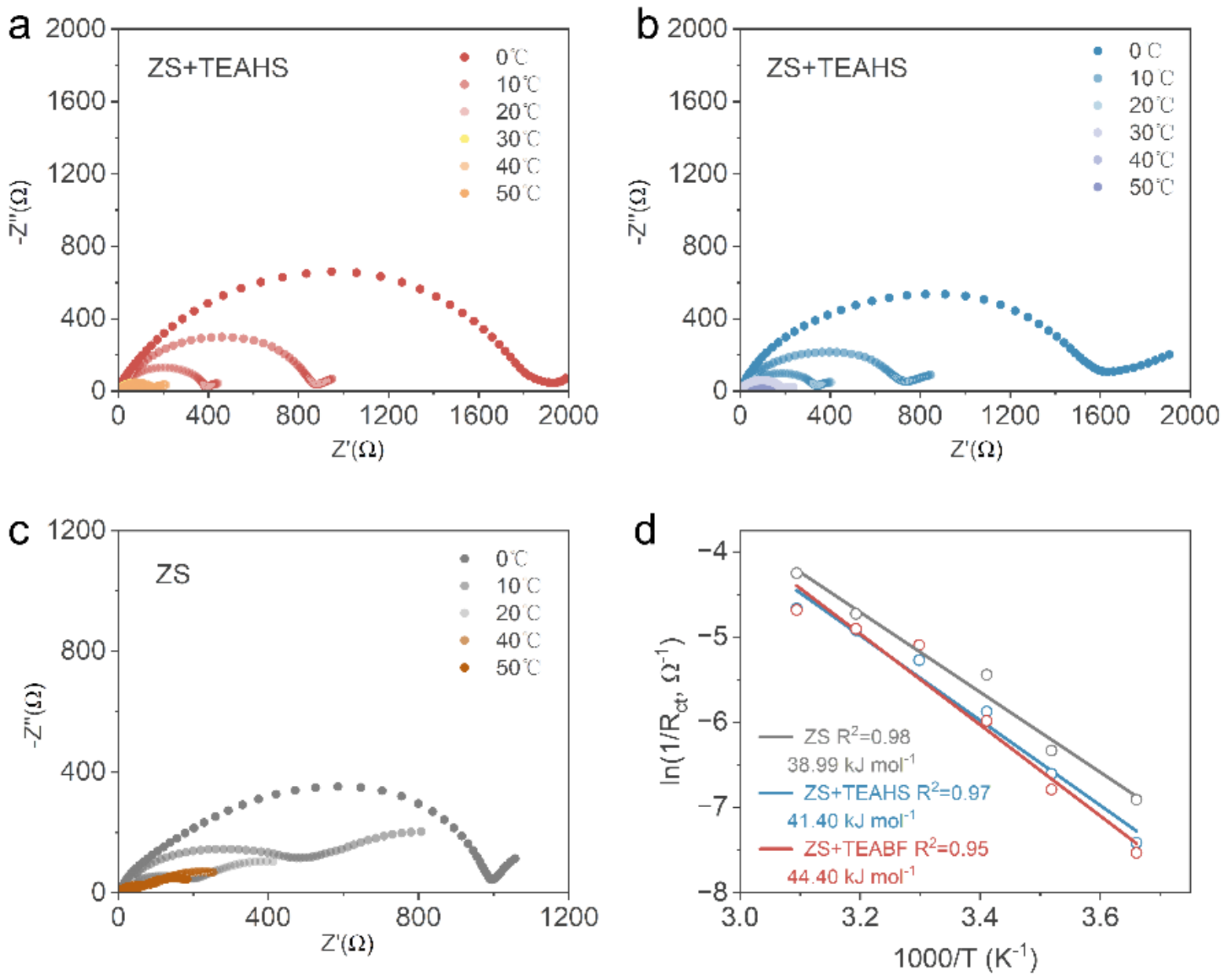

(a-c) EIS test of symmetric batteries in different electrolytes at 0-50 °C and (d) the activation energy fitted by the Arrhenius equation.

15. Fig. 5g, R^2 value should be included.

Response:

Thank you very much for your reminder. We have incorporated R^2 in **Supplementary Fig. 21d**. Among these, the R^2 values all exceeded 0.97, indicating a high degree of goodness of fit and demonstrating that the model fitting results are highly satisfactory.

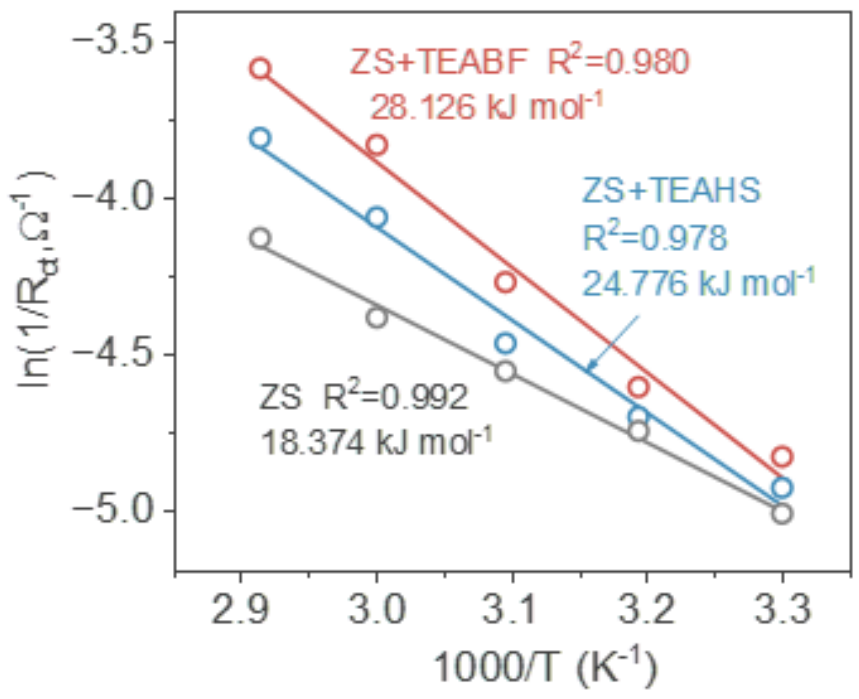

Supplementary Fig. 21. (d) The activation energy fitted by the Arrhenius equation.

16. Define all chemical abbreviations (e.g., EDOT, PEDOT, PVDF, NMP, CNT, etc.) when first introduced.

Response:

We sincerely appreciate your reminder. All abbreviations of chemical substances have now been supplemented with their full names in the R-MS. The updated content is provided below.

R-MS, Page 16: '*Moreover, the full cells using poly (3,4-ethoxylene dioxy thiophene) (PEDOT)-V_2O_5 (PVO) cathode are assembled in the ZS+TEAHS and ZS+TEABF electrolytes to evaluate practical value.*'

R-MS, Page 19: '*To prepare the paste for coating, 80wt% PVO, along with 10wt% polyvinylidene fluoride (PVDF) and 10wt% carbon nanotube (CNT) conductor were dissolved uniformly in N-Methyl-2-pyrrolidinone (NMP) and subjected to magnetic stirring for a duration of 6 h.*'

17. Include the source and purity of all chemicals used in the experiments.

Response:

Thank you for your valuable suggestion. We fully concur with your recommendation, as providing detailed information on the source and purity of chemicals is essential for ensuring experimental reproducibility. We have now updated the documentation for all chemical substances accordingly.

R-MS, Page 19: '***Preparation of ZS electrolyte and additive electrolyte***

143.78 g $ZnSO_4{\cdot}7H_2O$ (99.5%, Innochem) was dissolved in deionized water to form 250 mL homogeneous solution for the preparation of 2 M $ZnSO_4$ electrolyte, which was denoted as ZS electrolyte. Then, different moles of tetraethyl ammonium tetrafluoroborate ($TEABF_4$, 99%, Innochem) powder were dissolved in 2 M $ZnSO_4$ electrolyte respectively to prepare hybrid electrolytes containing different concentrations (0.005, 0.01, 0.03 and 0.1 M) $TEABF_4$ (denoted as ZS+TEABF electrolyte). The electrolytes with different additives were prepared by introducing $TEAHSO_4$ (99%, Aladdin), TEABr (99%, Innochem) and TEAAc (99%, Adamas-beta) in the same method. Commercially available $TEAHSO_4$ (99%, Apinno) was selected because battery-grade $(TEA)_2SO_4$ is neither available nor reliably synthesized;

repeated laboratory attempts produced hygroscopic solids with residual acid. In aqueous solution $TEAHSO_4$ is easily deprotonated, thereby delivering the same TEA^+/SO_4^{2-} pair required for this study. The alkalization of ZS+TEAHS electrolyte was achieved through the addition of $Zn(OH)_2$ (99%, Tianjin Damao).'

18. The material characterization section should be expanded with sufficient details for the work to be reproduced.

Response:

Thank you very much for your valuable suggestion. We have supplemented the XRD, SEM and HRTEM characterizations of the PVO cathode material. In this study, PVO was selected as the research focus based on the following considerations: EDOT is intercalated between the V_2O_5 layers and undergoes in situ polymerization, which effectively reduces the crystallinity of V_2O_5 and enhances its structural adaptability to the significant volume changes induced by repeated Zn^{2+} insertion and extraction during cycling (*Chem. Eng. J. 2024, 484, 149501*). XRD analysis reveals a marked reduction in the crystallinity of PVO, with the characteristic diffraction peaks of V_2O_5 significantly diminished. Furthermore, sulfur signals are observed in the EDS elemental mapping corresponding to the SEM images, and HRTEM results indicate an expansion of the (220) lattice spacing of V_2O_5 to 2.061 Å. These findings collectively confirm the successful intercalation and intralayer polymerization of EDOT within the V_2O_5 framework.

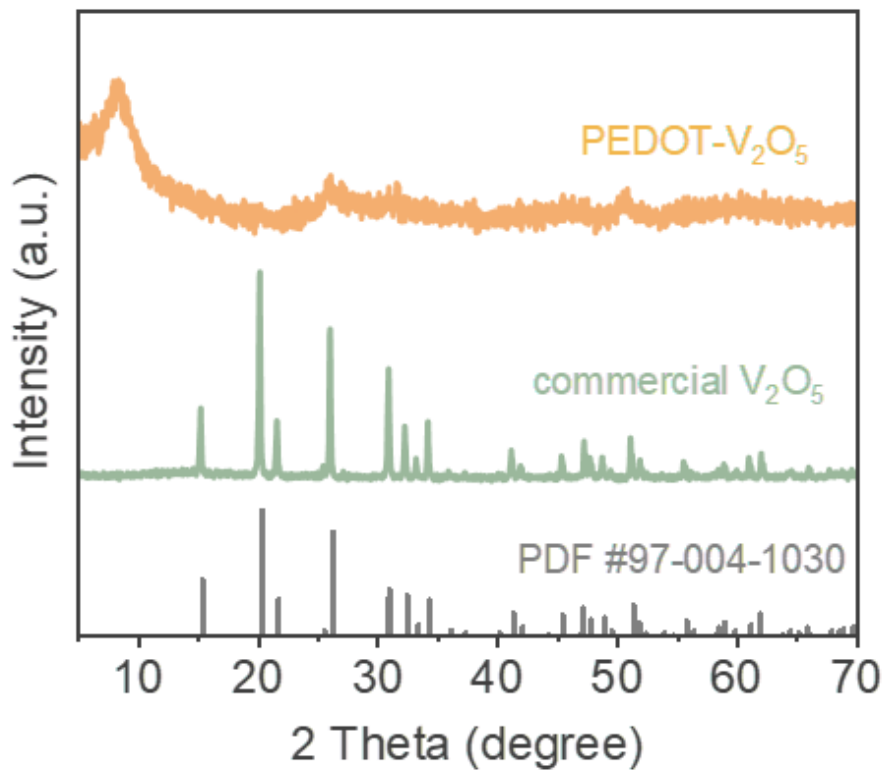

Supplementary Fig. 30. XRD pattern of the PEDOT-V_2O_5.

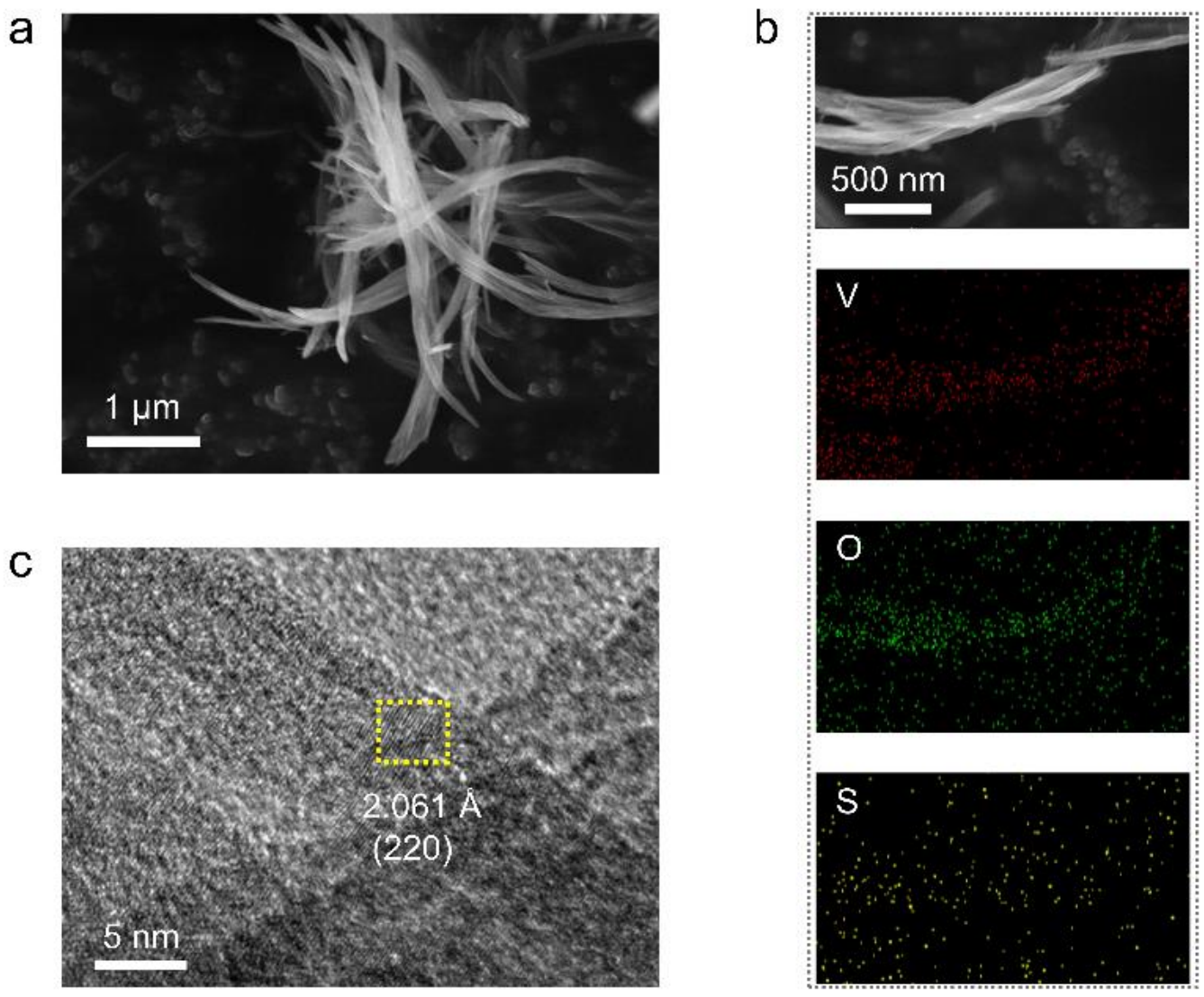

Supplementary Fig. 31. (a) SEM image and (b) EDS elemental mapping of the PVO. (c-d) The lattice spacing of the V_2O_5 (220) plane increases from 2.044 to 2.061 Å due to the intercalation and polymerization of EDOT.

In our R - MS, the following discussion content has been incorporated on Pages 16 and 17:

R-MS, Page 16: '*Moreover, the full cells using poly (3,4-ethoxylene dioxy thiophene) (PEDOT)-V_2O_5 (PVO) cathode are assembled in the ZS+TEAHS and ZS+TEABF electrolytes to evaluate practical value. The incorporation and internal polymerization of EDOT can reduce the crystallinity of V_2O_5, thus mitigating severe structural phase transformations induced by Zn^{2+} insertion/extraction during cycling (Supplementary Figs. 30, 31). Furthermore, this modification introduces additional active sites, facilitating faster zinc ion transport kinetics (Supplementary Fig. 32)*[40].'

19. The procedure for HRTEM and FFT indexing should be included in the material characterization section.

Response:

Thank you for your valuable suggestion. We have incorporated the relevant information into R-MS accordingly.

R-MS, Page 20: '*High-resolution transmission electron microscopy (HRTEM) was performed using a JEM-2200FS microscope and analyzed by DigitalMicrograph.*'

20. Experimental details for AFM, DRT, and EIS measurements should also be included in the Methods section.

Response:

Thank you for your valuable suggestion. We have incorporated detailed procedural steps and comprehensive data processing information into the Methods section of R-MS. The specific supplementary content is provided below.

R-MS, Page 20: '*Atomic force microscopy (AFM, Dimension Icon, Bruker, Germany) was employed to evaluate the surface height variations of the zinc anode and the Young's modulus of the SEI.*'

R-MS, Page 21: '*EIS measurements were performed using CHI660E electrochemical workstation over a frequency range of 0.01 to 100,000 Hz. The EIS tests at different temperatures were performed by placing the coil-symmetric cells in a temperature-controlled oven. The obtained EIS data were analyzed using 'DRT tools.*'

Reviewer #3:

Thank you for sharing interesting work. I have several recommendations to validate the methods you used to support the claim on mechanisms.

Response:

We express our sincere gratitude for your review of our research and for the valuable and constructive suggestions you have provided. In particular, we are highly appreciative of the suggestion regarding the necessity for further verification of the components of the SEI. In accordance with your suggestion, we have additionally conducted supplementary verification utilizing Ab Initio Molecular Dynamics (AIMD) simulations and ^{13}C solid-state nuclear magnetic resonance (NMR) spectra. These supplements aim to clarify the organic components within the SEI. The experimental results indicate that TEA^{+} demonstrates excellent stability in the battery operating environment and does not undergo decomposition reactions. Furthermore, regarding the relatively simplistic model system employed during our exploration of the influence of the SEI on the transport behavior of Zn^{2+}, the primary objective was to emphasize the crucial influence of the organic in the SEI on zinc deposition behavior. Naturally, we also acknowledge the limitations of the current model and will conduct more in-depth and precise mechanism studies in our subsequent work. Once again, we extend our heartfelt thanks for your assistance in significantly enhancing the scientific rigor and argumentation quality of this research. We sincerely hope that the revised manuscript (R-MS) can comprehensively address all your concerns.

Below, each response related to your comment is presented in blue font, and the revised sections in R-MS are highlighted in yellow.

1. The authors need to identify the surface observed in the experiment, at the moment the justification of adsorbate interaction with the Zn 002 surface is not provided. Different additives used in the battery set-up can lead to preferential formation of 002, 101, or 100 surfaces. Without clear experimental justification, the trends observed from experiment cannot be independently verified.

Response:

Thank you for this constructive suggestion. To experimentally justify the surface model used in the simulations, we performed XRD measurements on the zinc electrodes after 100 cycles in all electrolytes (**R-SI, Supplementary Fig. 2**). The data show that the (002) diffraction peak becomes significantly more pronounced after cycling, and the intensity ratio $I_{(002)}/I_{(101)}$ increases markedly relative to the pristine Zn foil. This indicates that during repeated plating/stripping process, zinc tends to recrystallize preferentially along the (002) plane, consistent with its lowest surface energy among the major Zn facets (*PNAS, 2024, 121, e2309981121*).

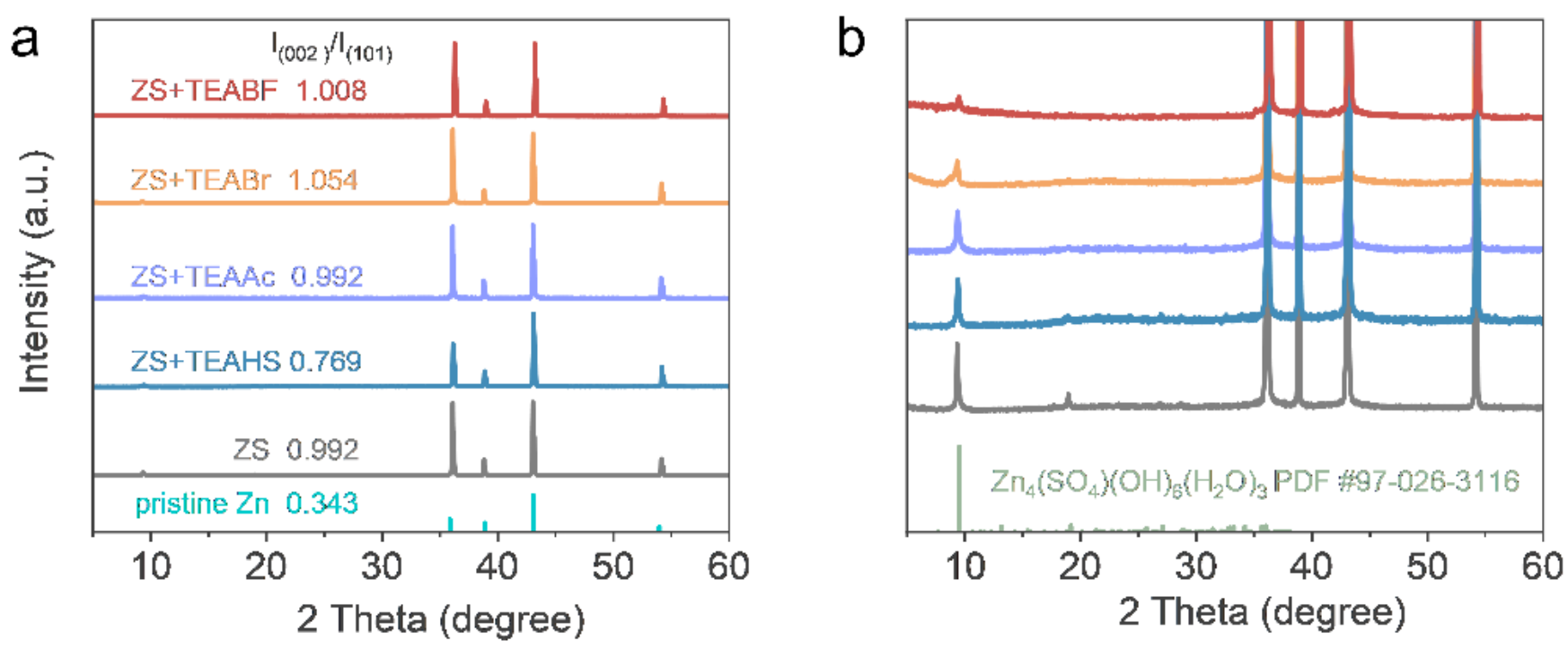

Supplementary Fig. 2. (a) XRD patterns and (b) locally magnified images of cycled zinc anodes in various electrolytes under 5 mA cm^{-2}/ 2.5 mAh cm^{-2}.

Based on this experimental evidence, and supported by literature consensus that the (002) basal plane is the thermodynamically preferred growth surface under aqueous Zn cycling conditions, we selected the Zn (002) surface as the representative model for evaluating additive adsorption behavior. We have added a clarifying statement in the R-MS to explicitly justify this choice.

R-MS, Page 22: '*The Zn (002) crystal plane was chosen as the substrate for molecular adsorption because both theoretical and experimental studies have confirmed that preferential epitaxial growth of the (002) crystal plane occurs during the zinc deposition process.*'

2. The authors must clearly mention whether they calculate neutral adsorbate or charged adsorbate on the Zn surface. It appears to me that the calculations were done in neutral adsorbate, so the discussion must be revised accordingly, by not explicitly mention anions, but rather neutral adsorbate. But, if its not the case, the authors need to better explain how they calculate the anion adsorption on the surface.

Response:

Thank you for pointing out this important clarification. The reviewer is correct that the adsorption calculations must explicitly state the charge state of the adsorbates. In our study, all surface-adsorption calculations were performed using a charge-neutral cluster or molecule, following the widely adopted neutral-adsorbate approximation for metal–electrolyte interfaces in vacuum DFT calculations. This approach avoids introducing an artificial net charge into the periodic slab model and is commonly used when modelling ion-surface interactions in the absence of an explicit solvent or countercharge. We have now clarified this in the R-MS and adjusted the discussion to avoid any implication that charged species were directly adsorbed on the Zn surface in the DFT model.

R-MS, Page 7: '*The results reveal that the neutral TEA exhibits the strongest affinity for the Zn (002) surface among electrolyte species, while the BF_4 shows the weakest adsorption.*'

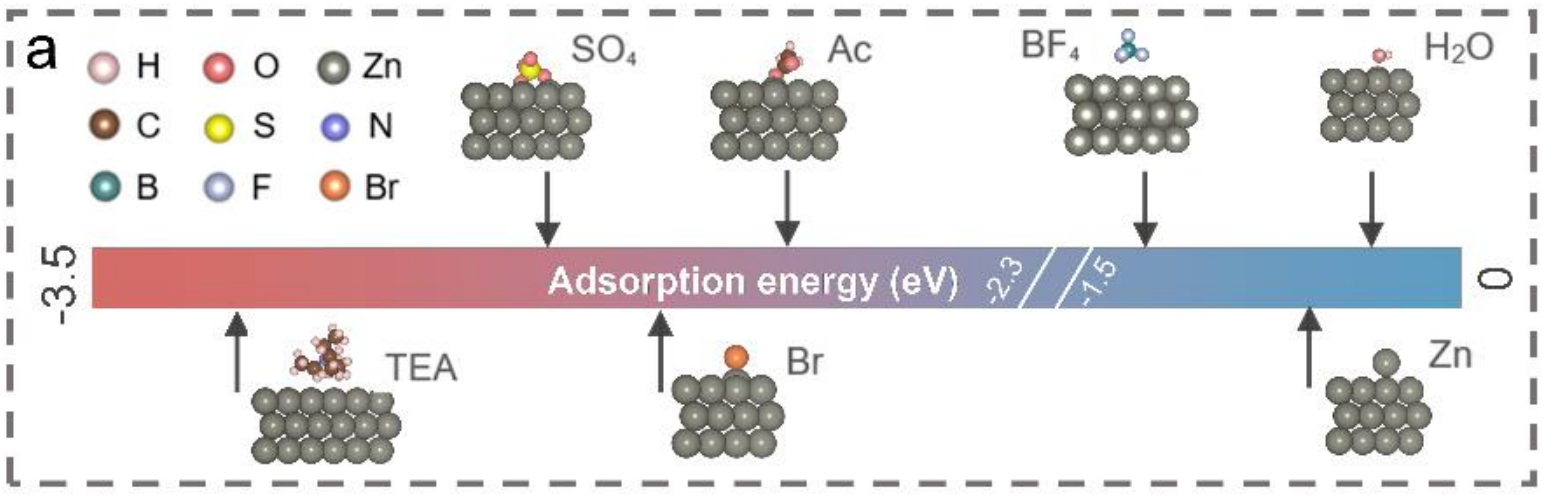

Fig. 3 | a Calculated adsorption energies of various electrolyte species on Zn (002), with insets illustrating their preferred binding configurations.

Supplementary Table 3. Adsorption energy of different neutral species on Zn (002).

	TEA	Zn	H_2O	CH_3COO	Br	SO_4	BF_4
E_{ad} (eV)	-3.32	-0.64	-0.39	-2.62	-2.78	-2.92	-1.31

3. The authors conducted very nice FFMD simulations both in the electrolyte and at the interface. However, they use different methods for both simulations. Can the authors better explain the reason for using different methods, especially the use of Forcite Module? Can the authors give justification how their models consistent with experiment results? Probably by comparing the density, component analysis (solvation structure), and so on?

Response:

Thank you for your positive feedback and constructive suggestions. When investigating the single molecule dissociation process of TEA additive on the zinc surface, as this process involves no more than 1000 atoms and can precisely depict the behavior of molecular bond breakage, there is no necessity to construct a large-scale electrode surface model. Consequently, the Forcite Module in Materials Studio is employed for calculations in this part. This tool can effectively and accurately simulate such surface reaction processes. Regarding the electrolyte system, it is essentially an unordered structure, and a large-scale model (comprising more than 10,000 atoms) needs to be constructed to comprehensively reflect its unordered characteristics. If the Forcite Module is still utilized at this juncture, the computational load will increase significantly, and the calculation time will be prolonged. Therefore, Gromacs, which has superior computational performance, is adopted for processing.

Furthermore, in response to the fifth comment raised by the reviewers, to further demonstrate the rationality of our SEI model, specifically, the existence form of the organic components in the SEI, we substituted the original FFMD simulation part with a more precise AIMD method to acquire highly accurate simulation results.

Regarding the agreement between theoretical calculations and experimental results.

(I) The density of electrolyte:

The ZS electrolyte (2M $ZnSO_4$ in water solvent) is taken as an example. An NPT ensemble equilibrium simulation was carried out at the temperature (298 K) and

pressure (1 atm) consistent with the experimental conditions. After the system density converged, the simulated density was calculated to be 1.44 g cm^{-3}. The error between this simulated density and the experimental density data reported in the literature (*J. Chem. Eng. Data 2021, 66, 38-44*) is approximately 6 - 8%. This error may be attributed to the approximate description of the molecular polarization effect by the fixed-charge force field employed and it falls within an acceptable range.

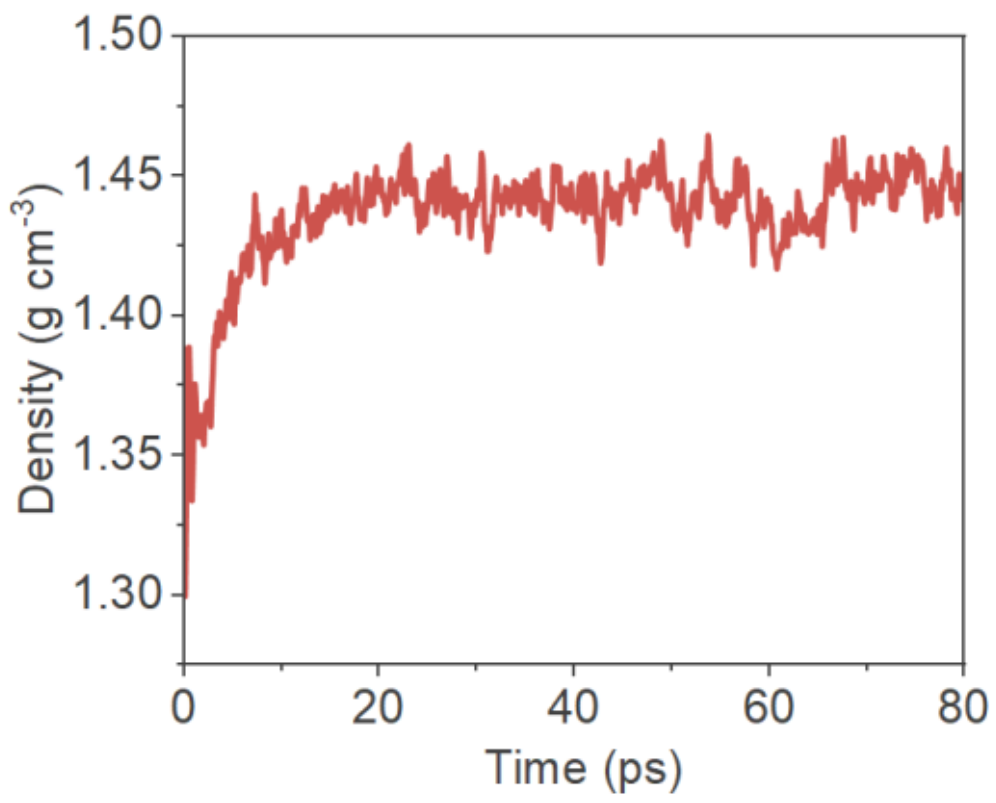

Statistical analysis of the electrolyte density during the simulation period

(II) Zn^{2+} solvation structure:

We conducted a further comparison between the Zn^{2+} solvation structure derived from the MD simulation and the experimental data obtained from Raman spectra. In the three target electrolytes, the characteristic spectral peaks of Zn-OH_2 were identical (*Adv. Mater. 2021, 33, 2102415*), showing no significant deviation. This phenomenon suggests that the water coordination configuration of Zn^{2+} has not experienced substantial changes. Moreover, according to the classical Eigen-Tamm (ET) mechanism, sulfate anions can form two coordination forms in the electrolyte: contact ion pairs (CIP) and solvent-separated ion pairs (SSIP) (*ACS Nano, 2023, 17, 552-560*). Through peak separation fitting and quantitative analysis of the SO_4^{2-} characteristic peaks, it was found that Zn^{2+} predominantly existed in the CIP form in all three electrolytes, and the relative proportion of SSIP/CIP was nearly the same, which directly indicates that no other components intervened in the Zn^{2+} solvation shell to alter its coordination state with SO_4^{2-}. The above spectral observation conclusions are in excellent agreement with the Zn^{2+} solvation structure revealed by the MD simulation in Fig. 1c (**Fig. 2c in R-MS**), fully validating the reliability and accuracy of the theoretical model in characterizing the microscopic coordination environment of the electrolyte.

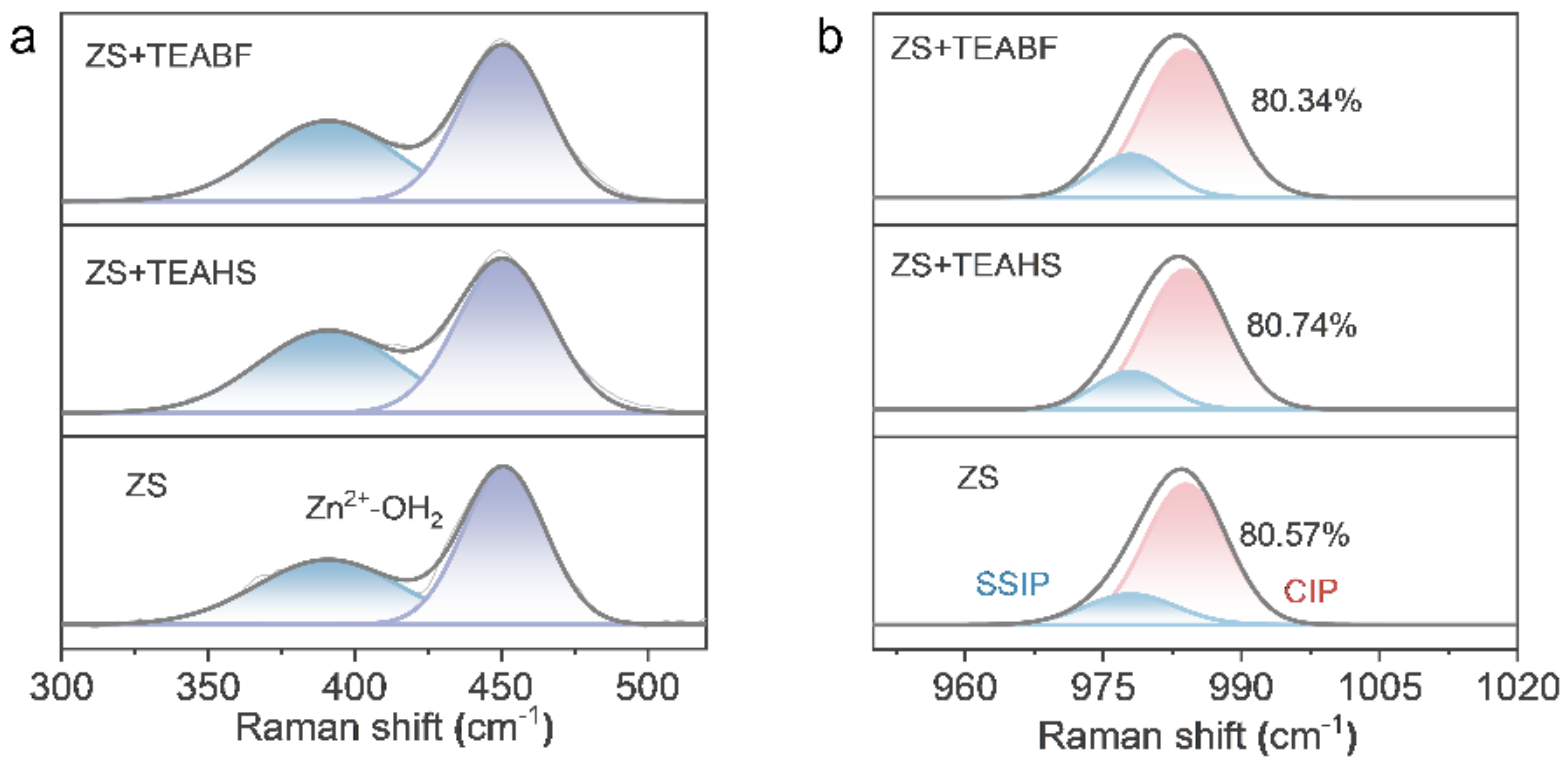

The detailed analysis is carried out on the (a) Zn-OH_2, (b) ν-SO_4^{2-} band in Raman spectra of ZS, ZS+TEAHS and ZS+TEABF electrolytes.

4. Regarding the validation of electrochemistry kinetics, did the authors able to verify whether the electrolyte components are stable on the surface? Are you able to check, probably through the CV or any surface characterisation? Although the systems are simplified, but the use of such model if not verified can lead to poor justification of mechanisms.

Response:

Thank you for highlighting this critical point.

(I) Verification of SEI components

Through surface detection techniques such as XPS and HRTEM, we have proven that certain components in the electrolyte can participate in the formation of the SEI as reduction products or reactants during the electrochemical process. For instance, in the ZS and ZS+TEAHS electrolytes, the SEI primarily comprises substances originating from hydroxyl/water and decomposition products associated with sulfates. In the ZS+TEABF electrolyte, the formed SEI mainly consists of inorganic components $ZnSO_3$ and $ZnCO_3$, while the organic component rich in the outer layer is TEA. Concerning the form of the organic components, we have carried out a series of experiments to elucidate this.

First, the CV of deionized water (DIW) either with or without 0.01 M $TEABF_4$ were measured within an appropriate voltage window (0–1.2 V, using two clean Pt foils as the working and counter electrodes and a saturated calomel electrode as the reference electrode). At a scanning rate of 2 mV s^{-1}, following 5 cycles of cyclic testing, no other distinct redox reaction signals were detected, apart from a minor difference in the response current. This indicates that TEA^+ did not undergo electrochemical decomposition within this potential range.

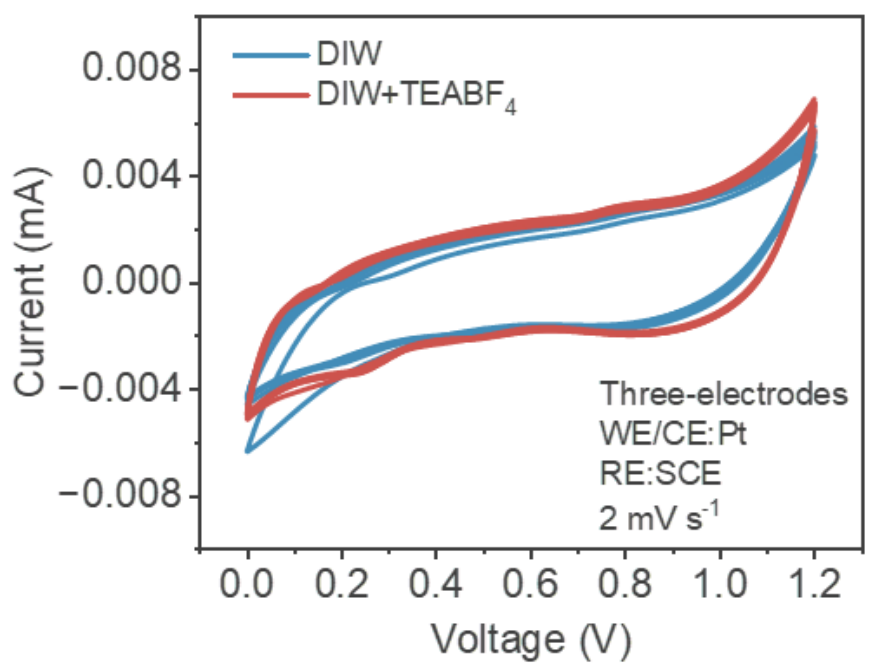

Supplementary Fig. 12. The CV curves of the DIW with or without 0.01 M $TEABF_4$ were measured within the potential range of 0–1.2 V.

Furthermore, we carried out a ^{13}C magic-angle spinning (MAS) solid-state nuclear magnetic resonance (ssNMR) analysis on the SEI samples formed on the electrode surface after 30 cycles. The results indicated that the spectra were largely consistent with the $TEABF_4$ standard sample, presenting two main characteristic peaks: the peak at 9.8 ppm is attributed to α-C ($–CH_3$), whereas the peak at 50.9 ppm corresponds to β-C ($N^+–CH_2$). This outcome further verified that TEA^+ could exist stably in the formed SEI without structural degradation.

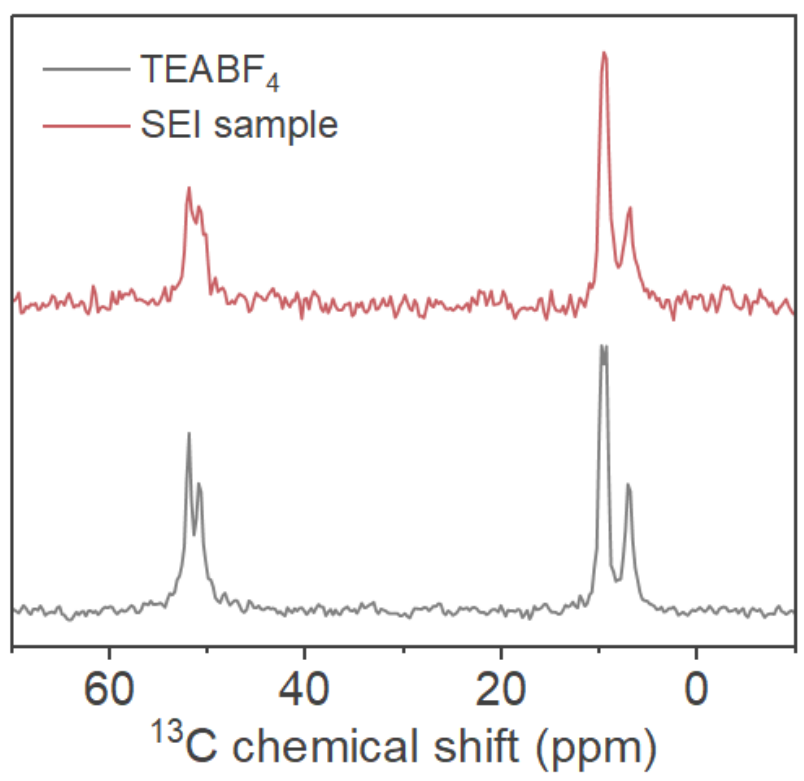

Supplementary Fig. 14. The ^{13}C NMR spectra of SEI sample derived from cycled zinc electrode and $TEABF_4$ powder. The zinc electrode was retrieved from Zn||Zn symmetric cell after 10 cycles under the condition of 5 mA cm^{-2}@2.5 mAh cm^{-2}.

(II) Verification of SEI stability

To further verify whether the SEI derived from the ZS+TEABF electrolyte can maintain its structural and chemical stability during long-term cycling, a series of additional characterization experiments were conducted as follows:

Firstly, in-situ DRT analysis was carried out on the long-cycle zinc symmetric battery in ZS+TEABF electrolyte. The results indicated that the impedance of the SEI (R_{SEI}) reached its maximum value at the 10th cycle, suggesting that the SEI had not fully evolved before this point. After 30 cycles, the R_{SEI} of the battery tended to stabilize, and the interfacial charge-transfer resistance (R_{ct}) showed a high degree of consistency in subsequent cycles. This stable interfacial electrochemical behavior reflects the stability of the SEI after sufficient evolution.

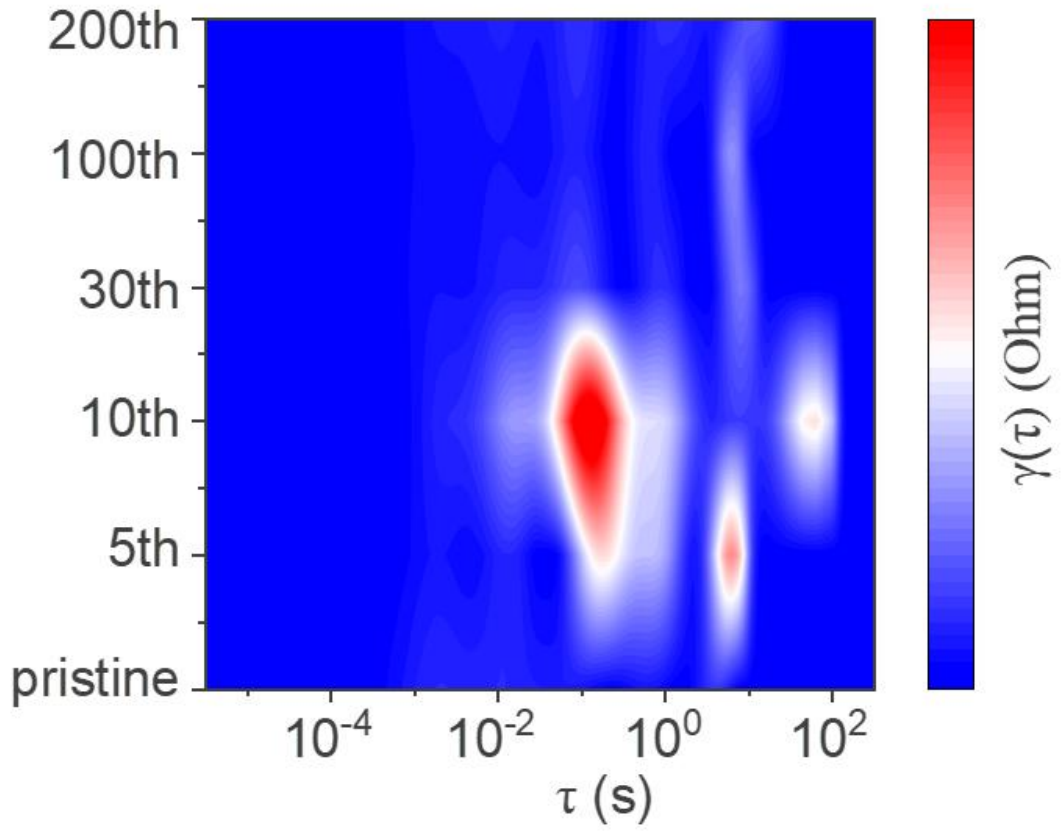

Zn||Zn symmetric battery based on in-situ DTR analysis of ZS+TEABF electrolyte.

Secondly, the surface deposition morphology of the zinc electrode after 200 cycles was characterized using SEM. Consistent with the surface features of the zinc electrode after 30 cycles in **Supplementary Fig. 1a**, there were almost no obvious by-products generated on the electrode surface, and the zinc deposition exhibited a regular texture feature, indicating that the stable SEI continuously exerted an efficient regulatory effect on the zinc deposition morphology.

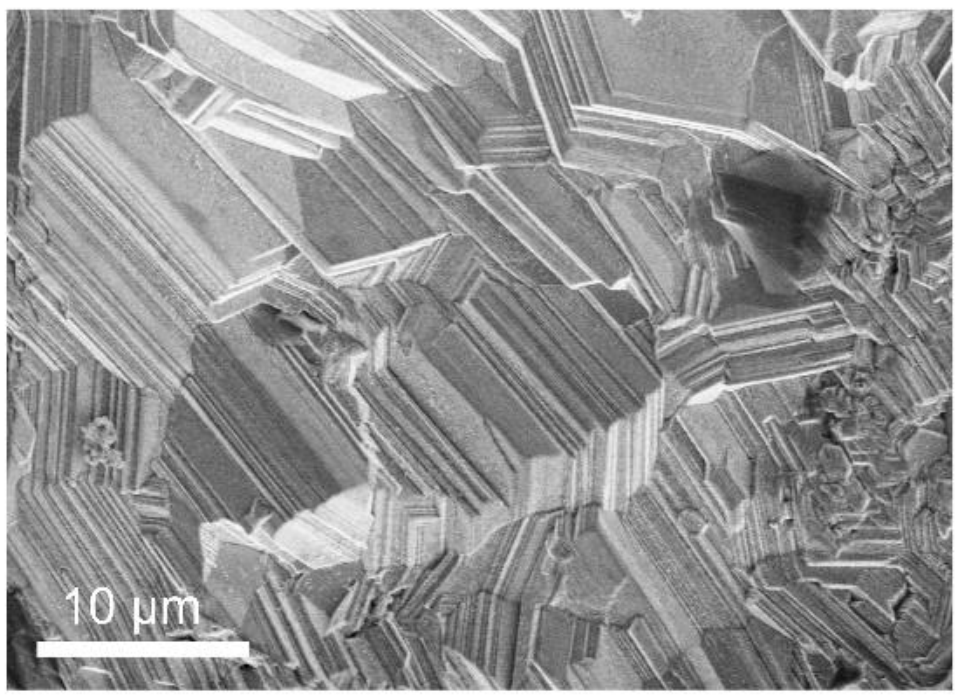

SEM image of the Zn electrode after 200 cycles at a constant current density of 5 mA cm^{-2} in ZS+TEABF electrolyte.

Additionally, HRTEM characterization was conducted after 100 cycles of zinc plating/stripping on the copper mesh substrate. The results indicated that characteristic diffraction signals of $ZnSO_3$, $ZnCO_3$, and ZnO were detected; the connection characteristics between amorphous organic components and lattice fringes could be observed at the edges. This structure was highly consistent with the HRTEM results of the short-cycle stage (**R-MS, Fig. 5g**), further validating the structural stability of the SEI.

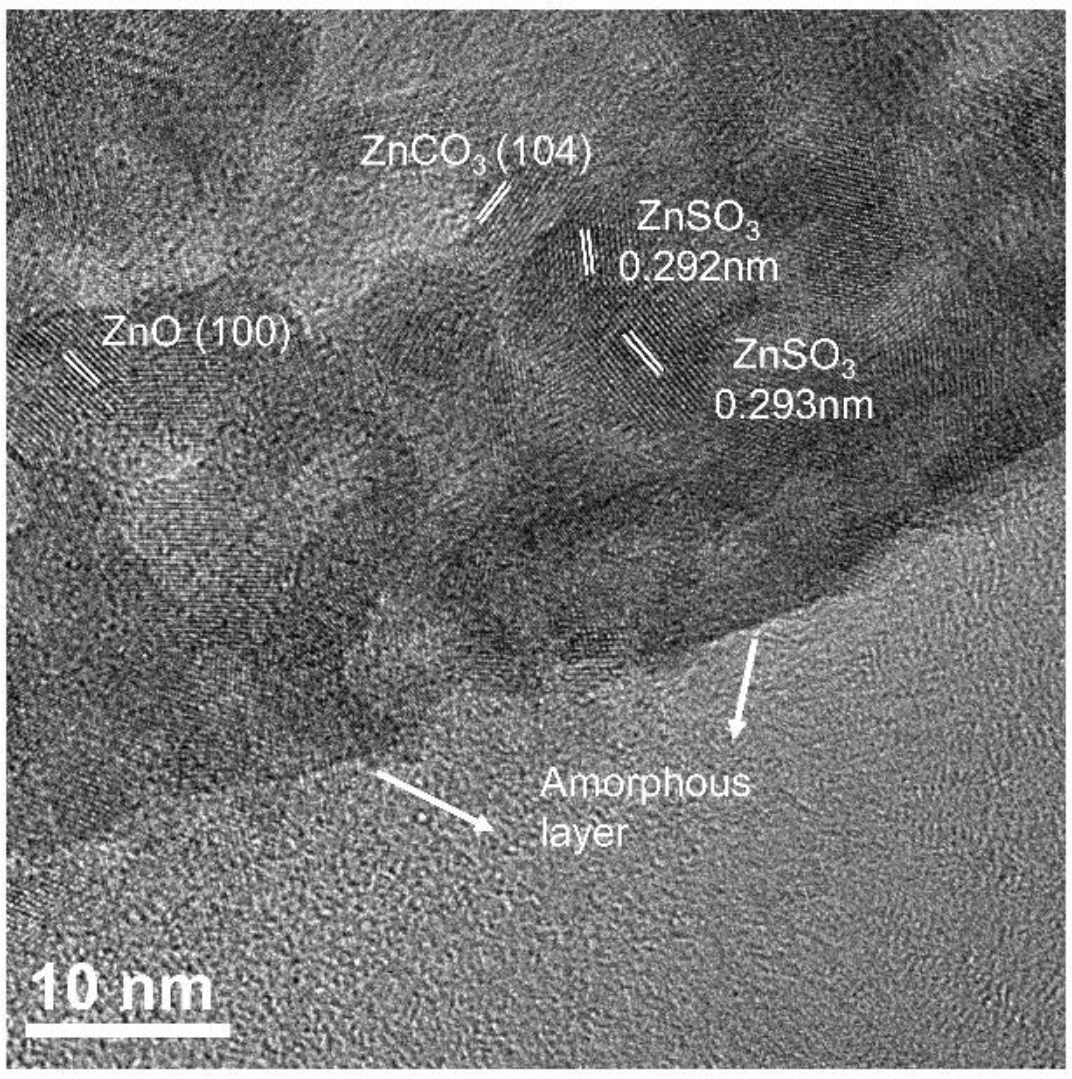

HRTEM image of after 100 cycles of zinc plating/stripping on the Cu mesh in ZS+TEABF electrolyte.

Overall, the results of the long-cycle supplementary experiments comprehensively demonstrate that the components of the SEI derived from the ZS+TEABF electrolyte possess excellent structural stability. Even after hundreds of cycles, this SEI can still maintain a favorable regulatory effect on the interfacial zinc deposition process, thereby ensuring that the Zn||Zn batteries can achieve a stable operation exceeding 850 hours under the extreme test conditions of 10 mA cm^{-2}@5 mAh cm^{-2}. Simultaneously, it verified the rigor and accuracy of our conclusion regarding the SEI.

5. The authors can also better support the SEI model using longer FFMD simulations, at the moment almost all FFMD simulations were conducted in a very short time. Probably using AIMD is a more appropriate method, as the AIMD will allow the review of molecular stability on the surface, due to the bond-breaking/forming mechanisms are captured. Nonetheless, if the FFMD are used, the better model of SEI on the surface can be achieved through longer simulation time at the interface and the results can be used to understand the migration barrier of Zn transport, statistically. This will give you better estimation in SEI from ZS-TEAHS and ZS-TEABF, rather than using controlled migration using DFT, which are limited and even the model of SEI is unrealistic.

Response:

Thank you very much for your valuable suggestions. We wholeheartedly concur with the significance of implementing a more accurate AIMD method to thoroughly investigate the evolutionary process of the bond structure. Based on this understanding, we carried out a simulation study on the aqueous environment containing $TEABF_4$ using AIMD. Throughout the 1000 fs simulation period, no substantial alteration in the bond length of the C–N bond was detected, and neither the C–C nor the C–H bonds exhibited further breakage. The interfacial reaction activity revealed by the AIMD simulation is in accordance with the outcomes of experimental characterization techniques (such as CV and ssNMR), suggesting that the TEA^+ demonstrates high stability in the aqueous electrolyte and does not undergo decomposition reactions but directly participates in the formation of the SEI.

Furthermore, despite the relative simplicity of the SEI model we employed, it is crucial to recognize that precisely analyzing the components and contents of the SEI remains an extremely challenging task at present. Even within more well-established lithium ion batteries, systematically elucidating the synergistic mechanism between the various components of the SEI and the ion migration/embedding behavior proves to be difficult. Existing studies are predominantly confined to assessing the influence of a single or a few key components on electrode performance (*Matter, 2025, 8, 102368; Energy Environ. Sci. 2023, 16, 5904; Nat. Chem. 2024, 17, 246-255*). Therefore, in this study, we primarily constructed corresponding theoretical models based on the main SEI components identified by experimental techniques such as XPS and HRTEM, and ensured the correct and stable existence form of TEA^+. Although the model is simplified, this initial exploration of comprehensively considering the collaborative effects of multiple SEI components still serves to attract greater attention to the heterogeneous structure and complex chemical behavior of the SEI in aqueous zinc batteries.

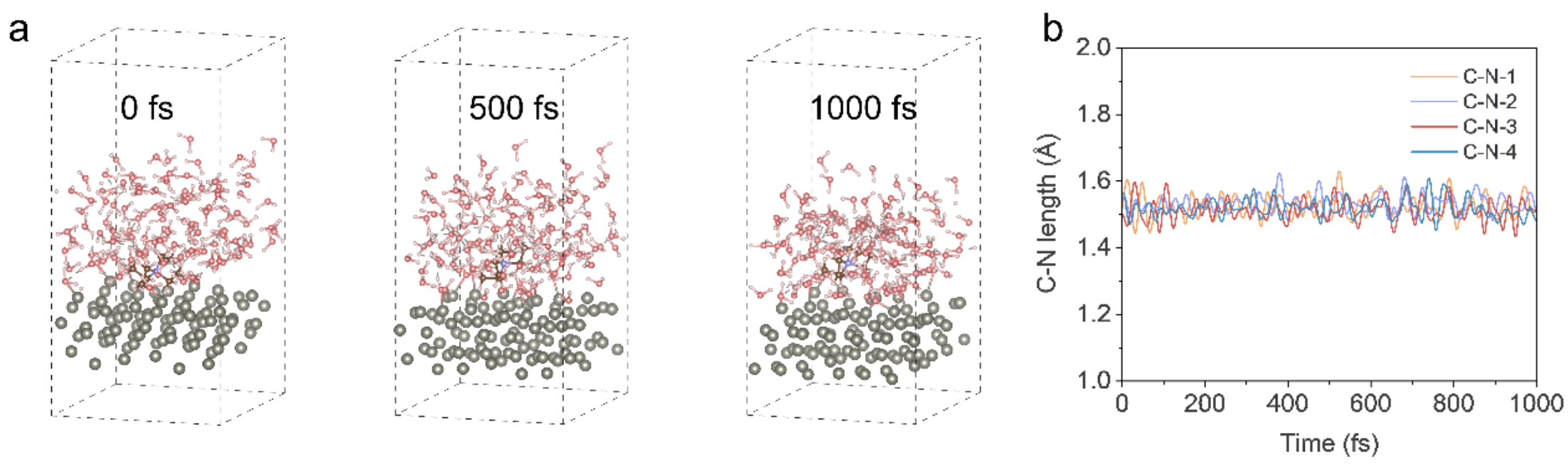

Fig. 5 | a Snapshots from AIMD simulations depicting TEA^+ existence on the Zn surface. **b** Changes in the length of C-N bond during MD simulation.

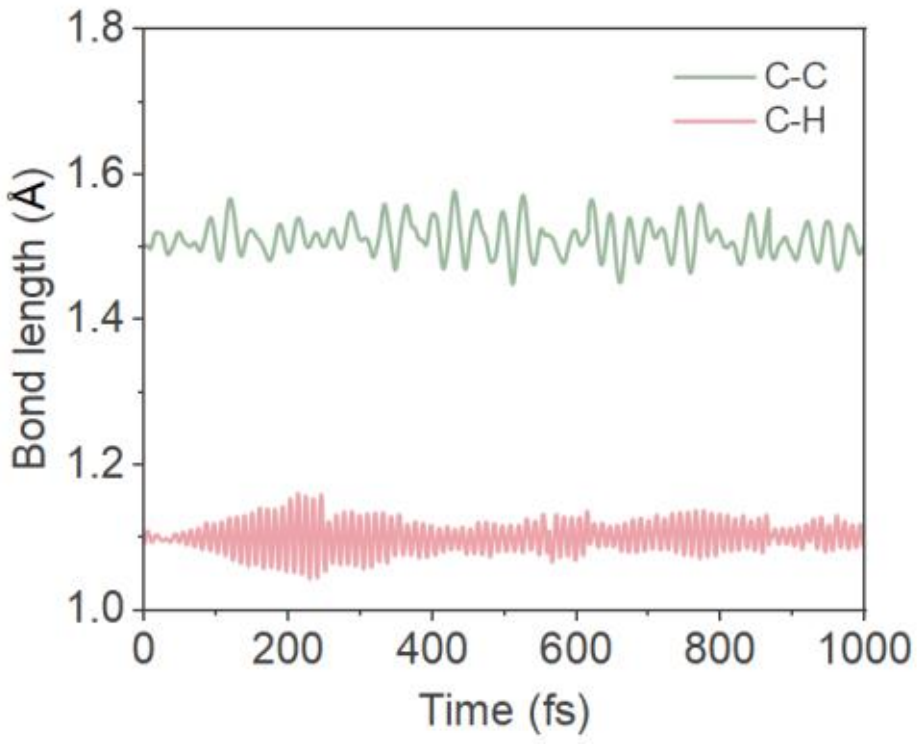

Supplementary Fig. 14. Changes in the C-C and C-H bond length during AIMD simulations.

Presented below is the description in the R-MS, accompanied by the corresponding details of AIMD.

R-MS, Page 11: '*To further investigate the dynamic behavior of TEA$^+$ at the electrode surface, ab initio molecular dynamics (AIMD) simulations were conducted using a periodic Zn crystal plane model, with particular attention to real-time variations in bond lengths of TEA$^+$ in aqueous solution (Fig. 5a). The simulation results revealed low reactivity of C–N, C–C, and C–H bonds, none of which fractured within the 1000 fs simulation period, further confirming the structural robustness of TEA$^+$ in aqueous electrolytes (Fig. 5b and Supplementary Fig. 14).*'

R-MS, Page 23: '*AIMD simulations were performed on the surface of a zinc crystal substrate in an aqueous solvent containing $TEABF_4$ salt. Initially, the geometric configuration of the system was optimized with an energy convergence criterion of 1×10^{-5} eV/atom and a maximum force tolerance of 0.0001 Å. Subsequently, MD simulations were carried out within the NVT ensemble over a total duration of 1000 fs, employing the Nosé–Hoover thermostat to maintain thermal equilibrium. The electronic structure was described using the generalized gradient approximation (GGA) with the Perdew–Burke– Ernzerhof (PBE) functional, and the equations of motion were integrated using a time step of 1 fs. The evolution of specific chemical bonds was monitored by computing instantaneous bond lengths throughout the simulation. The resulting time-series data were analyzed to determine the distribution, mean values, fluctuations, and potential instances of significant bond elongation or dissociation.*'

Overall, the authors show a promising strategy to boost the performance of Zn batteries, however, the clear rationale on the mechanisms cannot be fully explained. The justification taken from the simulations, both FFMD and DFT, are not really backed up from the observations on experiments, and the models used are too simple. The authors need either stronger experiments or a more refined models for the simulations.

Point-by-point response

Reviewer #1:

After checking this revised manuscript, I can see that the authors have well addressed referees' comments and largely improved the quality of the paper: 1, It's much more clear for the presence of the novelty of non-solvation additives, with discussion on a broad context to stand out the proposed strategy herein. 2, More control tests along with characterization and modeling results are supplemented to make interface analysis (particularly EDL) and mechanistic explanation convincing. 3, Demonstration of full cells are provided to show the considerable applicability of TEAHS and TEABF electrolyte additives. 4, More experimental and simulation details are described to enrich the information. Overall, I admire the authors' sincere efforts to strength this piece of elegant work. I thus recommend publication of the updated version as it is.

Response:
We sincerely thank the reviewer for the comprehensive and positive evaluation of our work. Your careful assessment and generous affirmation are highly appreciated, and we will continue to make efforts in relevant research.

Reviewer #2:

Thanks for the revision. it is ok to accept.

Response:
We greatly appreciate the reviewer's evaluation of our revised work and your recommendation for publication in *Nature Communications*.

Reviewer #3:

Thanks for addressing the previous comments. I really appreciate your response on the issues on clarifying mechanisms.

I would like further clarification on the additional AIMD simulations conducted. What software are you using? Any details on the set-up? Can the authors provide details how the structure is developed, and at what temperature the simulation was conducted?

It is important because the stability of your adsorbate can be highly influenced either by simulation time or temperatures. If the authors are limited with computational resources, increasing simulation temperature may be able to capture faster kinetics. At the moment, simulations conducted for 1 ps (very short) compared to other studies with

longer simulation time up to 3-5 ps, and no information on temperature. Also, please explain the set-up of supercell, how many layers of Zn, and how many waters, and why you introduce certain configuration of TEA. Please carefully address this.

Response:

We express our sincere gratitude to the reviewers for raising these detailed concerns regarding this section. The detailed supplementation of the methods of AIMD is essential for enhancing the comprehensiveness of our work.

We carried out AIMD analysis using the CASTEP module. A periodic slab model (18.5*16.0 Å) was constructed, comprising a Zn crystal surface ((002) plane, 3-layer) in contact with an explicit aqueous solution (120 H_2O molecules, the density was set to ~1.0 g/cm^3) containing the organic molecule of TEA. This setup yields a total simulation cell height of ~32 Å, including a 15 Å vacuum gap above the liquid to suppress spurious dipole interactions. The system geometry was first optimized with an energy convergence criterion of 1×10^{-5} eV/atom and a maximum force tolerance of 0.0001 Å. Subsequently, molecular dynamics simulation was propagated in the NVT ensemble for a total duration of 1000 fs, employing the Nosé thermostat to maintain the system (the simulation was performed at 298 K).

We fully acknowledge the reviewer's concern regarding simulation time and temperature effects. While previous studies (*Nat. Chem. 2025, 17, 246–255; PNAS 2024, 121, e2313096121*) suggest that a 1 ps timescale can already capture decomposition events if they occur rapidly, we recognize that longer simulations provide greater confidence. Following the reviewer's valuable suggestion, we have extended the AIMD simulation time to 4.5 ps. Throughout this extended simulation period, no bond cleavage or abnormal elongation was observed, and all key bonds of TEA remained intact. These results are consistent with our initial conclusion.

We have also made modifications and enhanced the relevant descriptions in the revised manuscript (R-MS).

R-MS, Page 11: '*The simulation results demonstrated that within 4500 fs simulation time, the C–N, C–C, and C–H bonds remained intact, indicating that TEA is structurally stable in aqueous electrolytes (Fig. 5b and Supplementary Fig. 13).*'

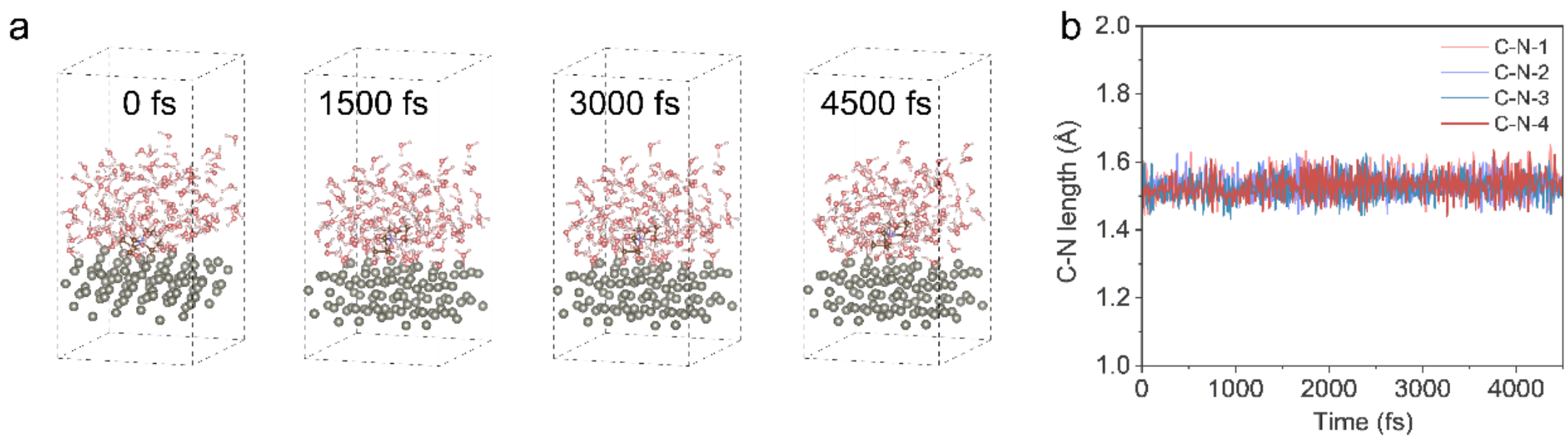

Fig. 5 a Snapshots from AIMD simulations depicting TEA existence on the Zn surface. **b** Changes in the length of C-N bond during MD simulation.

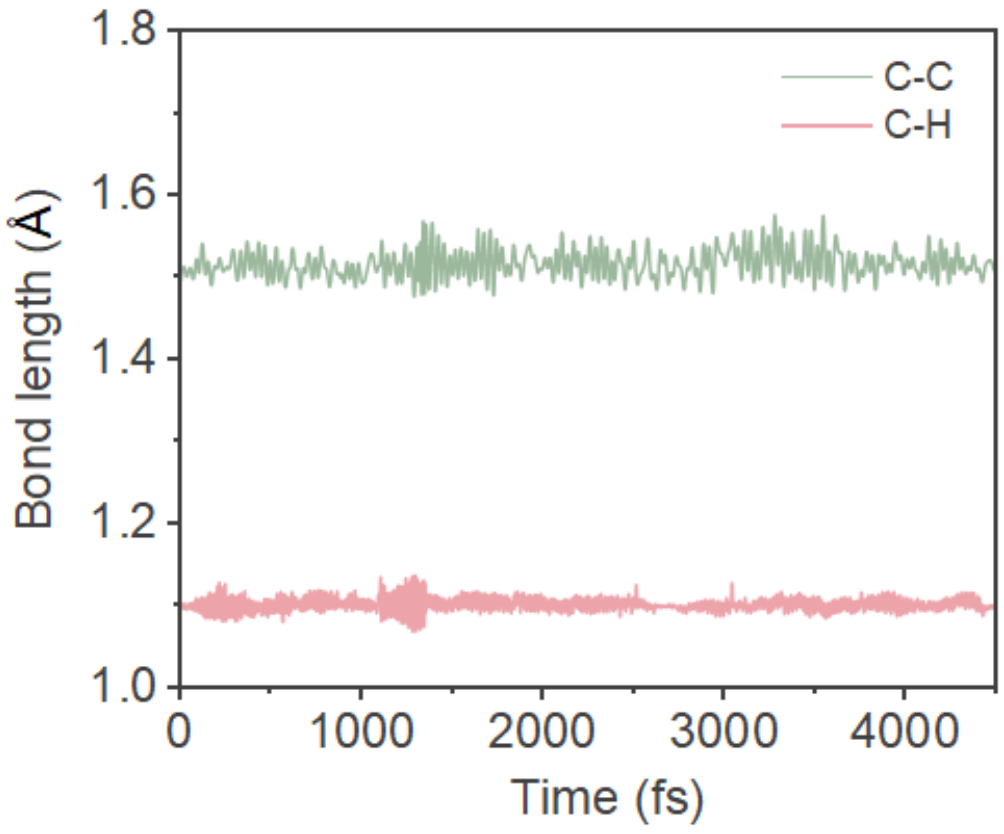

Supplementary Fig. 13. Changes in the C-C and C-H bond length during AIMD simulations.

R-MS, Page 23: '*AIMD simulations were performed on the surface of a zinc crystal substrate in an aqueous solvent containing TEABF$_4$ salt by utilizing the CASTEP module*[49]*. A periodic slab model (18.5×16.0 Å) was established, which incorporated the zinc crystal surface ((002) plane, 3 layers) in contact with an explicit aqueous solution (120 H_2O molecules, the density was set to ~ 1.0 g/cm^3) containing the target organic molecule. The total height of the simulation cell was approximately 32 Å, with a 15 Å vacuum layer above the liquid surface to mitigate spurious dipole interactions. Initially, the system geometry was optimized with an energy convergence criterion of 1×10^{-5} eV/atom and a maximum force tolerance of 0.0001 Å. Subsequently, molecular dynamics simulations were conducted in the NVT ensemble for a total duration of 1000 fs, employing the Nosé thermostat to maintain the system (simulations were performed at 298 K)*[50]*. The electronic structure was described using the GGA with the PBE functional, and the equations of motion were integrated using a time step of 1 fs. The evolution of specific chemical bonds was monitored by computing instantaneous bond lengths throughout the simulation. The resulting time-series data were analyzed to determine the distribution, mean values, fluctuations, and potential instances of significant bond elongation or dissociation.*'

The reason for focusing specifically on TEA is that we aim to determine the exact organic components of the SEI on the zinc surface. Since TEA is the original organic additive used in this work, we have performed corresponding simulations and experimental characterizations to examine its potential decomposition behavior.

Thank you once again for your comprehensive and valuable suggestions on this section. We earnestly anticipate your further guidance!
